# Design, Synthesis, and Biological Evaluation of 2-Substituted Aniline Pyrimidine Derivatives as Potent Dual Mer/c-Met Inhibitors

**DOI:** 10.3390/molecules29020475

**Published:** 2024-01-18

**Authors:** Daowei Huang, Ying Chen, Jixia Yang, Bingyang Zhao, Shouying Wang, Tingting Chai, Jie Cui, Xiaolei Zhou, Zhenhua Shang

**Affiliations:** 1 School of Chemical and Pharmaceutical Engineering, Hebei University of Science and Technology, Shijiazhuang 050018, China; huangdaowei321@163.com (D.H.); 15631196861@163.com (Y.C.); yang9910152021@163.com (B.Z.); 18633541881@163.com (T.C.); 2 State Key Laboratory Breeding Base-Hebei Key Laboratory of Molecular Chemistry for Drug, Shijiazhuang 050018, China; 3 School of Pharmacy, Hebei University of Chinese Medicine, Shijiazhuang 050200, China; jxyang_18@163.com; 4 School of Food Science and Biology, Hebei University of Science and Technology, Shijiazhuang 050018, China; 13292299239@163.com; 5 Department of Head and Neck Surgery, National Cancer Center/National Clinical Research Center for Cancer/Cancer Hospital & Shenzhen Hospital, Chinese Academy of Medical Sciences and Peking Union Medical College, Shenzhen 518116, China; cuijie@cicams-sz.org.cn

**Keywords:** Mer kinase, c-Met kinase, dual inhibitor, 2-substituted aniline pyrimidine, anticancer

## Abstract

Mer and c-Met kinases, which are commonly overexpressed in various tumors, are ideal targets for the development of antitumor drugs. This study focuses on the design, synthesis, and evaluation of several 2-substituted aniline pyrimidine derivatives as highly potent dual inhibitors of Mer and c-Met kinases for effective tumor treatment. Compound **18c** emerged as a standout candidate, demonstrating robust inhibitory activity against Mer and c-Met kinases, with IC_50_ values of 18.5 ± 2.3 nM and 33.6 ± 4.3 nM, respectively. Additionally, compound **18c** displayed good antiproliferative activities on HepG2, MDA-MB-231, and HCT116 cancer cells, along with favorable safety profiles in hERG testing. Notably, it exhibited exceptional liver microsomal stability in vitro, with a half-life of 53.1 min in human liver microsome. Compound **18c** also exhibited dose-dependent cytotoxicity and hindered migration of HCT116 cancer cells, as demonstrated in apoptosis and migration assays. These findings collectively suggest that compound **18c** holds promise as a dual Mer/c-Met agent for cancer treatment.

## 1. Introduction

Mer kinase, belonging to the Tyro3-Axl-Mer family, interacts with growth arrest-specific 6 as its biological ligand [1,2,3]. This interaction mediates platelet aggregation and epithelial cell clearance and modulates macrophage cytokine synthesis, cell motility, and cell survival. Overexpression of Mer is identified in various human cancers, such as B- and T-cell acute lymphoblastic leukemia (ALL), non-small-cell lung cancer (NSCLC), and common pediatric malignancies [4]. In instances such as NSCLC and acute leukemia, Mer overexpression attenuates sensitivity to chemotherapy-induced apoptosis and doubles cell survival. Moreover, treating melanoma cells with a small-molecule Mer inhibitor reduces colony formation in soft agar and diminishes invasion into the collagen matrix [5]. Consequently, inhibiting Mer with small-molecule inhibitors may offer clinical benefits either alone or in combination with chemotherapeutic agents.

**Figure 1:**
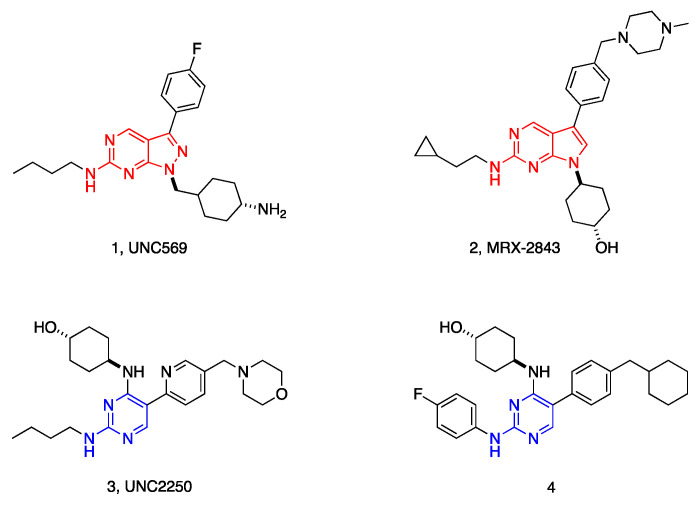
The representative Mer inhibitors.

Mer inhibitors are broadly classified into two types: aminopyrimidine pyrazole (pyrrole) and aminopyrimidine series (Figure 1). The initial small-molecule Mer inhibitor UNC569 effectively inhibits Mer and downstream signaling pathways ERK and AKT but suffers from poor pharmacokinetic properties [6,7]. Subsequent developments, such as compounds UNC2225 and MRX-2843 derived from UNC569 [8,9,10,11], exhibit enhanced inhibition of Mer signal transduction, improved pharmacokinetic properties, and favorable drug-like characteristics. Currently, MRX-2843 is in phase II clinical research for treating ALL [12]. Another notable inhibitor UNC2250 is an aminopyrimidine-type Mer inhibitor with an IC_50_ value of 1.7 nM [13,14]. It blocks the activity of the Mer-EGFR chimeric protein, retarding tumor cell growth and promoting apoptosis.

The c-mesenchymal–epithelial transition factor (c-Met) oncogene comprises 21 exons separated by 20 introns, and its protein Met/hepatocyte growth factor receptor (HGFR) [15,16], expressed in endothelial and epithelial cells, belongs to the receptor tyrosine kinases family. The c-Met kinase participates in cell proliferation, migration, invasion, survival, and branching morphogenesis [17,18]. Overexpression of c-Met has been validated as an oncogenic driver in tumorigenesis, especially in the development of invasive and metastatic phenotypes, such as NSCLC, hepatocellular carcinoma (HCC), and gastric cancer (GC). Consequently, c-Met has been acknowledged as an effective target for treating of many cancers.

**Figure 2:**
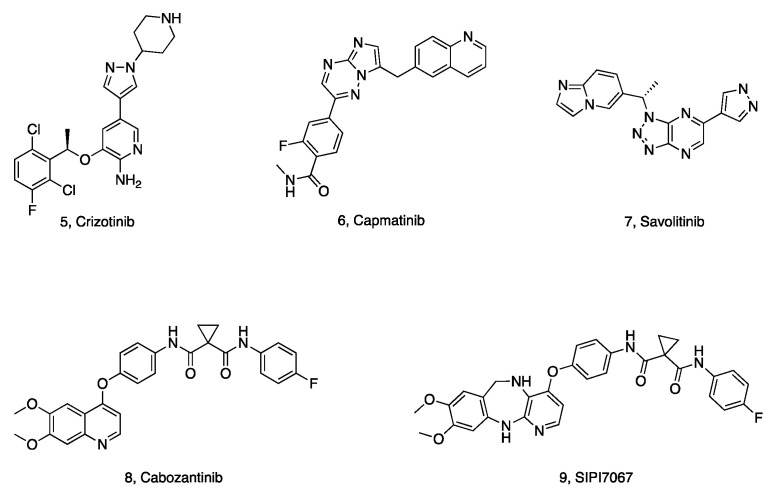
The representative c-Met inhibitors.

Pfizer’s crizotinib [19], approved by the FDA in 2011, targets locally advanced or metastatic NSCLC with ALK positivity. Exelixis’s multitarget inhibitor cabozantinib includes c-Met/VEGFR/Mer/Kit and is suggested for metastatic medullary thyroid cancer, advanced renal cell carcinoma post-antiangiogenic therapy, and first-line treatment for advanced renal cell tumors [20]. The highly selective c-Met inhibitor savolitinib is currently undergoing international multicenter phase III clinical trials for papillary renal cell carcinoma treatment [21]. Capmatinib has received an FDA priority review for treating metastatic NSCLC-carrying MET exon 14 jump mutation [22]. Lastly, our group is developing a compound SIPI7067, which is undergoing preclinical studies as a c-Met inhibitor [23]. The structures of representative c-Met inhibitors were showed in Figure 2.

**Figure 3:**
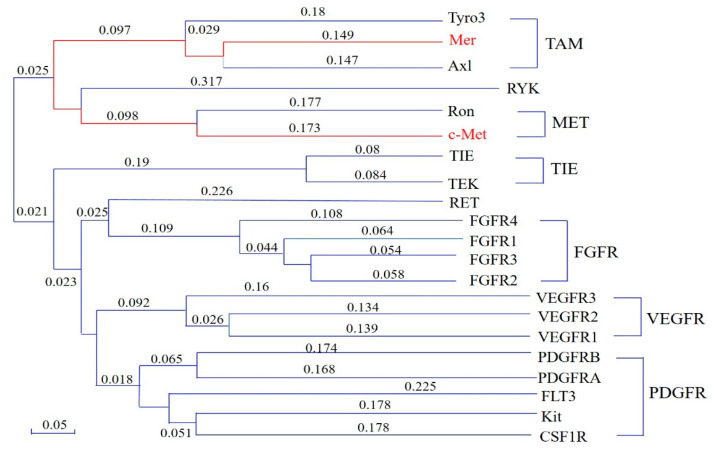
The relationships of each tyrosine kinase.

Mer and c-Met, both belonging to the receptor tyrosine kinase family, exhibit similar structures. To assess their relation, an analysis of the amino acid sequence in the kinase-binding region of human protein tyrosine kinases was conducted, resulting in a tree diagram drawn using the N-J method (Figure 3). The types of tyrosine kinases are depicted on the right side of the diagram, and the values on the lines are calculated using the Tamura–Nei method [24,25]. Larger values indicated a higher degree of evolution. Figure 3 highlights that the distance between Mer kinase and c-Met kinase is the closest (marked in red), suggesting a low degree of evolution and a close relation in the basic amino acid sequences within the kinase domain. Additionally, both Mer and c-Met receptors can activate common signaling molecules, and the two receptor families share similar functions. Consequently, concurrently targeting Mer and c-Met receptors is supported by a feasible and solid theoretical foundation.

Despite being in the early stages, the development of dual Mer/c-Met inhibitors has seen initial progress. Bicyclic pyrazolone derivatives were among the first reported dual Mer/c-Met inhibitors [26], although subsequent advancements were lacking. Exelixis’s patent (WO2019148043A1) disclosed that cabozantinib and its analogs demonstrated potent inhibitory activity against Mer kinase (IC_50_~30 nM). The binding mode of cabozantinib to Mer and c-Met receptors emphasizes the essential role of heterocyclic N atoms and amide bonds as functional groups for inhibitory activities [27]. These findings provide a scientific basis for exploring dual Mer/c-Met inhibitors.

The target compounds exhibited a binding model similar to that of cabozantinib, indicating that the 2-substituted aniline pyrimidine scaffold could serve as a building block for developing dual Mer/c-Met inhibitors. Notably, compounds **18c**, **18l**, **18n**, and **18o** displayed robust inhibitory activities against Mer and c-Met kinases. Compound **18c**, in particular, demonstrated good liver microsomal stability and exhibited potent antiproliferative activities against three cancer cell lines (HepG2, MDA-MB-231, and HCT116), comparable to or exceeding those of cabozantinib. Notably, compound **18c** demonstrated acceptable safety profiles in hERG tests, induced significant dose-dependent cytotoxicity in cancer cells (HCT116) in apoptosis assay, and inhibited cancer cell (HCT116) migration. These results position compound **18c** as a promising candidate for further research.

## 2. Results and Discussion

### 2.1. Chemistry

**Scheme 1:**
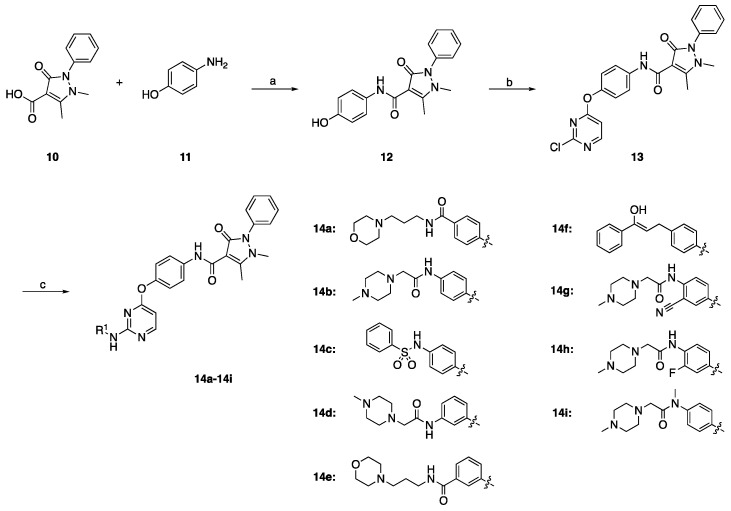
The synthetic route of compounds **14a**–**14i**. Reagents and conditions: (**a**) HBTU/TEA/DMF, r.t., 8 h; (**b**) 2,4-dichloropyrimidine, K_2_CO_3_/DMF, 80 °C, 4.5 h; (**c**) substituted anilines, PTSA/DMF, 90 °C, 4 h.

**Scheme 2:**
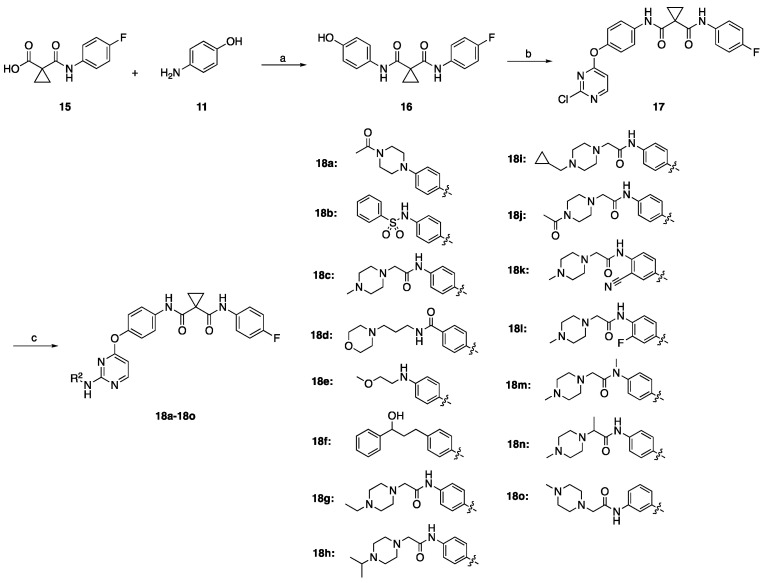
The synthetic route of compounds **18a**–**18i**. Reagents and conditions: (**a**) EDC·HCl/DMF, r.t., 6 h; (**b**) 2,4-dichloropyrimidine, K_2_CO_3_/DMF, 80 °C, 6 h; (**c**) substituted anilines, PTSA/DMF, 90 °C, 4 h.

A series of target compounds and the relative intermediates were synthesized, and the synthetic routes were shown in Scheme 1 and Scheme 2. The structures of compounds were confirmed by ^1^H-NMR, ^13^C-NMR, and HRMS spectroscopy which can be downloaded from supplementary materials.

The intermediate **12** was prepared from 4-antipyrine acid (**10**) and 4-aminophenol (**11**) through condensation reaction at room temperature [28], followed by reaction with 2,4-dichloropyrimidine to yield intermediate **13** through *SN*_2_ reaction [29]. The target compounds **14a**–**14i** were prepared from **13** and different substituted anilines [30]. The method is shown in Scheme 1.

1-((4-fluorophenyl)carbamoyl)cyclopropane-1-carboxylic acid (**15**) was firstly reacted with 4-aminophenol (**11**) to obtain intermediate **16** [31] and was treated with 2,4-dichloropyrimidine to yield the key intermediate **17**. Finally, **17** was reacted with different substituted anilines to give the desired compounds **18a**–**18o**. The method is shown in Scheme 2.

### 2.2. Kinase Inhibitory Activities

The inhibitory activities against Mer and c-Met of the designed compounds were determined. As depicted in Table 1, all tested compounds exhibited strong inhibitory activities against Mer kinase. Compounds **14a**, **14b**, and **14g** showed IC_50_ values of 7.9 ± 1.3 nM, 9.4 ± 1.5 nM, and 7.1 ± 0.9 nM, respectively. However, the inhibitory activities toward c-Met kinase were considerably weaker than those against Mer, prompting further development of compounds with dual Mer/c-Met inhibitory activities. As shown in Table 2, compound **18c** demonstrated inhibitory activities of 18.5 ± 2.3 nM and 33.6 ± 4.3 nM against Mer and c-Met kinase, respectively, showcasing robust inhibitory activities for both targets. Subsequent work focused on optimizing compound **18c**, and Table 3 reveals that compounds **18l**, **18n**, and **18o** also exhibited dual-target inhibitory activities. These results collectively indicate that the designed compounds possess potent inhibitory effects on both Mer and c-Met targets.

### 2.3. In Vitro Liver Microsomal Stability

The metabolic stability of compounds **18c**, **18l**, **18n**, and **18o** was evaluated through liver microsome assays, considering clearance and half-life measurements in human liver microsomes. As per Table 4, compound **18c** displayed a half-life and clearance of 53.1 min and 0.06 mL/min/mg, respectively, while compound **18l** exhibited 9.6 min and 0.36 mL/min/mg, respectively. For compound **18n**, the half-life and clearance were 11.9 min and 0.29 mL/min/mg, respectively, and for compound **18o**, these values were 8.6 min and 0.40 mL/min/mg, respectively. These results indicate that compound **18c** exhibits good in vitro liver microsome stability.

### 2.4. Antiproliferation Assay In Vitro

Compound **18c** was further assayed for antiproliferative activities against HepG2, MDA-MB-231, and HCT116 cancer cells using the CCK8 assay, with cabozantinib as the positive compound. As shown in Table 5, compound **18c** effectively inhibited the proliferation of HepG2, MDA-MB-231, and HCT116 cells with equal to or surpassing that of cabozantinib. Although slightly weaker against HepG2 cells, compound **18c** demonstrated superior antiproliferative activity against MDA-MB-231 and HCT116 cells. Specifically, the antiproliferative activity of compound **18c** against HepG2 cells was 3.8 times lesser than that of cabozantinib, 3.3 times higher against MDA-MB-231 cells, and 1.6 times higher against HCT116 cells. These results suggest the potential for developing compounds as novel and effective dual Mer/c-Met inhibitors.

### 2.5. Preliminary SAR Analysis

The pharmacological activities revealed that the 2-substituted aniline pyrimidine groups played pivotal roles in Mer and c-Met kinase inhibitory activities. Six different substituted anilines were incorporated as compounds **14a**–**14f**, displaying good inhibitory activities toward Mer kinase (IC_50_ = 8.1–462 nM). Notably, compounds **14a** and **14b** showed particularly strong inhibitory activities with IC_50_ values of 8.1 nM and 9.6 nM, respectively. However, the inhibitory activities of compounds **14a**–**14f** toward c-Met kinase were notably weaker than those toward Mer kinase, with IC_50_ values ranging from 144.0 to 8897.0 nM. Additionally, compounds **14g**–**14i**, derived from compound **14b**, exhibited weaker inhibitory activities toward c-Met kinase compared to Mer kinase.

The activity of the morpholine amide group equals that of the piperazine amide group (**14a**~**14b**), both surpassing the benzene sulfonamide and (Z)-1,3-diphenylprop-1-en-1-ol group (**14c** and **14f** < **14a** and **14b**). Para-substitution demonstrates superior activity compared to meta-substitution (**14a** > **14e** and **14b** > **14d**), illustrating the effective binding of para-substituted compounds with the target. No notable impact on activity was observed when benzene was substituted with cyan and fluorine groups (**14g**~**14h**).

The antipyrine side chain was substituted with the cyclopropane-1-carboxylic acid group to obtain compounds **18a**–**18f** for evaluating inhibitory activities on Mer and c-Met kinase. Compounds **18c** and **18d** exhibited potent dual inhibitory activities toward Mer and c-Met kinase with IC_50_ values of 18.9 and 33.3 nM and 41.5 and 53.2 nM, respectively. Combining the morpholine amide group and piperazine amide group with the cyclopropane-1-carboxylic acid group increased the inhibitory activity of c-Met kinase (**18c** > **14b** and **18d** > **14a**).

Compound **18c** underwent further optimization for SAR development. Substituting the piperazine group with ethyl, isopropyl, cyclopropyl methyl, and acetyl groups retained Mer inhibitory activities (**18g**–**18j**~**18c**), but the c-Met inhibitory activity decreased slightly. Similarly, the Mer inhibitory activities of compounds **18k**, **18l**, and **18n** were preserved, while the c-Met inhibitory activity decreased. Therefore, the substituted group on piperazine and benzene retained Mer inhibitory activity but decreased c-Met inhibitory activity. Methylation of the amide group decreased the activity (**18c** > **18m**), indicating the essential role of the amide group in binding affinity. The activity of the paraposition was comparable to the metaposition (**18o**~**18c**), suggesting the substituted position did not substantially affect binding affinity.

**Figure 4:**
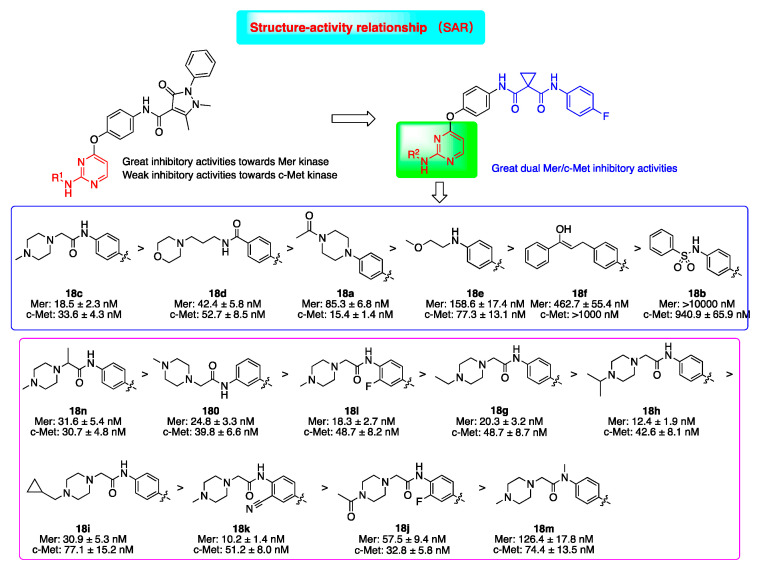
The structure–activity relationship of designed compounds.

According to SAR, the dual inhibitory activities of compounds **18c**, **18l**, **18n**, and **18o** surpassed those of other compounds, indicating strong affinities with Mer and c-Met kinase and validating the design concept. The SAR of the newly designed compounds is depicted in Figure 4.

### 2.6. Molecular Docking Study of Compound **18c**

**Figure 5:**
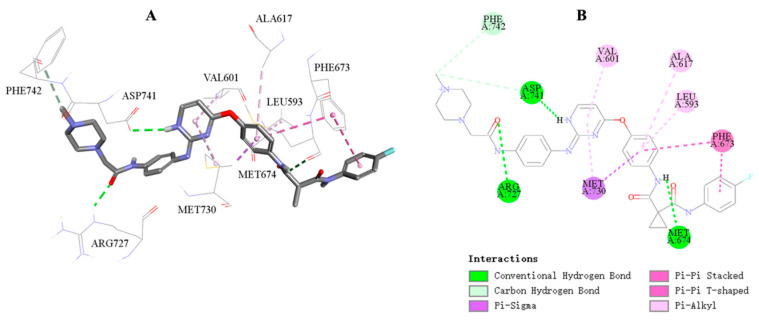
The docking mode of **18c** with Mer kinase (PDB: 4M3Q). (**A**) 3D binding mode and (**B**) 2D binding mode.

**Figure 6:**
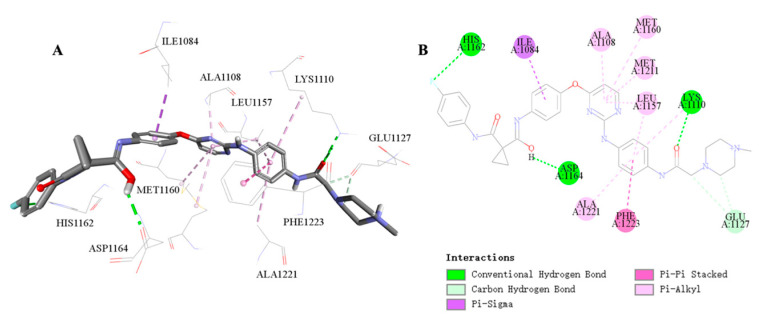
The docking mode of **18c** with c-Met kinase (PDB: 3LQ8). (**A**) 3D binding mode and (**B**) 2D binding mode.

A docking study of compound **18c** was conducted to assess the rationality of the designed strategy, and the co-crystal structure of cabozantinib with Mer and c-Met was selected as the docking mode. The docking results of compound **18c** with Mer kinase (PDB: 4M3Q) showed that the aminopyrimidine group could form a hydrogen bond with Asp741, the amide group of the side chain could form a hydrogen bond with Met674, and the amide group could form a hydrogen bond with Arg727 (Figure 5). The benzene group of the side chain could form π-π interaction. The docking results of compound **18c** with c-Met kinase (PDB: 3LQ8) showed that the amide group could form two hydrogen bonds with Asp1164 and Lys1110, respectively. The benzene group of the substituted aniline group could form π-π interaction. The docking results illustrated (Figure 6) that the designed compounds had significant inhibitory potency and the 2-substituted aniline pyrimidine group, when used as the core structure, could effectively inhibit Mer and c-Met kinase activity.

### 2.7. The hERG Tests

It is necessary to test the inhibitory activity on hERG potassium currents to further evaluate cardiotoxicity. As per the results in Table 6, the IC_50_ value of compound **18c** was >40 μM, and no obvious inhibition of hERG potassium currents was observed while Mer and c-Met kinase activity was inhibited.

### 2.8. Apoptosis Assay

HCT116 cells were grown on a coverslip, treated with various indicated doses of compound **18c** for 48 h, and stained for TUNEL (green). The number of TUNEL-positive cells was counted from 5 non-overlap random fields per group; DAPI (blue). Data are representative of three independent experiments.

**Figure 7:**
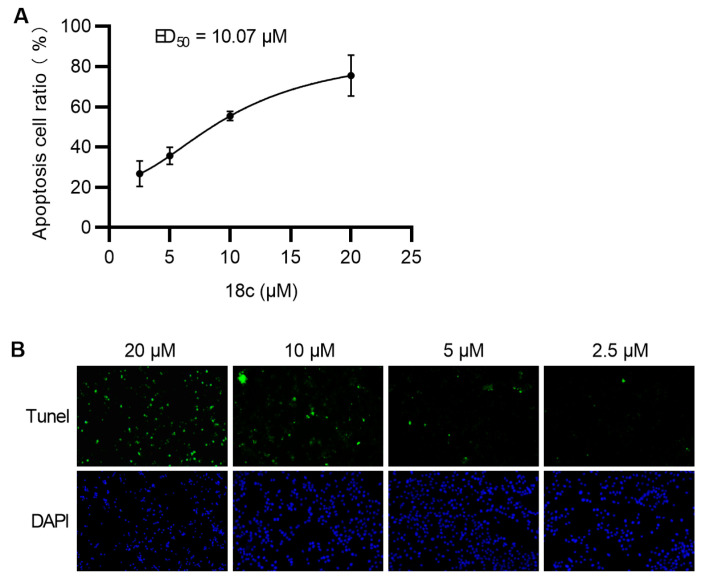
Compound **18c** induced cell apoptosis in HCT116 cancer cells. (**A**) Quantitative analysis of TUNEL staining. (**B**) Representative TUNEL and DAPI-stained images. Magnification, ×100. HCT116 cells were grown on a coverslip, treated with various indicated doses of **18c** for 48 h, and stained for TUNEL (green). The number of TUNEL-positive cells was counted from 5 non-overlap random fields per group; DAPI (blue). Data are representative of three independent experiments.

As shown in Figure 7, the TUNEL assay shows that the candidate compound **18c** can induce apoptosis of HCT116 cancer cells with an extremely low value of ED_50_ 10.07 μM, which demonstrates that **18c** is a qualified compound.

### 2.9. Transwell Assay

**Figure 8:**
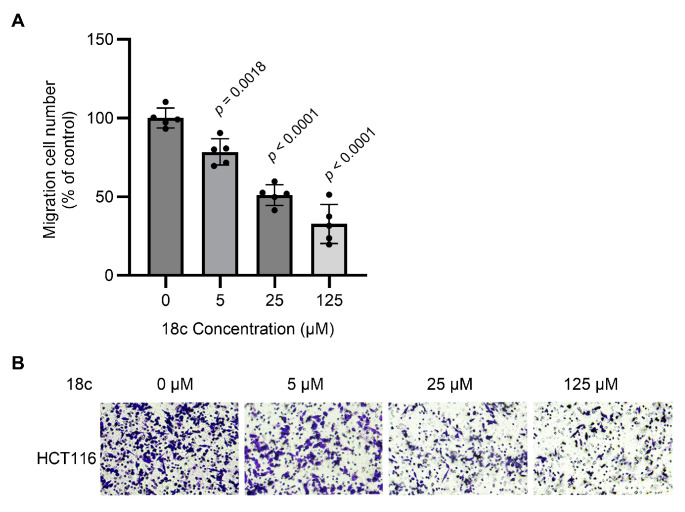
Compound **18c** inhibited cell migration in HCT116 cancer cells. (**A**) Representative images of transwell assay of HCT116 cells treated with compound **18c** for 24 h. (**B**) Quantification of transwell assay.

Transwell migration assays were performed using Transwell^®^ chamber inserts (Costar, Cambridge, MA, USA) with a porous polycarbonate membrane (8 μM pore size). As shown in Figure 8, the transwell migration assay showed that compound **18c** treatment significantly inhibited HCT116 cancer cell migration compared with the control group.

## 3. Materials and Methods

### 3.1. Chemical Part

Reactions were monitored by thin-layer chromatography (TLC) on precoated silica GF_254_ plates. High-resolution mass spectra (HRMS) were taken in ESI mode on Water Q-Tof. ^1^H-NMR and ^13^C-NMR spectra were generated on Bruker AM-400 and 500 spectrometers (Bruker Bioscience, Billerica, MA, USA) with TMS as the internal standard. All other chemicals were analytical grade and used without further purification.

#### 3.1.1. Preparation of N-(4-Hydroxyphenyl)-1,5-dimethyl-3-oxo-2-phenyl-2,3-dihydro-1H-pyrazole-4-carboxamide (Intermediate **12**)

To a solution of 4-antipyrine acid (2.0 g, 8.61 mmol) and 4-aminophenol (1.13 g, 10.33 mmol) in DMF (20 mL), HBTU (3.92 g, 10.33 mmol) and TEA (2.61 g, 25.84 mmol) were added. The reaction solution was stirred at room temperature for 8 h and monitored by TLC. The reaction solution was poured into ice water (200 mL), and the precipitate was filtered off, washed, and dried in a vacuum to yield intermediate **12** as a white solid (1.75 g, 63.0%).

#### 3.1.2. Preparation of N-(4-((2-Chloropyrimidin-4-yl)oxy)phenyl)-1,5-dimethyl-3-oxo-2-phenyl-2,3-dihydro-1H-pyrazole-4-carboxamide (Intermediate **13**)

To a solution of the intermediate **12** (1.75 g, 5.41 mmol) and 2,4-dichloropyrimidine (0.81 g, 5.41 mmol) in DMF (15 mL), K_2_CO_3_ (0.82 g, 5.95 mmol) was added. The mixture was stirred at 80 °C for 4.5 h. The reaction solution was poured into ice water (100 mL), and the precipitate was filtered off, washed, and dried in a vacuum to yield intermediate **13** as a white solid (1.85 g, 78.0%), which can be used directly without any further purification.

#### 3.1.3. General Procedure for Preparation of Compound **14a**–**14i**

To a mixture of the intermediate **13** (1.2 mmol), substituted aniline (1.0 mmol), and DMF (8 mL), p-toluenesulfonic acid (PTSA, 4.0 mmol) was added. The mixture was stirred at 90 °C for 4 h under N_2_ atmosphere. The reaction solution was cooled to room temperature, then poured into ice water (100 mL), and the precipitate was filtered off, washed, and dried in a vacuum to obtain the crude product, which was purified by silica gel chromatography using a mixture of DCM/MeOH (100:1~30:1) to afford the product **14a**–**14i**.

Compound **14a**: White solid, yield: 34.0%. ^1^H NMR (500 MHz, DMSO-d_6_) δ 10.83 (s, 1H), 9.82 (s, 1H), 8.40 (d, J = 5.5 Hz, 1H), 8.23 (s, 1H), 7.70 (d, J = 8.6 Hz, 2H), 7.67–7.56 (m, 6H), 7.52 (t, J = 7.25Hz 1H), 7.45 (d, J = 7.7 Hz, 2H), 7.22 (d, J = 8.6 Hz, 2H), 6.48 (d, J = 5.5 Hz, 1H), 3.56 (s, 4H), 3.37 (s, 3H), 3.31 (s, 1H), 3.24 (q, J = 5.7 Hz, 2H), 2.73 (s, 3H), 2.34 (s, 5H), 1.65 (s, 2H). ^13^C NMR (126 MHz, DMSO-d_6_) δ 170.07, 166.25, 163.53, 161.69, 160.39, 159.90, 154.30, 147.98, 143.26, 136.81, 133.52, 129.95, 129.33, 128.05, 127.61, 122.80, 120.71, 118.06, 99.40, 97.61, 66.64, 56.59, 53.78, 33.79, 26.44, 11.95. HRMS: *m*/*z* C_36_H_38_N_8_O_5_ [M + Na]^+^ 685.2965, found 685.2866.

Compound **14b**: White solid, yield: 12.0%. ^1^H NMR (500 MHz, DMSO-d_6_) δ 10.81 (s, 1H), 9.53 (s, 1H), 8.32 (d, J = 5.6 Hz, 1H), 7.69 (d, J = 8.9 Hz, 2H), 7.60 (t, J = 7.7 Hz, 2H), 7.54–7.49 (m, 4H), 7.46–7.43 (m, 2H), 7.37 (d, J = 8.7 Hz, 2H), 7.20 (d, J = 8.9 Hz, 2H), 6.37 (d, J = 5.6 Hz, 1H), 3.37 (s, 3H), 3.08 (s, 2H), 2.72 (s, 3H), 2.53 (s, 4H), 2.50 (s, 2H), 2.26 (s, 3H), 1.24 (s, 2H). ^13^C NMR (126 MHz, DMSO-d_6_) δ 170.00, 168.13, 163.53, 161.65, 160.31, 160.18, 154.32, 148.02, 136.71, 136.38, 133.51, 133.00, 129.95, 129.31, 127.56, 122.76, 120.63, 120.24, 119.55, 98.43, 97.63, 62.12, 54.94, 53.05, 46.09, 33.80, 11.95. HRMS: *m*/*z* C_35_H_37_N_9_O_4_ [M + Na]^+^ 670.2969, found 670.2864.

Compound **14c**: White solid, yield: 32.0%. ^1^H NMR (500 MHz, DMSO-d_6_) δ 10.84 (s, 1H), 9.93 (s, 1H), 9.50 (s, 1H), 8.30 (d, J = 5.6 Hz, 1H), 7.71–7.66 (m, 4H), 7.62–7.56 (m, 3H), 7.54–7.49 (m, 3H), 7.47–7.43 (m, 2H), 7.38 (d, J = 8.4 Hz, 2H), 7.17 (d, J = 8.9 Hz, 2H), 6.82 (d, J = 8.8 Hz, 2H), 6.38 (d, J = 5.6 Hz, 1H), 3.37 (s, 3H), 2.74 (s, 3H). ^13^C NMR (126 MHz, DMSO-d_6_) δ 170.00, 163.60, 161.69, 160.27, 159.97, 154.32, 147.93, 139.97, 137.54, 136.79, 133.54, 133.14, 131.34, 129.96, 129.56, 129.32, 127.59, 127.18, 122.82, 122.09, 120.51, 119.75, 98.60, 97.66, 33.79, 11.97, 11.93. HRMS: *m*/*z* C_34_H_29_N_7_O_5_S [M + Na]^+^ 670.1951, found 670.1851.

Compound **14d**: White solid, yield: 19.0%. ^1^H NMR (500 MHz, DMSO-d_6_) δ 10.81 (s, 1H), 9.53 (d, J = 19.7 Hz, 2H), 8.34 (d, J = 5.6 Hz, 1H), 7.73 (s, 1H), 7.67 (d, J = 8.8 Hz, 2H), 7.60 (t, J = 7.6 Hz, 2H), 7.52 (t, J = 7.4 Hz, 1H), 7.45 (d, J = 7.4 Hz, 2H), 7.30 (d, J = 7.8 Hz, 1H), 7.20 (d, J = 8.8 Hz, 3H), 7.03 (d, J = 8.1 Hz, 1H), 6.39 (d, J = 5.6 Hz, 1H), 3.37 (s, 4H), 3.10 (s, 2H), 2.72 (s, 3H), 2.54 (s, 3H), 2.41 (s, 4H), 2.20 (s, 3H). ^13^C NMR (126 MHz, DMSO-d_6_) δ 170.00, 168.46, 163.51, 161.64, 160.31, 160.23, 154.27, 147.98, 140.89, 138.88, 136.69, 133.50, 129.95, 129.33, 128.82, 127.60, 122.79, 120.64, 115.13, 113.59, 110.94, 98.75, 97.56, 62.05, 55.07, 53.06, 46.15, 33.79, 11.93. HRMS: *m*/*z* C_35_H_37_N_9_O_4_ [M + Na]^+^ 670.2969, found 670.2865.

Compound **14e**: White solid, yield: 28.0%. ^1^H NMR (500 MHz, DMSO-d_6_) δ 10.81 (s, 1H), 9.67 (s, 1H), 8.36 (d, J = 5.5 Hz, 1H), 8.33 (s, 1H), 7.96 (s, 1H), 7.73 (d, J = 7.7 Hz, 1H), 7.68 (d, J = 8.7 Hz, 2H), 7.60 (t, J = 7.5 Hz, 2H), 7.52 (t, J = 7.1 Hz, 1H), 7.45 (d, J = 7.5 Hz, 2H), 7.30 (d, J = 7.4 Hz, 1H), 7.20 (d, J = 8.6 Hz, 2H), 7.16 (t, J = 7.7 Hz, 1H), 6.42 (d, J = 5.5 Hz, 1H), 3.56 (s, 4H), 3.37 (s, 3H), 3.25 (dd, J = 6.6 Hz, J = 12.2Hz, 2H), 2.73 (s, 3H), 2.35 (s, 6H), 1.67 (s, 2H). ^13^C NMR (126 MHz, DMSO-d_6_) δ 170.05, 166.93, 163.53, 161.65, 160.38, 160.16, 154.31, 147.98, 140.69, 136.73, 135.79, 133.51, 129.95, 129.32, 128.45, 127.58, 122.80, 121.91, 120.74, 120.31, 118.89, 98.96, 97.58, 66.59, 56.52, 38.21, 33.80, 31.62, 30.30, 11.94. HRMS: *m*/*z* C_36_H_38_N_8_O_5_ [M + Na]^+^ 685.3000, found 685.2864.

Compound **14f**: White solid, yield: 84.0%. ^1^H NMR (500 MHz, DMSO-d_6_) δ 10.82 (s, 1H), 9.46 (s, 1H), 8.32 (d, J = 5.5 Hz, 1H), 7.69 (d, J = 8.6 Hz, 2H), 7.60 (t, J = 7.5 Hz, 2H), 7.52 (t, J = 7.3 Hz, 1H), 7.44 (d, J = 7.3 Hz, 4H), 7.30 (d, J = 4.0 Hz, 4H), 7.19 (d, J = 8.4 Hz, 3H), 6.95 (d, J = 7.8 Hz, 2H), 6.38 (d, J = 5.5 Hz, 1H), 5.18 (s, 1H), 4.48 (t, J = 5.5 Hz, 1H), 2.72 (s, 3H), 1.81 (dt, J = 14.3, 7.9 Hz, 2H). ^13^C NMR (126 MHz, DMSO-d_6_) δ 170.08, 163.54, 161.67, 160.29, 160.20, 154.34, 148.10, 146.65, 138.29, 136.71, 135.44, 133.51, 129.95, 129.30, 128.42, 127.56, 126.24, 122.91, 120.78, 119.44, 98.29, 97.61, 72.09, 33.79, 31.30, 11.94. HRMS: *m*/*z* C_37_H_32_N_6_O_4_ [M + Na]^+^ 647.2485, found 647.2393.

Compound **14g**: White solid, yield: 9.0%. ^1^H NMR (400 MHz, DMSO-d_6_) δ 10.77 (s, 1H), 9.87 (d, J = 4.1 Hz, 2H), 8.40 (d, J = 5.6 Hz, 1H), 8.03 (s, 1H), 7.76 (s, 2H), 7.69 (d, J = 8.9 Hz, 2H), 7.60 (t, J = 7.6 Hz, 2H), 7.51 (t, J = 7.4 Hz, 1H), 7.43 (d, J = 7.4 Hz, 2H), 7.21 (d, J = 8.9 Hz, 2H), 6.50 (d, J = 5.6 Hz, 1H), 3.36 (s, 3H), 3.11 (s, 2H), 2.71 (s, 3H), 2.54 (s, 2H), 2.41 (s, 4H), 2.15 (s, 3H), 1.35–1.20 (m, 2H). ^13^C NMR (126 MHz, DMSO-d_6_) δ 170.07, 169.06, 163.52, 161.63, 160.40, 159.75, 154.44, 147.82, 137.66, 136.92, 134.24, 133.55, 129.94, 129.24, 127.45, 124.52, 123.71, 122.64, 121.54, 120.87, 117.08, 99.63,97.71, 61.51, 54.91, 53.11, 46.03, 33.82, 11.94. HRMS: *m*/*z* C_36_H_36_N_10_O_4_ [M + Na]^+^ 695.2921, found 695.2819.

Compound **14h**: White solid, yield: 29.1%. ^1^H NMR (500 MHz, DMSO-d_6_) δ 10.81 (s, 1H), 9.76 (s, 1H), 9.34 (s, 1H), 8.38 (d, J = 5.6 Hz, 1H), 7.70 (d, J = 8.8 Hz, 2H), 7.65 (d, J = 9.0 Hz, 1H), 7.60 (t, J = 7.6 Hz, 3H), 7.53 (d, J = 7.4 Hz, 1H), 7.44 (d, J = 7.4 Hz, 2H), 7.21 (d, J = 8.8 Hz, 3H), 6.48 (d, J = 5.6 Hz, 1H), 3.37 (s, 3H), 3.08 (s, 2H), 2.72 (s, 3H), 2.51 (s, 2H), 2.50 (s, 2H), 2.34 (s, 4H), 2.14 (s, 3H). ^13^C NMR (126 MHz, DMSO-d_6_) δ 170.05, 168.52, 163.51, 161.61, 159.81, 154.33, 147.90, 136.89, 133.50, 129.29, 127.50, 123.71, 122.77, 120.68, 119.37, 114.56, 106.09, 61.53, 55.14, 53.07, 46.10, 33.80, 11.95. HRMS: *m*/*z* C_35_H_36_FN_9_O_4_Na [M + Na]^+^ 688.2921, found 688.2776.

Compound **14i**: White solid, yield: 39.1%. ^1^H NMR (500 MHz, DMSO-d_6_) δ 10.81 (s, 1H), 9.72 (s, 1H), 8.36 (d, J = 5.5 Hz, 1H), 7.68 (d, J = 8.8 Hz, 2H), 7.60 (t, J = 7.6 Hz, 2H), 7.55–7.48 (m, 3H), 7.45 (d, J = 7.5 Hz, 2H), 7.20 (d, J = 8.7 Hz, 2H), 7.03 (d, J = 7.9 Hz, 2H), 6.48 (d, J = 5.2 Hz, 1H), 3.38 (s, 3H), 3.05 (s, 3H), 2.78 (s, 2H), 2.72 (s, 3H), 2.24 (d, J = 34.2 Hz, 8H), 2.09 (s, 3H). ^13^C NMR (126 MHz, DMSO-d_6_) δ 170.19, 169.12, 163.53, 161.70, 160.43, 159.91, 154.40, 148.14, 139.78, 137.07, 136.76, 133.52, 129.94, 129.29, 127.57, 127.49, 123.07, 120.94, 119.78, 98.83, 59.19, 55.05, 52.76, 33.81, 11.92. HRMS: *m*/*z* C_36_H_40_N_9_O_4_ [M + H]^+^ 662.3125, found 662.3206.

#### 3.1.4. Preparation of N-(4-Fluorophenyl)-N-(4-hydroxyphenyl)cyclopropane-1,1-dicarboxamide (Intermediate **16**)

To a solution of 1-((4-fluorophenyl)carbamoyl)cyclopropane-1-carboxylic acid (**15**, 2.02 g, 9.0 mmol) and 4-aminophenol (1.18 g, 10.8 mmol) in DMF (5 mL), EDC·HCl (2.07 g, 10.80 mmol) was added. The reaction solution was stirred at room temperature for 6 h and monitored by TLC. Ice water (125 mL) was added, and the precipitate was filtered off, washed, and dried in a vacuum to yield **16** as a white solid (2.28 g, 80.6%), which can be used for the next step without any purification.

#### 3.1.5. Preparation of N-(4-((2-Chloropyrimidin-4-yl)oxy)phenyl)-N-(4-fluorophenyl)cyclopropane-1,1-dicarboxamide (Intermediate **17**)

To a solution N-(4-((2-chloropyrimidin-4-yl)oxy)phenyl)-N-(4-fluorophenyl)cyclopropane-1,1-dicarboxamide (**16**, 2.01 g, 6.37 mmol) and 2,4-dichloropyrimidine (1.04 g, 7.01 mmol) in DMF (15 mL), K_2_CO_3_ (0.97 g, 7.01 mmol) was added under N_2_ atmosphere. The reaction solution was stirred at 80 °C for 6 h and monitored by TLC. The reaction mixture was poured into ice water (100 mL), and the precipitate was filtered off, washed, and dried in a vacuum to obtain the crude product, which was purified by silica gel chromatography using a mixture of DCM/MeOH (100:1~40:1) to afford the intermediate **17** as a white solid (2.34 g, 75.7%).

#### 3.1.6. General Procedure for Preparation of the Title Compounds **18a**–**18o**

To a mixture of the intermediate **17** (1.2 mmol), substituted aniline (1.0 mmol), and DMF (8 mL), p-toluenesulfonic acid (PTSA, 4.0 mmol) was added. The mixture was stirred at 90 °C for 4 h under N_2_ atmosphere. The reaction solution was cooled to room temperature, then poured into ice water (100 mL), and the precipitate was filtered off, washed, and dried in a vacuum to obtain the crude product, which was purified by silica gel chromatography using a mixture of DCM/MeOH (100:1~40:1) to afford the product **18a**–**18o**.

Compound **18a**: White solid, yield: 36.6%. ^1^H NMR (500 MHz, DMSO-d_6_) δ 10.16 (s, 1H), 10.09 (s, 1H), 9.36 (s, 1H), 8.28 (d, J = 5.6 Hz, 1H), 7.70 (d, J = 8.9 Hz, 2H), 7.66 (dd, J = 9.0, 5.1 Hz, 2H), 7.35 (d, J = 4.4 Hz, 2H), 7.20–7.12 (m, 4H), 6.75 (d, J = 8.3 Hz, 2H), 6.35 (d, J = 5.6 Hz, 1H), 3.55 (t, J = 4.7 Hz, 2H), 3.51 (t, J = 4.5 Hz, 2H), 3.01 (t, J = 4.6 Hz, 2H), 2.94 (t, J = 4.7 Hz, 2H), 2.01 (s, 3H), 1.51 (d, J = 8.5 Hz, 4H). ^13^C NMR (126 MHz, DMSO-d_6_) δ 170.10, 168.74 (d, J = 3.86 Hz, 1C), 168.60, 160.21 (d, J = 13.67 Hz, 1C), 159.72, 157.81, 148.68, 146.16, 136.58, 135.62 (d, J = 1.50 Hz, 1C), 133.34, 122.88 (d, J = 7.75 Hz, 1C), 122.50 (d, J = 17.70 Hz, 1C), 120.43, 116.79, 115.62, 115.45, 97.77, 50.01, 49.64, 45.99, 31.80, 21.60, 16.00. HRMS: *m*/*z* C_33_H_32_FN_7_O_4_ [M + H]^+^ 610.2500, found 610.2537.

Compound **18b**: White solid, yield: 13.5%. ^1^H NMR (500 MHz, DMSO-d_6_) δ 10.21 (s, 1H), 10.06 (s, 1H), 9.94 (s, 1H), 9.47 (s, 1H), 8.30 (d, J = 5.6 Hz, 1H), 7.70 (t, J = 9.6 Hz, 4H), 7.65 (dd, J = 8.9, 5.1 Hz, 2H), 7.59 (t, J = 7.4 Hz, 1H), 7.53 (t, J = 7.7 Hz, 2H), 7.41 (d, J = 8.3 Hz, 2H), 7.19–7.13 (m, 4H), 6.85 (d, J = 8.7 Hz, 2H), 6.36 (d, J = 5.6 Hz, 1H), 1.52 (d, J = 4.8 Hz, 4H). ^13^C NMR (126 MHz, DMSO-d_6_) δ 170.04, 168.69, 168.15, 160.32, 160.15, 159.70, 157.79, 148.52, 136.73, 136.39, 135.65 (d, J = 1.54 Hz, 1C), 132.93, 122.85 (d, J = 7.72 Hz, 1C), 122.35, 122.14, 120.31, 119.54, 115.60, 115.42, 98.37, 62.07, 54.85, 52.96, 45.95, 31.93, 15.98. HRMS: *m*/*z* C_33_H_27_FN_6_O_5_S [M + H]^+^ 639.1748, found 639.1833.

Compound **18c**: White solid, yield: 3.4%. ^1^H NMR (500 MHz, DMSO-d_6_) δ 10.15 (s, 1H), 10.10 (s, 1H), 9.49 (s, 2H), 8.32 (d, J = 5.6 Hz, 1H), 7.72 (d, J = 8.9 Hz, 2H), 7.65 (dd, J = 9.0, 5.1 Hz, 2H), 7.47 (d, J = 8.1 Hz, 2H), 7.38 (d, J = 8.8 Hz, 2H), 7.21–7.13 (m, 4H), 6.37 (d, J = 5.6 Hz, 1H), 3.05 (s, 2H), 2.51 (s, 4H), 2.42 (s, 4H), 2.20 (s, 3H), 1.51 (d, J = 10.8 Hz, 4H). ^13^C NMR (126 MHz, DMSO-d_6_) δ 169.99, 168.83, 168.65, 160.28, 160.03, 159.72, 157.81, 148.43, 139.99, 137.55, 136.77, 135.58 (d, J = 2.49 Hz, 1C), 133.15, 131.39, 129.57, 127.17, 122.94 (d, J = 7.88 Hz, 1C), 122.32, 122.08 (d, J = 10.53 Hz, 1C), 119.88, 115.60, 115.43, 98.61, 31.84, 16.02. HRMS: *m*/*z* C_34_H_35_FN_8_O_4_ [M + H]^+^ 639.2765, found 639.2849.

Compound **18d**: White solid, yield: 42.0%. ^1^H NMR (500 MHz, DMSO-d_6_) δ 10.16 (s, 1H), 10.09 (s, 1H), 9.83 (s, 1H), 8.40 (d, J = 5.6 Hz, 1H), 8.21 (t, J = 5.4 Hz, 1H), 7.72 (d, J = 8.9 Hz, 2H), 7.69–7.59 (m, 6H), 7.21 (d, J = 8.9 Hz, 2H), 7.16 (t, J = 8.9 Hz, 2H), 6.48 (d, J = 5.6 Hz, 1H), 3.56 (t, J = 4.4 Hz, 4H), 3.25 (q, J = 6.7 Hz, 2H), 2.38–2.27 (m, 6H), 1.66 (t, J = 7.05 Hz, 2H), 1.51 (d, J = 10.35 Hz, 4H). ^13^C NMR (126 MHz, DMSO-d_6_) δ 170.08, 168.77, 168.67, 166.26, 160.42, 159.87, 157.79, 148.53, 143.23, 136.77, 135.65 (d, J = 2.56 Hz, 1C), 128.10, 127.66, 122.87 (d, J = 7.82 Hz, 1C), 122.40 (d, J = 4.23 Hz, 1C), 118.07, 115.59, 115.42, 99.32, 66.67, 56.60, 53.80, 38.17, 31.95, 26.54, 15.94. HRMS: *m*/*z* C_35_H_36_FN_7_O_5_ [M + H]^+^ 654.2762, found 654.2842.

Compound **18e**: White solid, yield: 10.9%. ^1^H NMR (400 MHz, DMSO-d_6_) δ 10.07 (s, 1H), 10.00 (s, 1H), 9.02 (s, 1H), 8.16 (d, J = 5.6 Hz, 1H), 7.62 (d, J = 8.9 Hz, 2H), 7.57 (dd, J = 9.1, 5.1 Hz, 2H), 7.14 (d, J = 8.2 Hz, 2H), 7.10 (s, 2H), 7.09–7.07 (m, 2H), 6.36 (d, J = 8.5 Hz, 2H), 6.17 (d, J = 5.5 Hz, 1H), 3.36 (t, J = 5.6 Hz, 3H), 3.18 (s, 3H), 3.04 (t, J = 5.6 Hz, 2H), 1.43 (s, 4H). ^13^C NMR (101 MHz, DMSO-d_6_) δ 170.02, 168.79, 168.68, 160.45, 159.96, 157.58, 153.82, 148.64, 140.72, 136.55, 135.61 (d, J = 2.60 Hz, 1C), 122.90 (d, J = 7.91 Hz, 1C), 122.39, 122.21, 115.62, 115.40, 112.67, 97.24, 71.06, 58.45, 43.53, 32.01, 16.00. HRMS: *m*/*z* C_30_H_29_FN_6_O_4_ [M + H]^+^ 557.2234, found 557.2288.

Compound **18f**: White solid, yield: 7.4%. ^1^H NMR (400 MHz, DMSO-d_6_) δ 10.15 (s, 1H), 10.08 (s, 1H), 9.45 (s, 1H), 8.31 (d, J = 5.6 Hz, 1H), 7.70 (d, J = 8.9 Hz, 2H), 7.65 (dd, J = 9.1, 5.1 Hz, 2H), 7.43 (d, J = 8.2 Hz, 2H), 7.31 (d, J = 4.3 Hz, 4H), 7.25–7.20 (m, 1H), 7.20–7.13 (m, 4H), 6.95 (d, J = 8.4 Hz, 2H), 6.36 (d, J = 5.6 Hz, 1H), 5.21 (s, 1H), 4.51–4.46 (m, 1H), 1.88–1.76 (m, 2H), 1.48 (d, J = 3.5 Hz, 4H). ^13^C NMR (101 MHz, DMSO-d_6_) δ 170.06, 168.68 (d, J = 3.29 Hz, 1C), 160.20, 159.94, 157.55, 148.56, 146.68, 138.30, 136.71, 135.66 (d, J = 2.58 Hz, 1C), 135.44, 128.47 (d, J = 4.96 Hz, 1C), 127.09, 126.26, 122.84 (d, J = 7.87 Hz, 1C), 122.41, 122.25, 119.39, 115.63, 115.41, 98.27, 72.17, 41.54, 31.90, 31.37, 15.97. HRMS: *m*/*z* C_36_H_30_FN_5_O_4_ [M + H]^+^ 618.2438, found 618.2617.

Compound **18g**: White solid, yield: 30.3%. ^1^H NMR (400 MHz, DMSO-d_6_) δ 10.14 (s, 1H), 10.09 (s, 1H), 9.48 (s, 1H), 9.47 (s, 1H), 8.32 (d, J = 5.6 Hz, 1H), 7.71 (d, J = 8.9 Hz, 2H), 7.64 (dd, J = 9.0, 5.1 Hz, 2H), 7.47 (d, J = 8.6 Hz, 2H), 7.37 (d, J = 8.8 Hz, 2H), 7.16 (q, J = 8.7 Hz, 4H), 6.36 (d, J = 5.6 Hz, 1H), 3.04 (s, 2H), 2.50–2.39 (m, 8H), 2.33 (q, J = 7.1 Hz, 2H), 1.51 (d, J = 8.5 Hz, 4H), 0.99 (t, J = 7.2 Hz, 3H). ^13^C NMR (101 MHz, DMSO-d_6_) δ 170.05, 168.72, 168.19, 160.15, 159.95, 157.57, 148.55, 136.71, 136.39, 135.63 (d, J = 2.59 Hz, 1C), 132.93, 122.87 (d, J = 7.87 Hz, 1C), 122.35, 122.17, 120.32, 119.55, 115.62, 115.40, 98.37, 62.21, 53.24, 52.62, 52.02, 31.87, 16.01, 12.40. HRMS: *m*/*z* C_35_H_37_FN_8_O_4_ [M + H]^+^ 653.2922, found 653.3003.

Compound **18h**: White solid, yield: 13.4%. ^1^H NMR (400 MHz, DMSO-d_6_) δ 10.14 (s, 1H), 10.10 (s, 1H), 9.48 (s, 1H), 9.47 (s, 1H), 8.32 (d, J = 5.6 Hz, 1H), 7.71 (d, J = 8.9 Hz, 2H), 7.64 (dd, J = 9.0, 5.1 Hz, 2H), 7.47 (d, J = 8.7 Hz, 2H), 7.37 (d, J = 8.8 Hz, 2H), 7.16 (q, J = 8.6 Hz, 4H), 6.36 (d, J = 5.6 Hz, 1H), 3.04 (s, 2H), 2.67 (s, 1H), 2.55–2.50 (m, 8H), 1.50 (d, J = 8.4 Hz, 4H), 0.98 (d, J = 6.5 Hz, 6H). ^13^C NMR (101 MHz, DMSO-d_6_) δ 170.04, 168.72, 168.20, 160.15, 159.95, 157.56, 148.55, 136.71, 136.40, 135.63 (d, J = 2.58 Hz, 1C), 132.92, 122.87 (d, J = 8.04 Hz, 1C), 122.34, 122.17, 120.40, 119.55, 115.62, 115.40, 98.38, 78.93, 66.79, 62.19, 48.28, 31.88, 18.60, 16.00. HRMS: *m*/*z* C_36_H_39_FN_8_O_4_ [M + H]^+^ 667.3078, found 667.3160.

Compound **18i**: White solid, yield: 15.7%. ^1^H NMR (400 MHz, DMSO-d_6_) δ 10.07 (s, 1H), 10.02 (s, 1H), 9.41 (s, 1H), 9.40 (s, 1H), 8.25 (d, J = 5.6 Hz, 1H), 7.64 (d, J = 8.9 Hz, 2H), 7.57 (dd, J = 9.1, 5.1 Hz, 2H), 7.39 (d, J = 8.6 Hz, 2H), 7.30 (d, J = 8.9 Hz, 2H), 7.09 (q, J = 8.8 Hz, 4H), 6.29 (d, J = 5.6 Hz, 1H), 2.97 (s, 2H), 2.4 (d, J = 1.7 Hz, 8H), 2.12 (d, J = 6.5 Hz, 2H), 1.43 (d, J = 8.8 Hz, 4H), 1.01 (t, J = 7.2 Hz, 1H), 0.41–0.35 (m, 2H), 0.01 (d, J = 4.8 Hz, 2H). ^13^C NMR (101 MHz, DMSO-d_6_) δ 170.05, 168.72, 168.20, 160.15, 159.95, 157.56, 148.55, 136.71, 136.39, 135.63 (d, J = 2.55 Hz, 1C), 132.92, 122.87 (d, J = 7.90 Hz, 1C), 122.35, 122.17, 120.33, 119.54, 115.62, 115.40, 98.37, 63.19, 62.24, 52.96, 46.16, 31.89, 16.01, 8.67, 4.19. HRMS: *m*/*z* C_37_H_39_FN_8_O_4_ [M + H]^+^ 679.3078, found 679.3158.

Compound **18j**: White solid, yield: 34.8%. ^1^H NMR (400 MHz, DMSO-d_6_) δ 10.14 (s, 1H), 10.07 (s, 1H), 9.55 (s, 1H), 9.48 (s, 1H), 8.32 (d, J = 5.6 Hz, 1H), 7.71 (d, J = 8.9 Hz, 2H), 7.64 (dd, J = 9.1, 5.1 Hz, 2H), 7.47 (d, J = 8.7 Hz, 2H), 7.38 (d, J = 8.9 Hz, 2H), 7.20–7.12 (q, J = 8.8 Hz, 4H), 6.36 (d, J = 5.6 Hz, 1H), 3.51–3.44 (m, 4H), 3.10 (s, 2H), 2.49 (s, 2H), 2.44 (t, J = 4.8 Hz, 2H), 1.99 (s, 3H), 1.50 (d, J = 8.7 Hz, 4H). ^13^C NMR (101 MHz, DMSO-d_6_) δ 170.05, 168.72 (d, J = 5.70 Hz, 1C), 168.60, 168.05, 160.15, 159.96, 157.57, 148.54, 136.72, 136.43, 135.62 (d, J = 2.60 Hz, 1C), 132.93, 122.89 (d, J = 7.95 Hz, 1C), 122.36, 122.15, 120.42, 119.51, 115.63, 115.41, 98.36, 61.83, 53.33, 52.89, 46.04, 31.91, 21.62, 15.96. HRMS: *m*/*z* C_35_H_35_FN_8_O_5_ [M + H]^+^ 667.2714, found 667.2802.

Compound **18k**: White solid, yield: 13.9%. ^1^H NMR (400 MHz, DMSO-d_6_) δ 10.17 (s, 1H), 10.07 (s, 1H), 9.95 (s, 1H), 9.89 (s, 1H), 8.46 (d, J = 5.6 Hz, 1H), 8.02 (s, 1H), 7.87–7.74 (m, 4H), 7.69 (dd, J = 9.1, 5.1 Hz, 2H), 7.26 (d, J = 8.9 Hz, 2H), 7.21 (t, J = 8.9 Hz, 2H), 6.57 (d, J = 5.6 Hz, 1H), 3.36 (s, 2H), 3.18 (s, 2H), 2.66–2.58 (m, 6H), 2.27 (s, 3H), 1.55 (d, J = 12.0 Hz, 4H). ^13^C NMR (101 MHz, DMSO-d_6_) δ 170.10, 169.06, 168.69, 168.59, 160.45, 159.69, 157.54, 148.24, 137.62, 136.97, 135.60 (d, J = 2.62 Hz, 1C), 134.30, 124.56, 123.65, 122.80 (d, J = 7.90 Hz, 1C), 122.23 (d, J = 8.48 Hz, 1C), 121.14, 116.98, 115.65, 115.42, 104.69, 99.52, 61.55, 54.90, 53.11, 45.99, 31.85, 15.99. HRMS: *m*/*z* C_35_H_34_FN_9_O_4_ [M + H]^+^ 664.2718, found 664.2795.

Compound **18l**: White solid, yield: 10.9%. ^1^H NMR (400 MHz, DMSO-d_6_) δ 10.17 (s, 1H), 10.08 (s, 1H), 9.75 (s, 1H), 9.32 (s, 1H), 8.37 (d, J = 5.6 Hz, 1H), 7.73 (d, J = 8.9 Hz, 2H), 7.67–7.56 (m, 4H), 7.26 (d, J = 8.6 Hz, 1H), 7.21–7.13 (m, 4H), 6.45 (d, J = 5.6 Hz, 1H), 3.10 (s, 2H), 2.53 (s, 4H), 2.42 (s, 4H), 2.21 (s, 3H), 1.50 (s, 4H). ^13^C NMR (101 MHz, DMSO-d_6_) δ 170.06, 168.72, 168.65, 168.52, 160.36, 159.81, 157.55, 152.59, 148.37, 136.89, 135.62 (d, J = 2.55 Hz, 1C), 122.85 (d, J = 7.81 Hz, 1C), 122.32, 122.09, 119.43, 119.31, 115.63, 115.41, 114.62, 106.11, 99.15, 61.51, 55.01, 52.94, 45.93, 31.85, 16.02. HRMS: *m*/*z* C_34_H_34_F_2_N_8_O_4_ [M + H]^+^ 657.2671, found 657.2746.

Compound **18m**: White solid, yield: 9.4%. ^1^H NMR (400 MHz, DMSO-d_6_) δ 10.20 (s, 1H), 10.06 (s, 1H), 9.71 (s, 1H), 8.36 (d, J = 5.6 Hz, 1H), 7.69 (d, J = 8.9 Hz, 2H), 7.64 (dd, J = 9.0, 5.1 Hz, 2H), 7.55 (d, J = 8.1 Hz, 2H), 7.21–7.13 (m, 4H), 7.05 (d, J = 8.3 Hz, 2H), 6.45 (d, J = 5.6 Hz, 1H), 3.06 (s, 3H), 2.80 (s, 2H), 2.30 (s, 8H), 2.13 (s, 3H), 1.48 (d, J = 9.2 Hz, 4H). ^13^C NMR (101 MHz, DMSO-d_6_) δ 170.12, 168.75, 168.66, 160.43, 159.96, 157.56, 148.55, 136.74, 136.28, 135.63, 127.56, 122.88 (d, J = 7.74 Hz, 1C), 122.54, 122.39, 121.49, 119.80, 115.64, 115.41, 98.97, 59.20, 54.96, 52.64, 46.00, 37.30, 31.80, 15.99. HRMS: *m*/*z* C_35_H_37_FN_8_O_4_ [M + H]^+^ 653.2922, found 653.3007.

Compound **18n**: White solid, yield: 11.3%. ^1^H NMR (400 MHz, DMSO-d_6_) δ 10.20 (s, 1H), 10.13 (s, 1H), 9.59 (s, 1H), 9.53 (s, 1H), 8.38 (d, J = 5.6 Hz, 1H), 7.77 (d, J = 8.9 Hz, 2H), 7.69 (dd, J = 9.1, 5.1 Hz, 2H), 7.53 (d, J = 8.7 Hz, 2H), 7.43 (d, J = 8.9 Hz, 2H), 7.26–7.18 (m, 4H), 6.42 (d, J = 5.6 Hz, 1H), 3.54–3.46 (m, 1H), 3.23 (q, J = 6.8 Hz, 1H), 2.61 (s, 4H), 2.47 (s, 4H), 2.26 (s, 3H), 1.56 (d, J = 10.5 Hz, 4H), 1.20 (d, J = 6.8 Hz, 3H). ^13^C NMR (101 MHz, DMSO-d_6_) δ 171.15, 170.05, 168.71 (d, J = 4.87 Hz, 1C), 160.15, 159.94, 157.55, 148.52, 136.74, 136.31, 135.63 (d, J = 2.57 Hz, 1C), 133.10, 122.86 (d, J = 7.87 Hz, 1C), 122.35, 122.12, 120.20, 119.52, 115.62, 115.40, 98.36, 63.74, 56.50, 55.27, 45.96, 31.89, 16.00, 13.25. HRMS: *m*/*z* C_35_H_37_FN_8_O_4_ [M + H]^+^ 653.2922, found 653.3001.

Compound **18o**: White solid, yield: 7.6%. ^1^H NMR (400 MHz, DMSO-d_6_) δ 10.20 (s, 1H), 10.13 (s, 1H), 9.61 (s, 1H), 9.57 (s, 1H), 8.37 (d, J = 5.6 Hz, 1H), 7.76 (s, 1H), 7.74 (d, J = 9.0 Hz, 2H), 7.70 (dd, J = 9.1, 5.1 Hz, 2H), 7.36 (d, J = 8.1 Hz, 1H), 7.27–7.17 (m, 5H), 7.09 (t, J = 8.1 Hz, 1H), 6.41 (d, J = 5.6 Hz, 1H), 3.18 (s, 2H), 2.62 (s, 6H), 2.56 (s, 2H), 2.34 (s, 3H), 1.52 (s, 4H). ^13^C NMR (101 MHz, DMSO-d_6_) δ 170.01, 168.65 (d, J = 6.48 Hz, 1C), 168.37, 160.27 (d, J = 6.39 Hz, 1C), 159.94, 157.55, 148.48, 140.90, 138.88, 136.71, 135.67 (d, J = 2.70 Hz, 1C), 128.95, 122.82 (d, J = 7.91 Hz, 1C), 122.31, 122.19, 115.63, 115.41, 113.65, 110.97, 98.72, 61.70, 54.63, 52.31, 45.38, 31.98, 15.87. HRMS: *m*/*z* C_34_H_35_FN_8_O_4_ [M + H]^+^ 639.2765, found 639.2827.

### 3.2. Mer and c-Met Inhibitory Activity Assay

The in vitro inhibition assays of all final compounds against Mer and c-Met kinase were performed by Shanghai Bioduro Biological Technology Co., Ltd. (Shanghai, China). We prepared 1× buffer: HEPES 50 mM, MgCl_2_ 10 mM, EGTA 1 mM, NP-40 0.0001%, DTT 2 mM; compound dilution with DMSO. For test compounds, a 100 × final concentration solution was prepared. About 100 nL compounds were transferred to a 384-well plate by using the automated liquid handler. The final DMSO% in the assay is 1%. Enzyme stock solutions were diluted with 1× assay buffer to a concentration of 0.5 nM to make a 2× working solution. Five microliters were added manually to the assay plate (final 0.25 nM) using a multichannel pipette, spun down at 1000 rpm, centrifuged for 30 s, and incubated for 30 min at 25 °C temperature. Substrate solutions were diluted with 1× assay buffer. About 5 μL mix or buffer was added manually to the assay plate (ATP final 30 uM and TK-Sub-Biotin final 2 uM) using a multichannel pipette, spun down at 1000 rpm, and centrifuged for 30 s. After incubating at 30 °C for 60 min, 10 μL of detection solution was added to each well of the assay, mixed briefly with a centrifuge, and equilibrated for another 60 min. The luminescence was recorded using Envision.

### 3.3. Antiproliferation Assay

The antiproliferative activities of the compounds against HepG2, MDA-MB-231, and HCT116 cell lines were tested by the standard CCK-8 method [32]. Cell viability was measured by Cell Counting Kit-8 (Meilunbio, Dalian, China) according to the manufacturer’s instructions. Briefly, cancer cells were seeded into 96-well plates at a density of 2.5 × 10^3^ cells per well. After 12 h, cancer cells were treated with the indicated various concentrations of compound **18c** for 72 h. Subsequently, CCK-8 solution was added into each well and incubated for an additional 2 h. The absorbance at 450 nm was determined in each well using the BioTek Synergy HTX microplate reader. The assay was performed in triplicate, and each experiment was repeated three times. The IC_50_ for compound **18c** was calculated with the program GraphPad PRISM 9.5.

### 3.4. hERG Potassium Currents Assay

CHO cells stably expressing the transcript of hERG were investigated by the automated whole-cell patch clamp technique, using the QPatch system (Sophion, Ballerup, Denmark). The detailed procedures of the hERG potassium currents assay were described in our previous work [33]. Each cell was received from six escalating concentrations. Each concentration was tested on at least three cells. The reference compound cisapride was applied at the end of the test compound addition.

### 3.5. Liver Microsome Stability Assay

Stock solution (10 mM) of each of test compound was prepared in DMSO. The stock solution for each compound was then diluted into 200 μM with acetonitrile. Incubation mixtures were prepared in a total volume of 200 μL with final component concentrations as follows: 0.1 M PBS (pH 7.4), NADPH (2 mM), liver microsomes (0.2 mg/mL), and test compound (1 μM) or positive control. NAPDH was added after a 5 min preincubation of all other components at 37 °C, then pipette-mixed to achieve a homogenous suspension and immediately transferred 20 μL of the incubate as a 0 min sample to wells in a “Quenching” plate, followed by pipette-mixing. At 5, 15, 30, and 60 min, we pipette-mixed the incubate and serially transferred samples of 20 μL of the incubate per time point to wells in a separate “Quenching” plate, followed by pipette-mixing. In “Quenching” plates, 200 μL of acetonitrile was added with IS. The 96 wells were centrifuged at 4000 rpm for 10 min. Then 50 μL of supernatant was mixed with 50 μL of ddH_2_O and injected into the LC-MS/MS system for analysis.

### 3.6. Apoptosis Study

The One Step TUNEL Apoptosis Assay Kit (Meilunbio, Dalian, China) was used for studying apoptosis in a dose-dependent manner. HCT116 cells (1.5 × 10^4^ cells/well) were cultured in TC-treated glass coverslip-containing plates. After 24 h incubation, cells were treated with the indicated various concentrations of compound **18c** for 48 h. Next, adhered cells were fixed with 4% paraformaldehyde and permeabilized with 0.3% Triton-X-100. TUNEL staining was performed according to the manufacturer’s instructions. The nuclei of cells were counterstained with the DAPI reagent. Images were taken using an Olympus fluorescence microscope. The ED_50_ for **18c** was calculated with the program GraphPad PRISM 9.5. The assay was performed in triplicate, and each experiment was repeated three times.

### 3.7. Transwell Assay

Transwell migration assays were performed using Transwell^®^ chamber inserts (Costar, Cambridge, MA, USA) with a porous polycarbonate membrane (8 μM pore size). Briefly, 1.5 × 10^5^ HCT116 cells were added in the top chamber with FBS-free culture media containing 0.2% BSA. A culture medium containing 15% FBS was added to the bottom chamber. The indicated concentration of compound **18c** was added on both sides of the chamber insert. After incubation for 24 h at 37 °C, the cells were fixed in methanol and stained with 0.1% crystal violet. The cells that had not migrated from the top surface of the filters were removed with cotton. Five randomly selected fields were captured by brightfield microscopy for each well. Migrated cells were quantitated by ImageJ (1.53c). Experiments were repeated at least three times in replicates.

## 4. Conclusions

Herein, several novel and potent dual Mer/c-Met tyrosine kinase inhibitors were designed and synthesized. Most of the target compounds exhibited high dual inhibition potency. Appropriate docking studies identified the structure rationality of the structure due to two hydrogen-bonding interactions formed between compound **18c** and Mer and c-Met kinase. Furthermore, SAR studies led to the discovery of a dual inhibitor **18c** with excellent activity against Mer and c-Met kinases (IC_50_: 18.5 ± 2.3 nM and 33.6 ± 4.3 nM, respectively). In addition, compound **18c** showed equal or better antiproliferative activities against three cancer cell lines to positive control (cabozantinib), good liver microsome stability, and low toxicity in the hERG potassium channel assay. Finally, compound **18c** could induce apoptosis and significantly inhibit the migration of HCT116 cancer cells. To conclude, the results suggested that compound **18c** is worthy of further investigation as a dual Mer/c-Met inhibitor, and further evaluation of its performance as a drug is underway and will be reported soon.

## Figures and Tables

**Table 1:** Inhibitory activities of compound **14a**–**14i** against Mer and c-Met kinase.

Compd.	R ^1^	IC_50_ (nM) ^1^	
		Mer	c-Met
**14a**	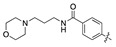	7.9 ± 1.3	151.2 ± 22.6
**14b**	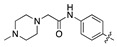	9.4 ± 1.5	179.3 ± 25.2
**14c**	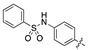	165.8 ± 21.6	>1000
**14d**	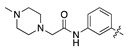	24.2 ± 2.6	143.6 ± 17.3
**14e**	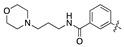	40.3 ± 3.6	321.2 ± 25.6
**14f**	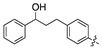	461.7 ± 41.6	>1000
**14g**	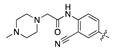	7.1 ± 0.9	89.4 ± 12.6
**14h**	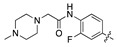	12.5 ± 2.0	74.9 ± 12.2
**14i**	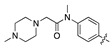	97.1 ± 12.7	224.2 ± 24.6
Cabozantinib		0.6 ± 0.1	1.4 ± 0.2

^1^ Data are means from three independent experiments in which the variation is less than 20%.

**Table 2:** Inhibitory activities of compound **18a**–**18f** against Mer and c-Met kinase.

Compd.	R ^2^	IC_50_ (nM) ^1^	
		Mer	c-Met
**18a**	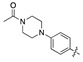	85.3 ± 6.8	15.4 ± 1.4
**18b**	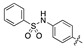	>10,000	940.9 ± 65.9
**18c**	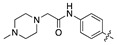	18.5 ± 2.3	33.6 ± 4.3
**18d**	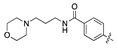	42.4 ± 5.8	52.7 ± 8.5
**18e**	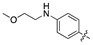	158.6 ± 17.4	77.3 ± 13.1
**18f**	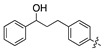	462.7 ± 55.4	>1000
Cabozantinib		0.6 ± 0.1	1.4 ± 0.2

^1^ Data are means from three independent experiments in which the variation is less than 20%. ^2^ R2 substitutions are for compounds **18a**–**18f**, and the structures of compounds **14a**–**14i** (Table 1) and **18a**–**18f** are different.

**Table 3:** Inhibitory activities of compound **18g**–**18o** against Mer and c-Met kinase.

Compd.	R ^2^	IC_50_ (nM) ^1^	
		Mer	c-Met
**18g**	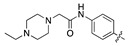	20.3 ± 3.2	48.7 ± 8.7
**18h**	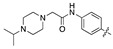	12.4 ± 1.9	42.6 ± 8.1
**18i**	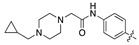	30.9 ± 5.3	77.1 ± 15.2
**18j**	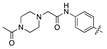	57.5 ± 9.4	32.8 ± 5.8
**18k**	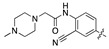	10.2 ± 1.4	51.2 ± 8.0
**18l**	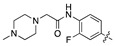	18.3 ± 2.7	48.7 ± 8.2
**18m**	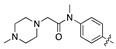	126.4 ± 17.8	74.4 ± 13.5
**18n**	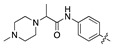	31.6 ± 5.4	30.7 ± 4.8
**18o**	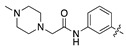	24.8 ± 3.3	39.8 ± 6.6
Cabozantinib		0.6 ± 0.1	1.4 ± 0.2

^1^ Data are means from three independent experiments in which the variation is less than 20%. ^2^ R2 substitutions are for compounds **18g**–**18o**, and the structures of compounds **14a**–**14i** (Table 1) and **18g**–**18o** are different.

**Table 4:** Microsome stability study of compound **18c**, **18l**, **18n**, and **18o**.

Cpd.	Human	
	T_1/2_ (min)	CL (mL/min/mg)
**18c**	53.1	0.06
**18l**	9.6	0.36
**18n**	11.9	0.29
**18o**	8.6	0.40
Testosterone	32.1	0.11

**Table 5:** Antiproliferative activities of compound **18c** on cancer cells.

Cpds	IC_50_(μM) ^1^ of 3 Cell Lines		
	HepG2	MDA-MB0231	HCT116
**18c**	6.63	8.90	4.95
Cabozatinib	1.72	28.96	7.82

^1^ Data are means from two independent experiments in which the variation is less than 20%.

**Table 6:** Activity on hERG potassium currents of compound **18c**.

Cpd.	IC_50_ (μM)
**18c**	>40
Cisapride	0.04

## Data Availability

The research data are available at https://www.mdpi.com/article/10.3390/molecules29020475/s1.

## Supplementary Materials

The following supporting information can be downloaded at https://www.mdpi.com/article/10.3390/molecules29020475/s1, Figures S1–S72: The spectra of ^1^H-NMR, ^13^C-NMR and HRMS.

## References

[1] Gao L., He C., Yang A., Zhou H., Lu Q., Birge R.B., Wu Y. (2023). Receptor tyrosine kinases Tyro3, Axl, and Mertk differentially contribute to antibody-induced arthritis. Cell. Commun. Signal..

[2] Myers C.K.V., de Groot A.E., Mendez S.A., Mallin M.M., Amend S.R., Pienta K.J. (2023). Targeting MerTK decreases efferocytosis and increases anti-tumor immune infiltrate in prostate cancer. Med. Oncol..

[3] Linger R.M., Cohen R.A., Cummings C.T., Sather S., Migdall-Wilson J., Middleton D.H., Lu X., Barón A.E., Franklin W.A., Merrick D.T. (2013). Mer or Axl receptor tyrosine kinase inhibition promotes apoptosis, blocks growth and enhances chemosensitivity of human non-small cell lung cancer. Oncogene.

[4] She Y.X., Xu X., Yu Q.Y., Yang X.S., He J.X., Tang X.X. (2023). Elevated expression of macrophage MERTK exhibits profibrotic effects and results in defective regulation of efferocytosis function in pulmonary fibrosis. Resp. Res..

[5] Schlegel J., Sambade M.J., Sather S., Moschos S.J., Tan A.C., Winges A., DeRyckere D., Carson C.C., Trembath D.G., Tentler J.J. (2013). MERTK receptor tyrosine kinase is a therapeutic target in melanoma. J. Clin. Investig..

[6] Sayama A., Okado K., Yamaguchi M., Samata N., Imaoka M., Kai K., Mori K. (2020). The impact of the timing of dosing on the severity of UNC569-induced ultrastructural changes in the mouse retina. Toxicol. Pathol..

[7] Koda Y., Itoh M., Tohda S. (2018). Effects of MERTK inhibitors UNC569 and UNC1062 on the growth of acute myeloid Leukaemia cells. Anticancer Res..

[8] DeRyckere D., Lee-Sherick A.B., Huey M.G., Hill A.A., Tyner J.W., Jacobsen K.M., Page L.S., Kirkpatrick G.G., Eryildiz F., Montgomery S.A. (2017). UNC2025, a MERTK small-molecule inhibitor, is therapeutically effective alone and in combination with methotrexate in leukemia models. Clin. Cancer Res..

[9] Yan D., Huelse J.M., Kireev D., Tan Z., Chen L., Goyal S., Wang X., Frye S.V., Behera M., Schneider F. (2022). MERTK activation drives osimertinib resistance in EGFR-mutant non–small cell lung cancer. J. Clin. Investig..

[10] Zhang W., DeRyckere D., Hunter D., Liu J., Stashko M.A., Minson K.A., Cummings C.T., Lee M., Glaros T.G., Newton D.L. (2014). UNC2025, a potent and orally bioavailable MER/FLT3 dual inhibitor. J. Med. Chem..

[11] Branchford B.R., Stalker T.J., Law L., Acevedo G., Sather S., Brzezinski C., Wilson K.M., Minson K., Lee-Sherick A.B., Davizon-Castillo P. (2018). The small-molecule MERTK inhibitor UNC2025 decreases platelet activation and prevents thrombosis. J. Thromb. Haemost..

[12] Lee-Sherick A.B., Jacobsen K.M., Henry C.J., Huey M.G., Parker R.E., Page L.S., Hill A.A., Wang X., Frye S.V., Earp H.S. (2018). MERTK inhibition alters the PD-1 axis and promotes anti-leukemia immunity. JCI Insight.

[13] Zhang W., Zhang D., Stashko M.A., DeRyckere D., Hunter D., Kireev D., Miley M.J., Cummings C., Lee M., Norris-Drouin J. (2013). Pseudo-cyclization through intramolecular hydrogen bond enables discovery of pyridine substituted pyrimidines as new Mer kinase inhibitors. J. Med. Chem..

[14] Shi C., Li X., Wang X., Ding N., Ping L.Y., Shi Y., Mi L., Lai Y., Song Y., Zhu J. (2018). The proto-oncogene Mer tyrosine kinase is a novel therapeutic target in mantle cell lymphoma. J. Hematol. Oncol..

[15] Gherardi E., Birchmeier W., Birchmeier C., Woude G.V. (2012). Targeting MET in cancer: Rationale and progress. Nat. Rev. Cancer.

[16] Faiella A., Riccardi F., Cartenì G., Chiurazzi M., Onofrio L. (2022). The emerging role of c-Met in carcinogenesis and clinical implications as a possible therapeutic target. J. Oncol..

[17] Lee M., Jain P., Wang F., Ma P.C., Borczuk A., Halmos B. (2021). MET alterations and their impact on the future of non-small cell lung cancer (NSCLC) targeted therapies. Expert Opin. Ther. Targets.

[18] Bouattour M., Raymond E., Qin S., Cheng A.L., Stammberger U., Locatelli G., Faivre S. (2018). Recent developments of c-Met as a therapeutic target in hepatocellular carcinoma. Hepatology.

[19] Shi Y., Chen J., Zhang H., Zhang Z., Zhang Y., Wang Z., Zhang S., Zhao J., Liu C., Wang X. (2023). Efficacy and safety of iruplinalkib (WX-0593) in ALK-positive crizotinib-resistant advanced non-small cell lung cancer patients: A single-arm, multicenter phase II study (INTELLECT). BMC Med..

[20] Hoy S.M. (2014). Cabozantinib: A review of its use in patients with medullary thyroid cancer. Drugs.

[21] Miao K., Zhang X., Wang H., Si X., Zhang L. (2023). Savolitinib versus crizotinib for treating MET positive non-small cell lung cancer. Thorac. Cancer.

[22] Dhillon S. (2020). Capmatinib: First approval. Drugs.

[23] Huang D.W., Huang L., Zhang Q.W., Li J.Q. (2017). Synthesis and biological evaluation of novel 6, 11-dihydro-5H-benzo [e] pyrimido-[5, 4-b][1, 4] diazepine derivatives as potential c-Met inhibitors. Eur. J. Med. Chem..

[24] Saitou N., Nei M. (1987). The neighbor-joining method: A new method for reconstructing phylogenetic trees. Mol. Biol. Evol..

[25] Tamura K., Nei M. (1993). Estimation of the number of nucleotide substitutions in the control region of mitochondrial DNA in humans and chimpanzees. Mol. Biol. Evol..

[26] Xi N., Wu Y.J. (2015). Preparation of Bicyclic Pyrazolone Compounds for Inhibiting or Modulating the Activity of Receptor Tyrosine Kinases.

[27] Naresh G.K., Guruprasad L. (2021). Enhanced metastable state models of TAM kinase binding to cabozantinib explains the dynamic nature of receptor tyrosine kinases. J. Biomol. Struct. Dyn..

[28] Kang S., Yim H., Won J., Kim M., Kim J., Kim H., Lee S., Yoon Y. (2008). Effective Amidation of Carboxylic Acids Using (4, 5-Dichloro-6-oxo-6H-pyridazin-1-yl)-phosphoric Acid Diethyl Ester. Bull. Korean Chem. Soc..

[29] Gao G.R., Li M.Y., Lv Y.C., Cao S.F., Tong L.J., Wei L.X., Ding J., Xie H., Duan W.H. (2016). Design, synthesis and biological evaluation of biphenylurea derivatives as VEGFR-2 kinase inhibitors (II). Chin. Chem. Lett..

[30] Park H., Jung H.Y., Mah S., Hong S. (2018). Systematic computational design and identification of low Picomolar inhibitors of Aurora kinase A. J. Chem. Inf. Model..

[31] Wei D., Fan H., Zheng K., Qin X., Yang L., Yang Y., Duan Y., Zhang Q., Zeng C., Hu L. (2019). Synthesis and anti-tumor activity of [1, 4] dioxino [2, 3-f] quinazoline derivatives as dual inhibitors of c-Met and VEGFR-2. Bioorg. Chem..

[32] Han B.Y., Liu Z., Hu X., Ling H. (2022). HNRNPU promotes the progression of triple-negative breast cancer via RNA transcription and alternative splicing mechanisms. Cell Death Dis..

[33] Huang D., Yang J.X., Zhang Q., Wang G., Zhang Z.X., Zhang Y., Li J.Q. (2021). Structure-guided design and development of novel N-phenylpyrimidin-2-amine derivatives as potential c-Met inhibitors. Eur. J. Med. Chem..

