# Supplementary material for: Design, Synthesis, and Biological Evaluation of 2-Substituted Aniline Pyrimidine Derivatives as Potent Dual Mer/c-Met Inhibitors
Source: Molecules. 2024 Jan 18;29(2):475. doi: 10.3390/molecules29020475 (PMC10819570; doi:10.3390/molecules29020475)
Supplement: Supplementary file 1 [file molecules-29-00475-s001.zip › molecules-2786503-supplementary.pdf]

Table of Contents

1. HRMS, ^{1}H-NMR, ^{13}C-NMR of compound **14a** S_1-S_3
2. HRMS, ^{1}H-NMR, ^{13}C-NMR of compound **14b** S_4-S_6
3. HRMS, ^{1}H-NMR, ^{13}C-NMR of compound **14c** S_7-S_9
4. HRMS, ^{1}H-NMR, ^{13}C-NMR of compound **14d** S_{10}-S_{12}
5. HRMS, ^{1}H-NMR, ^{13}C-NMR of compound **14e** S_{13}-S_{15}
6. HRMS, ^{1}H-NMR, ^{13}C-NMR of compound **14f** S_{16}-S_{18}
7. HRMS, ^{1}H-NMR, ^{13}C-NMR of compound **14g** S_{19}-S_{21}
8. HRMS, ^{1}H-NMR, ^{13}C-NMR of compound **14h** S_{22}-S_{24}
9. HRMS, ^{1}H-NMR, ^{13}C-NMR of compound **14i** S_{25}-S_{27}
10. HRMS, ^{1}H-NMR, ^{13}C-NMR of compound **18a** S_{28}-S_{30}
11. HRMS, ^{1}H-NMR, ^{13}C-NMR of compound **18b** S_{31}-S_{33}
12. HRMS, ^{1}H-NMR, ^{13}C-NMR of compound **18c** S_{34}-S_{36}
13. HRMS, ^{1}H-NMR, ^{13}C-NMR of compound **18d** S_{37}-S_{39}
14. HRMS, ^{1}H-NMR, ^{13}C-NMR of compound **18e** S_{40}-S_{42}
15. HRMS, ^{1}H-NMR, ^{13}C-NMR of compound **18f** S_{43}-S_{45}
16. HRMS, ^{1}H-NMR, ^{13}C-NMR of compound **18g** S_{46}-S_{48}
17. HRMS, ^{1}H-NMR, ^{13}C-NMR of compound **18h** S_{49}-S_{51}
18. HRMS, ^{1}H-NMR, ^{13}C-NMR of compound **18i** S_{52}-S_{54}
19. HRMS, ^{1}H-NMR, ^{13}C-NMR of compound **18j** S_{55}-S_{57}
20. HRMS, ^{1}H-NMR, ^{13}C-NMR of compound **18k** S_{58}-S_{60}
21. HRMS, ^{1}H-NMR, ^{13}C-NMR of compound **18l** S_{61}-S_{63}
22. HRMS, ^{1}H-NMR, ^{13}C-NMR of compound **18m** S_{64}-S_{66}
23. HRMS, ^{1}H-NMR, ^{13}C-NMR of compound **18n** S_{67}-S_{69}
24. HRMS, ^{1}H-NMR, ^{13}C-NMR of compound **18o** S_{70}-S_{72}

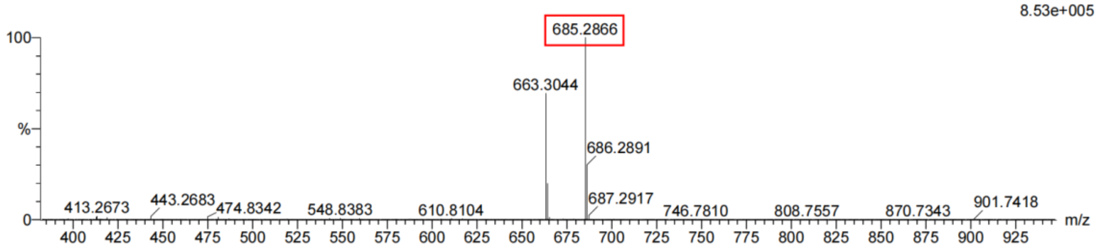

S$_1$: HRMS of compound **14a**

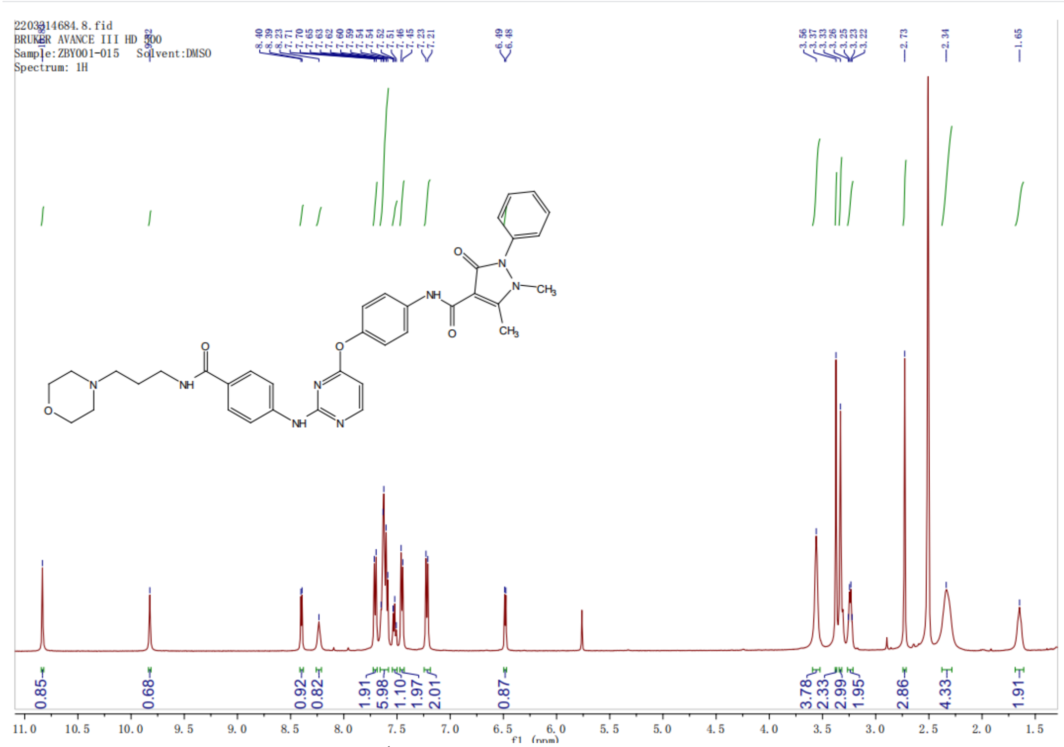

S$_2$: ^{1}H-NMR of compound **14a**

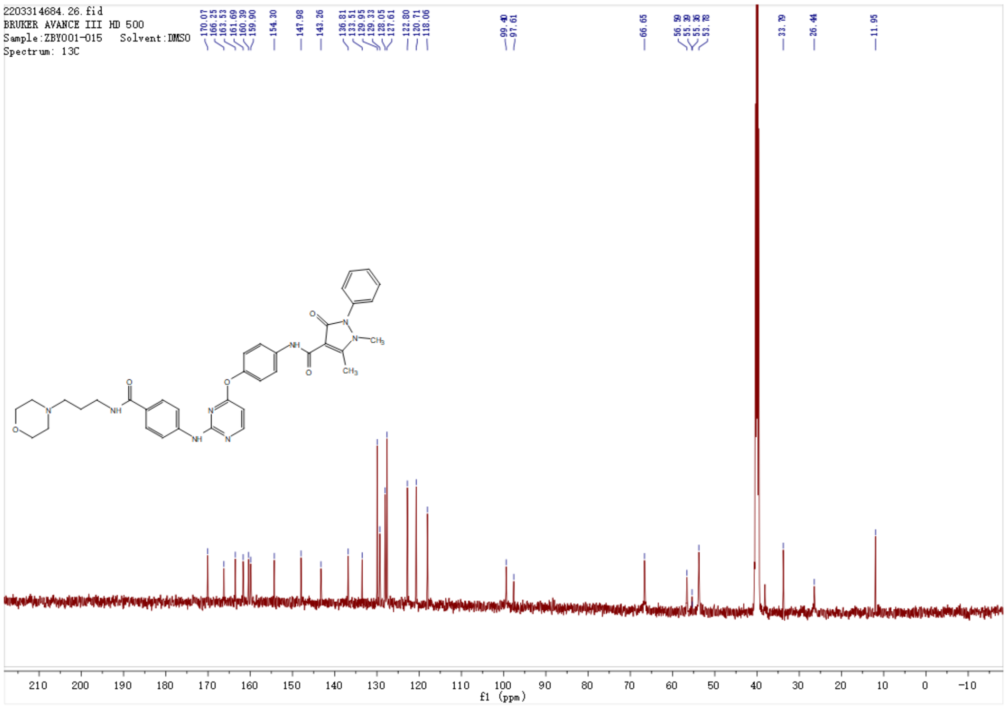

S$_3$: ^{13}C-NMR of compound **14a**

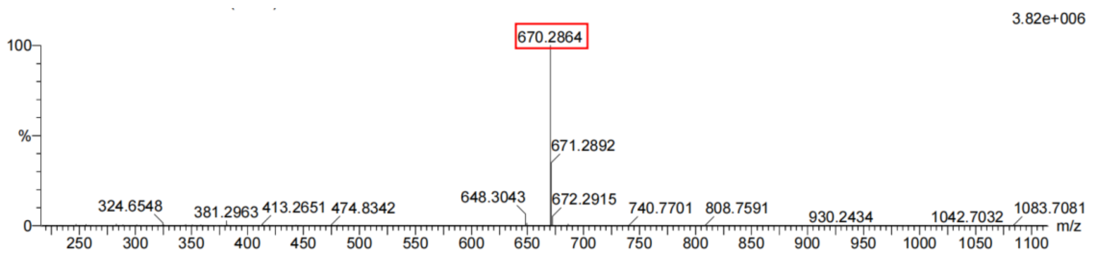

S$_4$: HRMS of compound **14b**

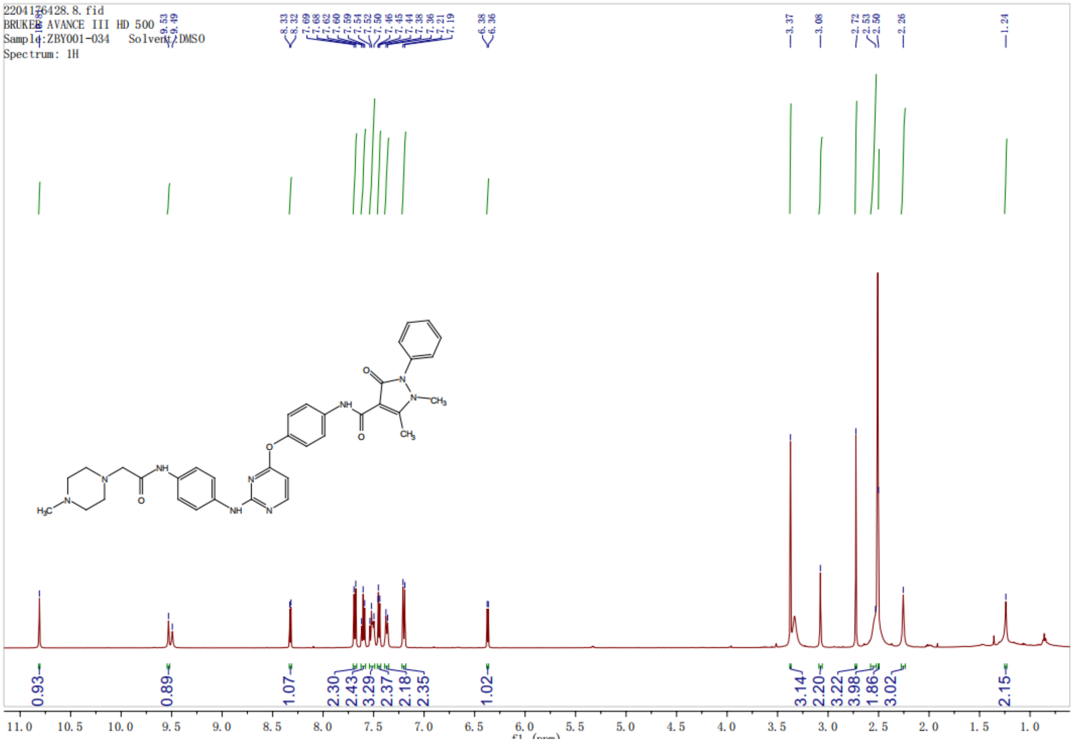

S$_5$: ^{1}H-NMR of compound **14b**

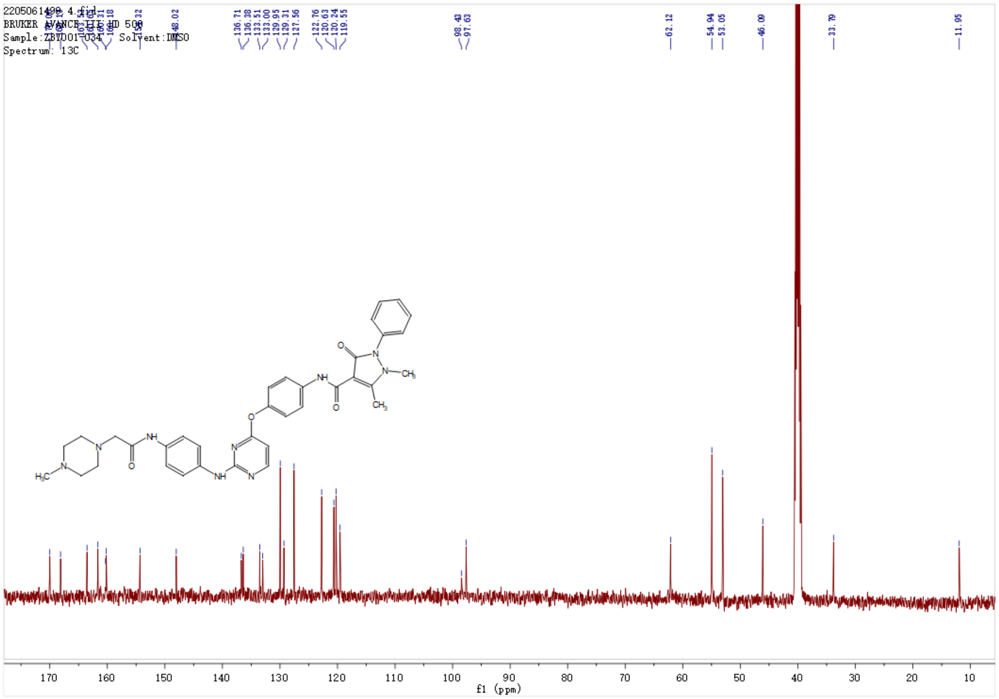

S$_6$: ^{13}C-NMR of compound **14b**

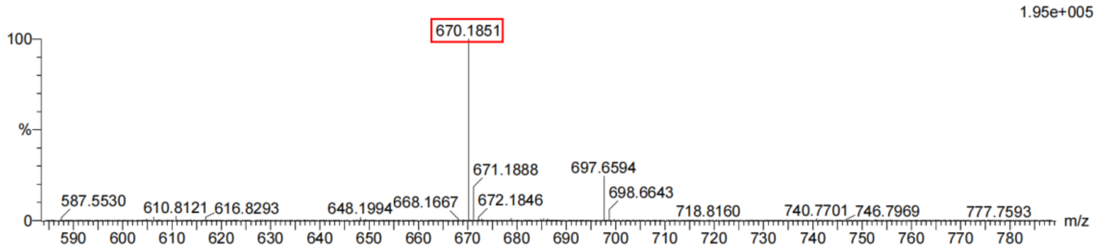

S$_7$: HRMS of compound **14c**

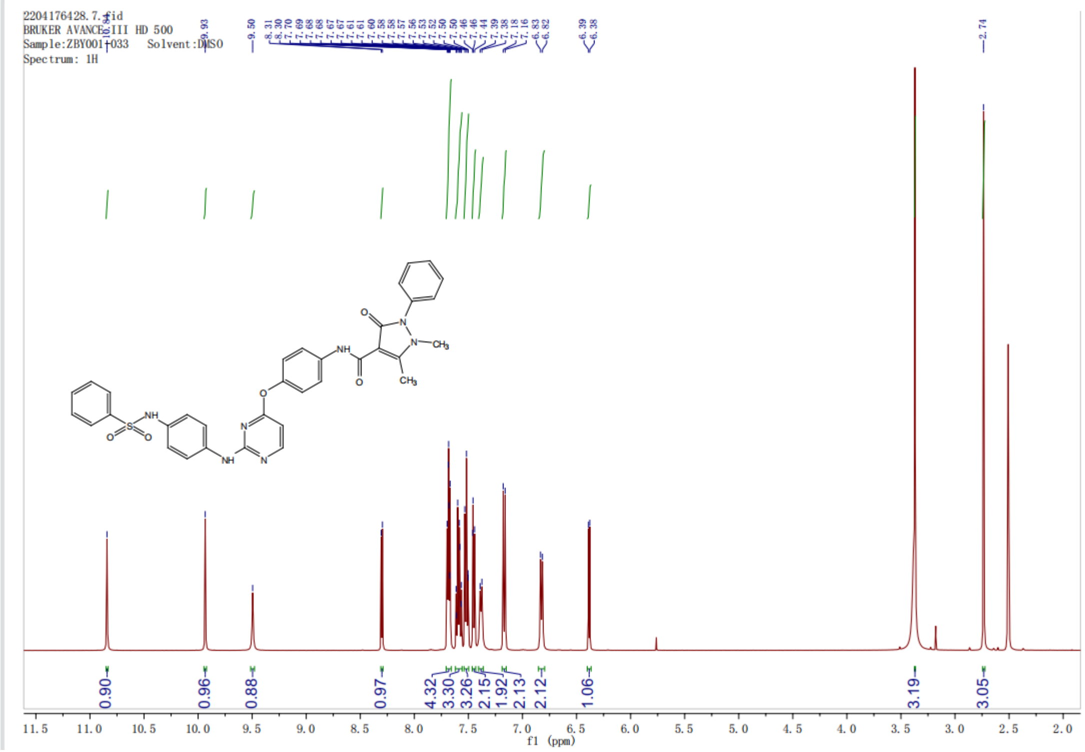

S$_8$: ^{1}H-NMR of compound **14c**

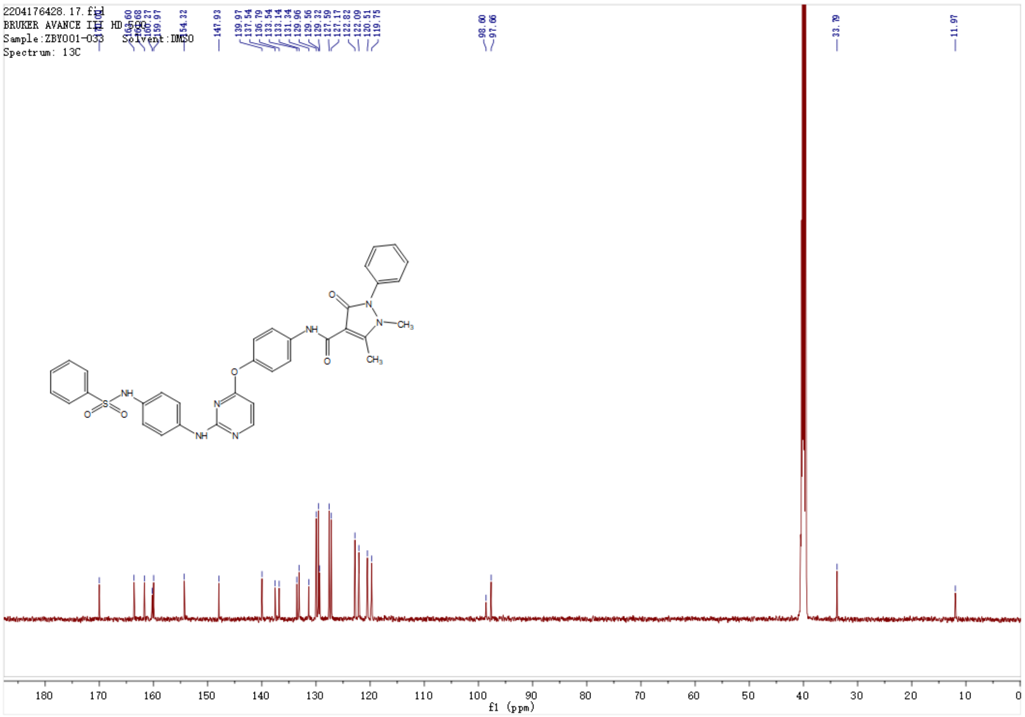

S9: ^{13}C-NMR of compound **14c**

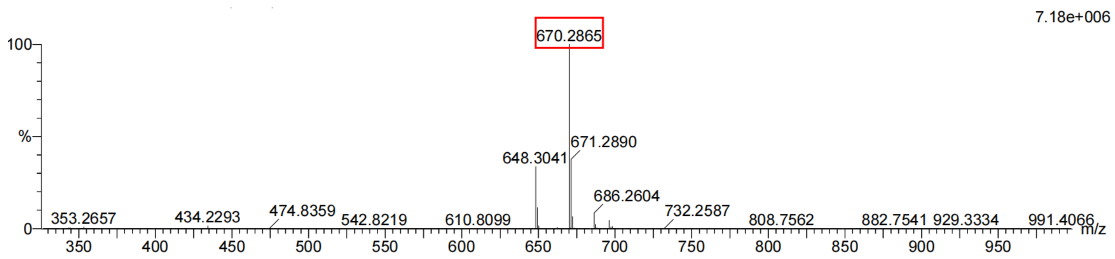

S10: HRMS of compound **14d**

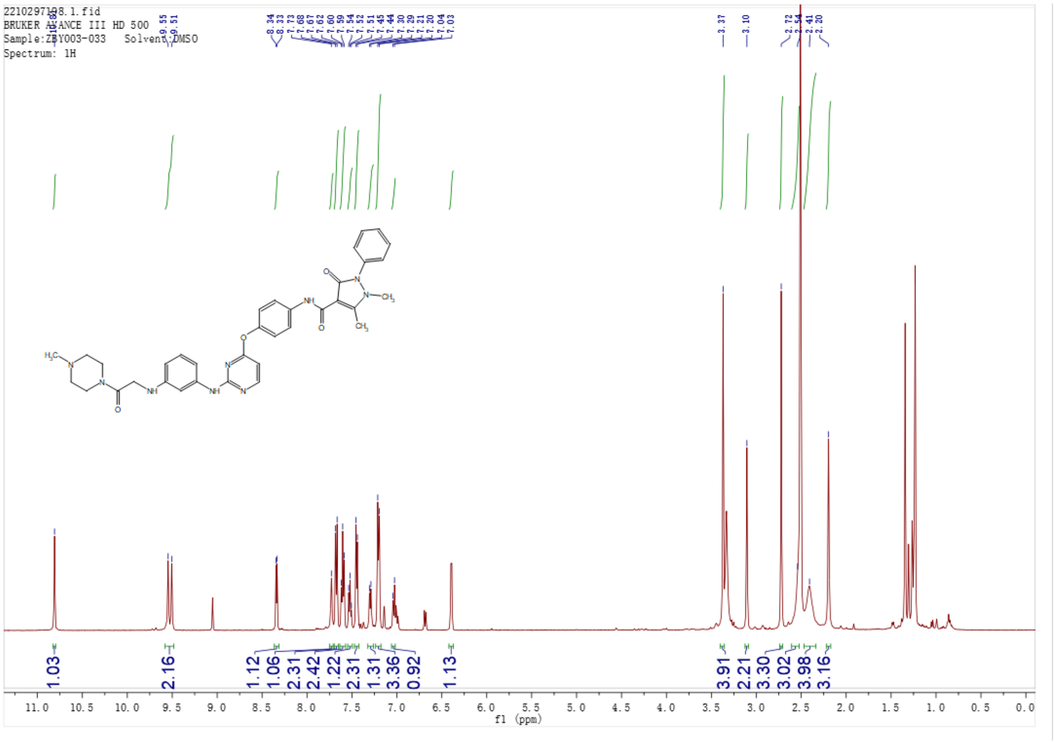

S11: ^{1}H-NMR of compound **14d**

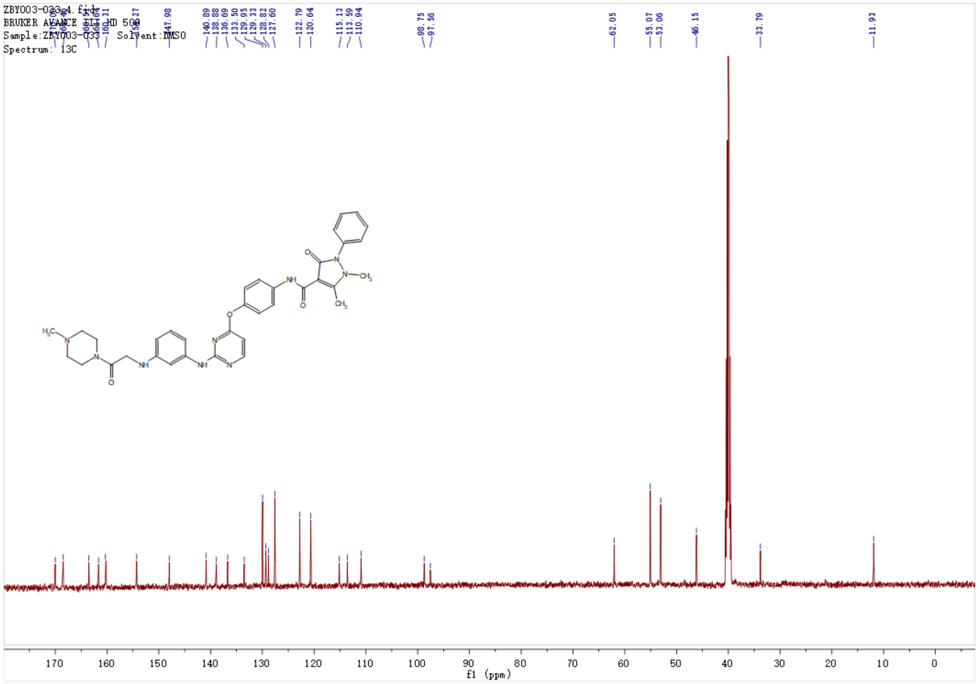

S12: ^{13}C-NMR of compound **14d**

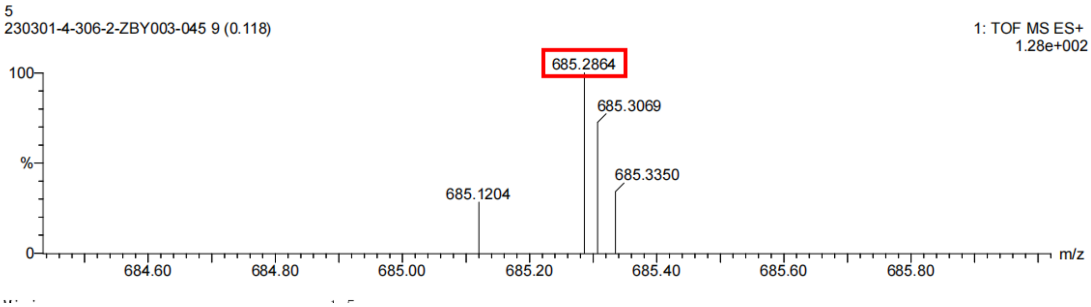

S13: HRMS of compound **14e**

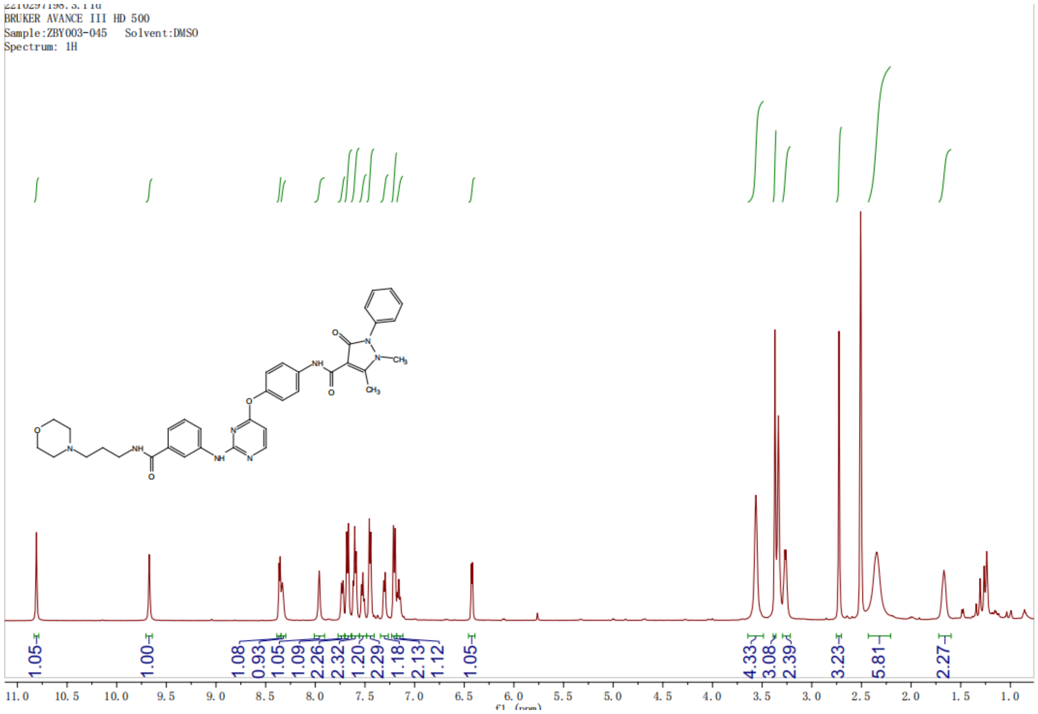

S14: ^{1}H-NMR of compound **14e**

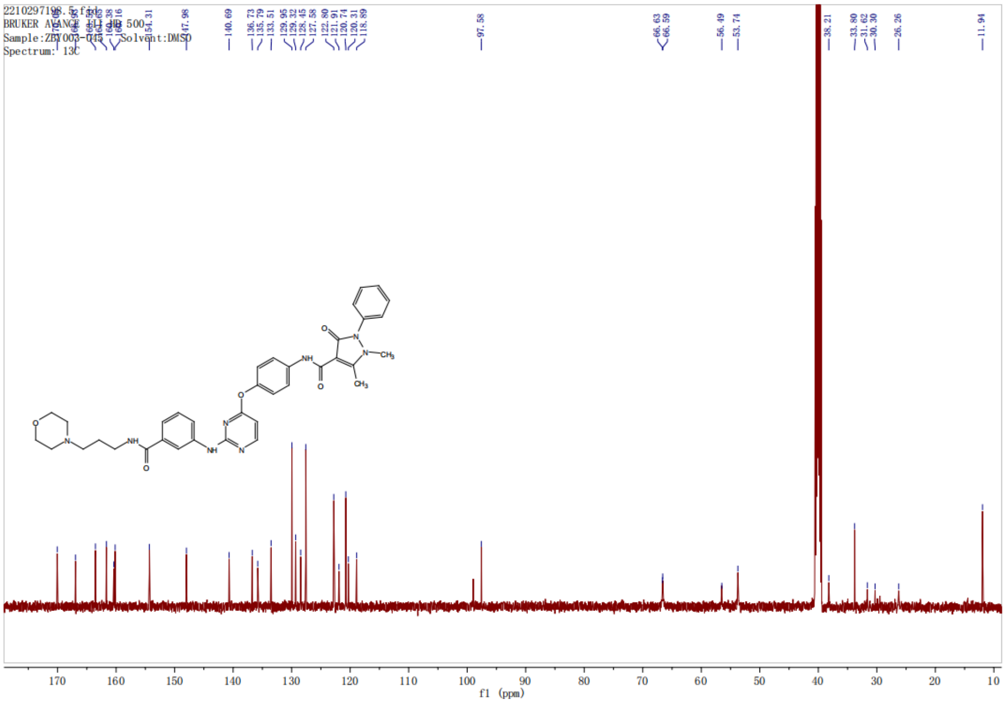

S15: ^{13}C-NMR of compound **14e**

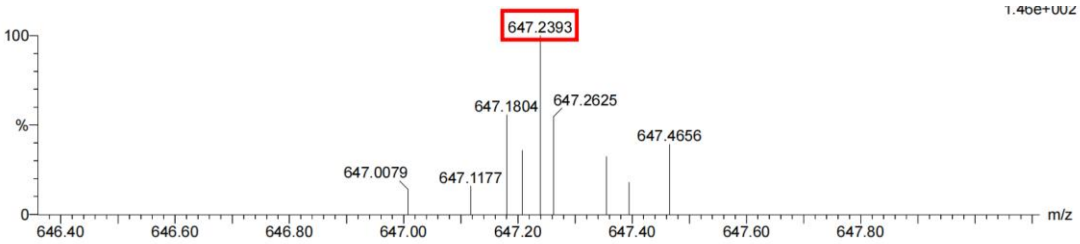

S16: HRMS of compound **14f**

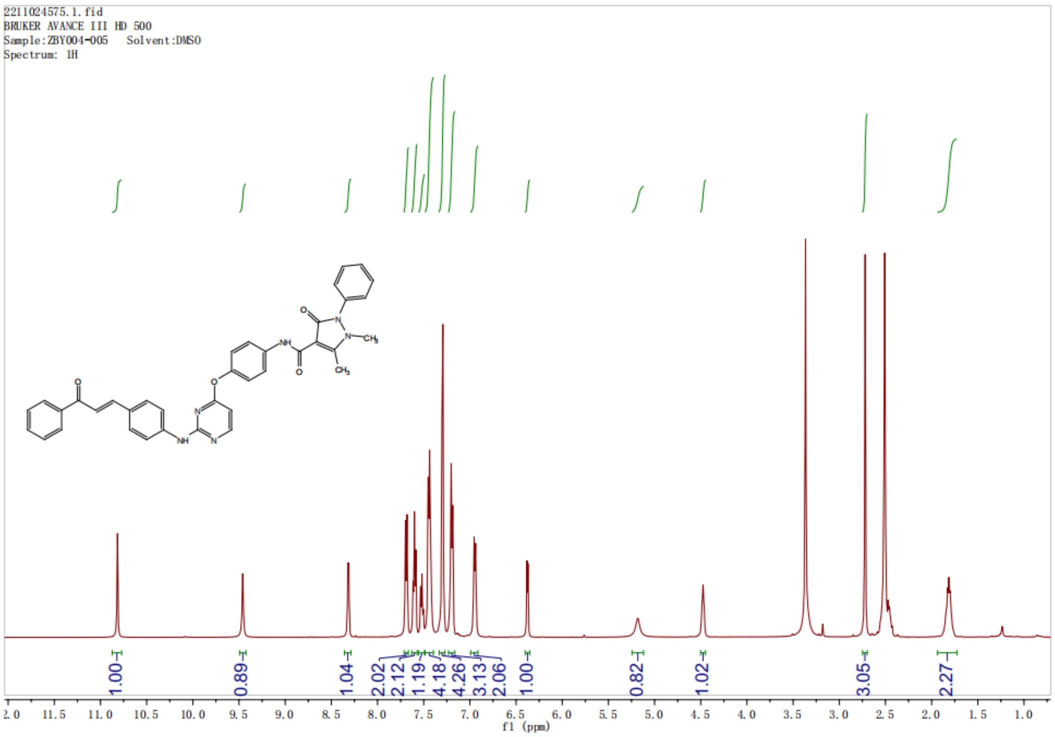

S17: ^{1}H-NMR of compound **14f**

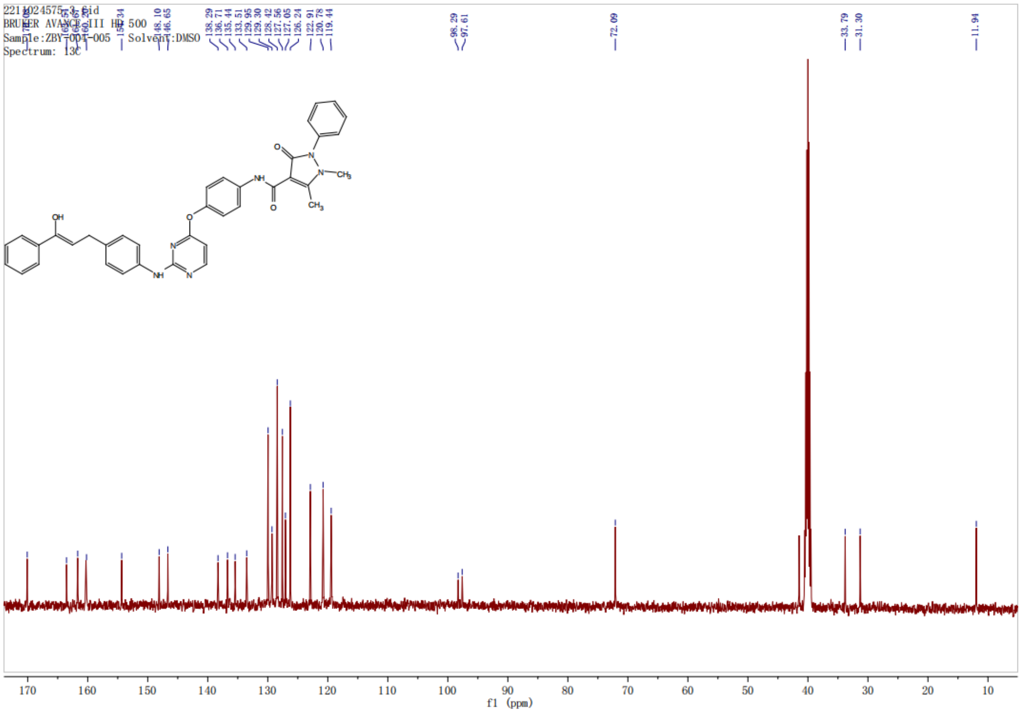

S18: ^{13}C-NMR of compound **14f**

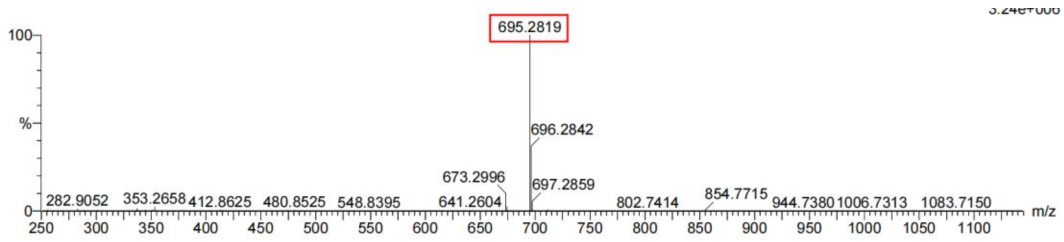

S19: HRMS of compound **14g**

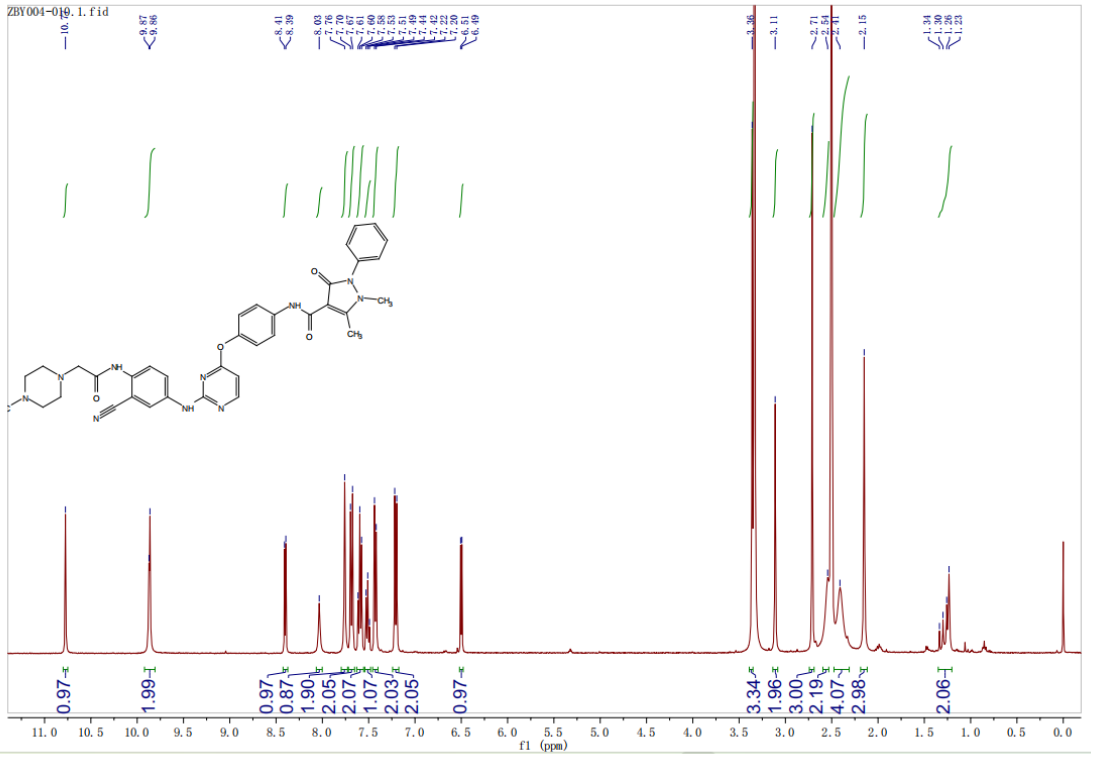

S20: ^{1}H-NMR of compound **14g**

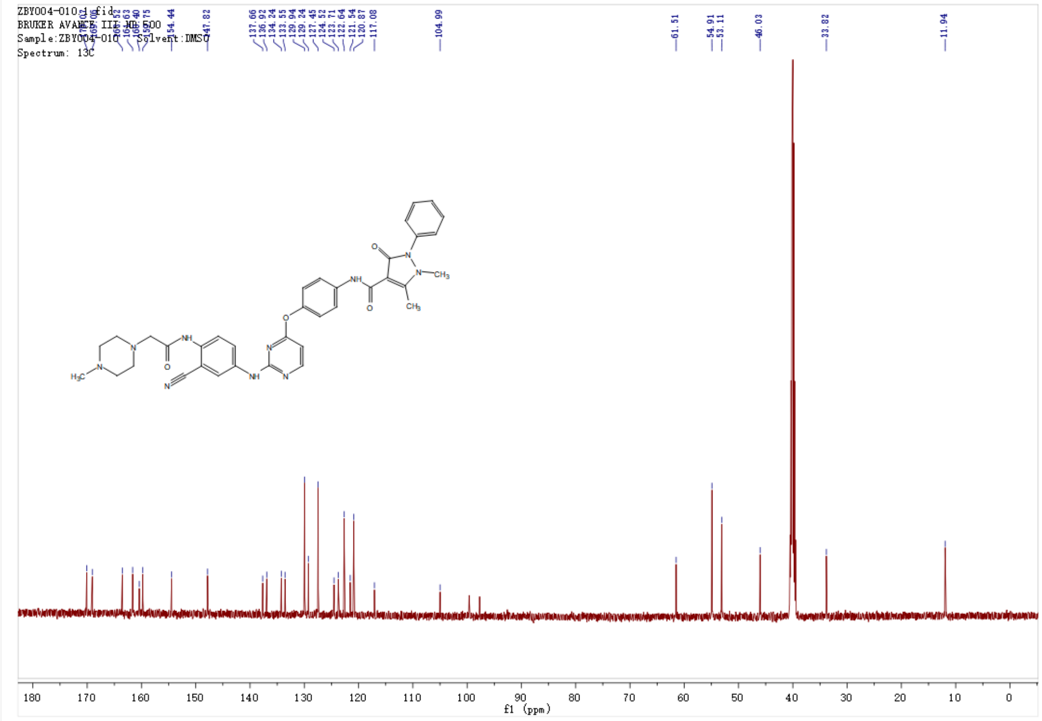

S$_{21}$: ^{13}C-NMR of compound **14g**

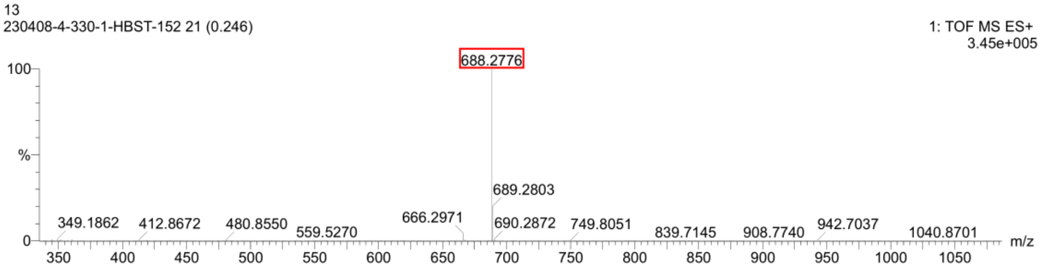

S$_{22}$: HRMS of compound **14h**

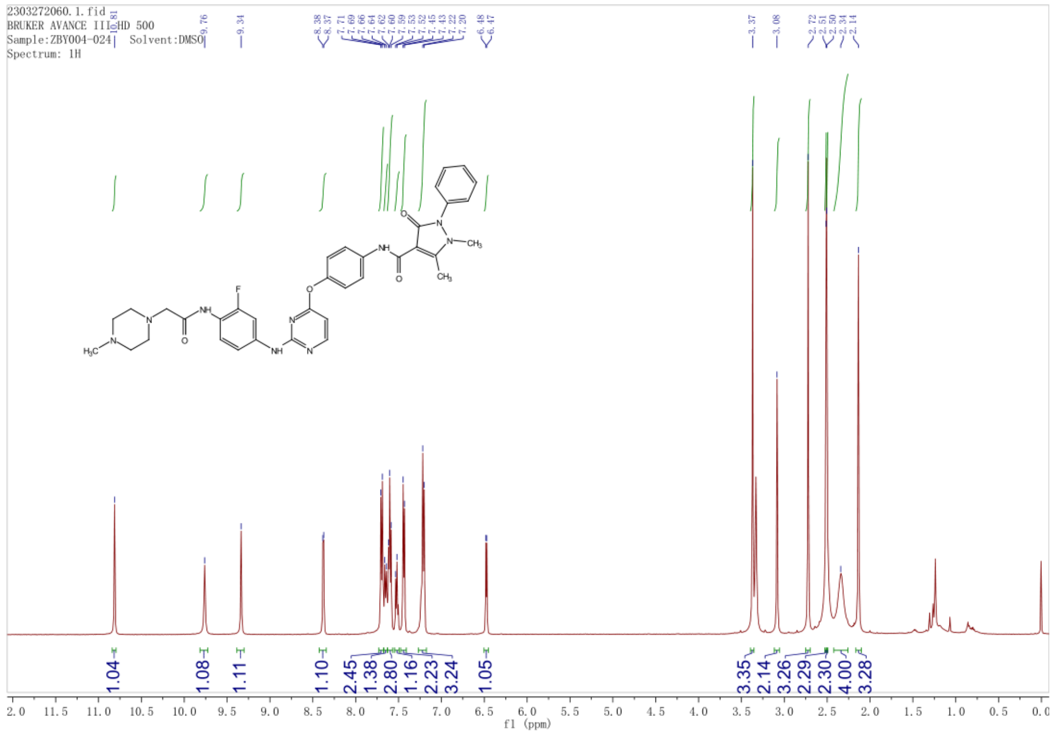

S$_{23}$: ^{1}H-NMR of compound **14h**

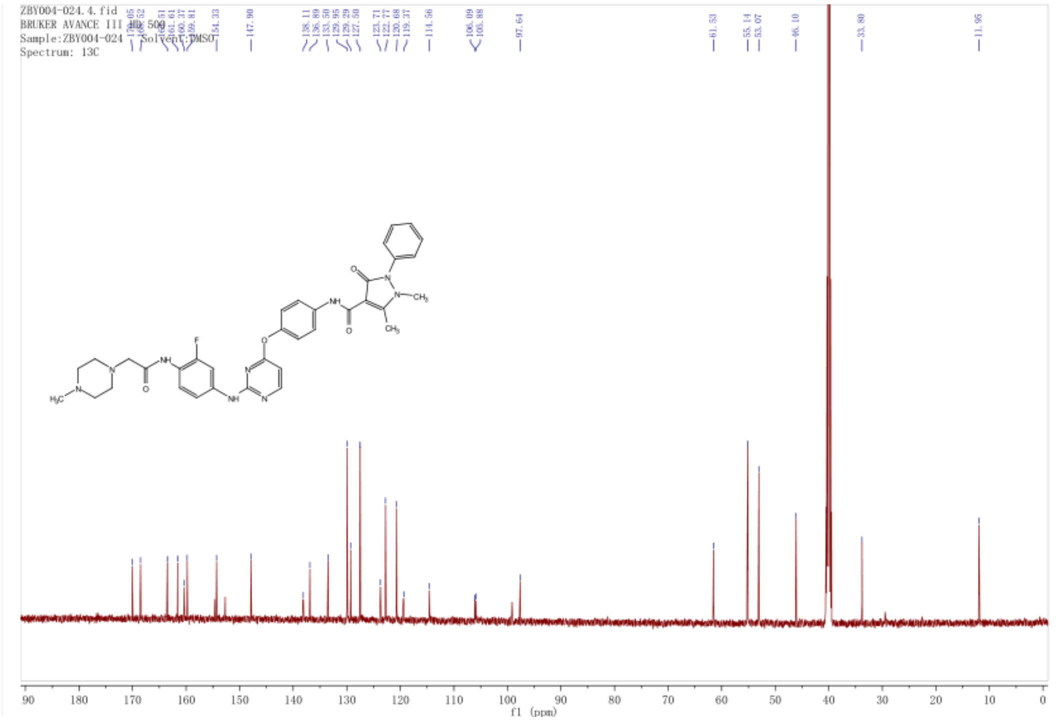

S$_{24}$: ^{13}C-NMR of compound **14h**

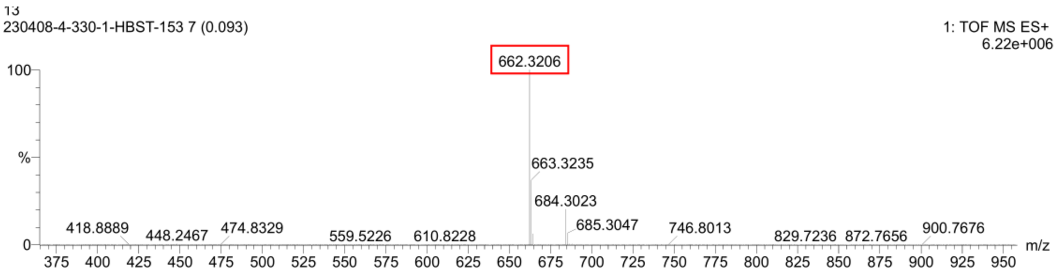

S$_{25}$: HRMS of compound **14i**

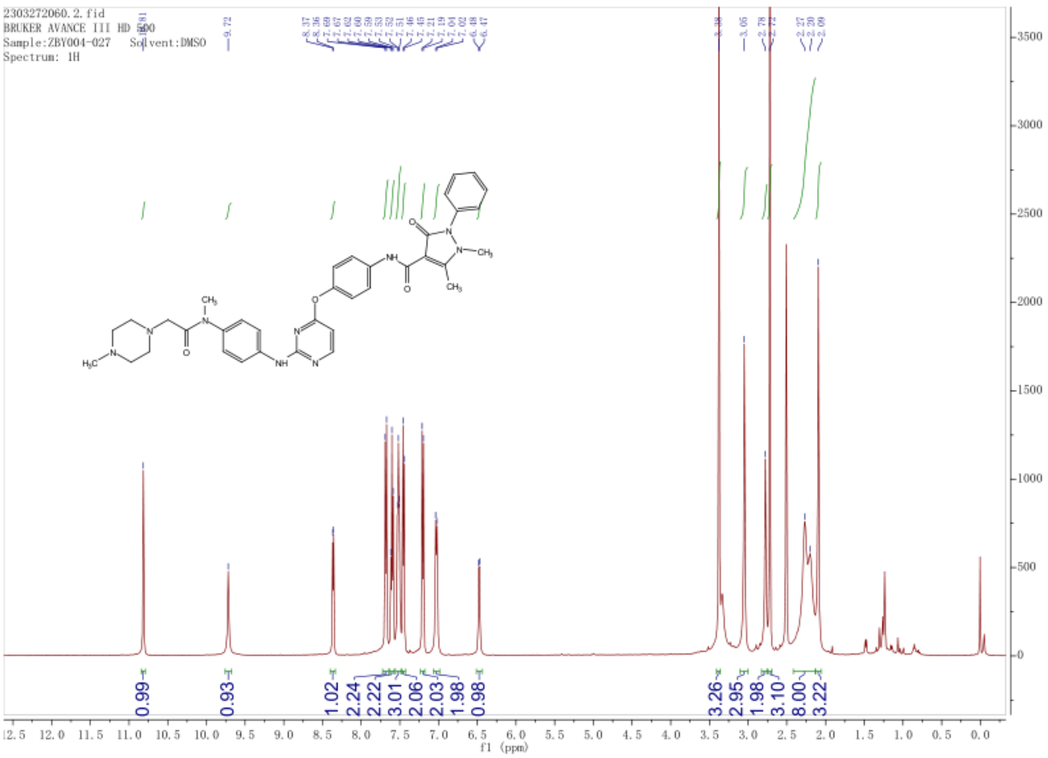

S$_{26}$: ^{1}H-NMR of compound **14i**

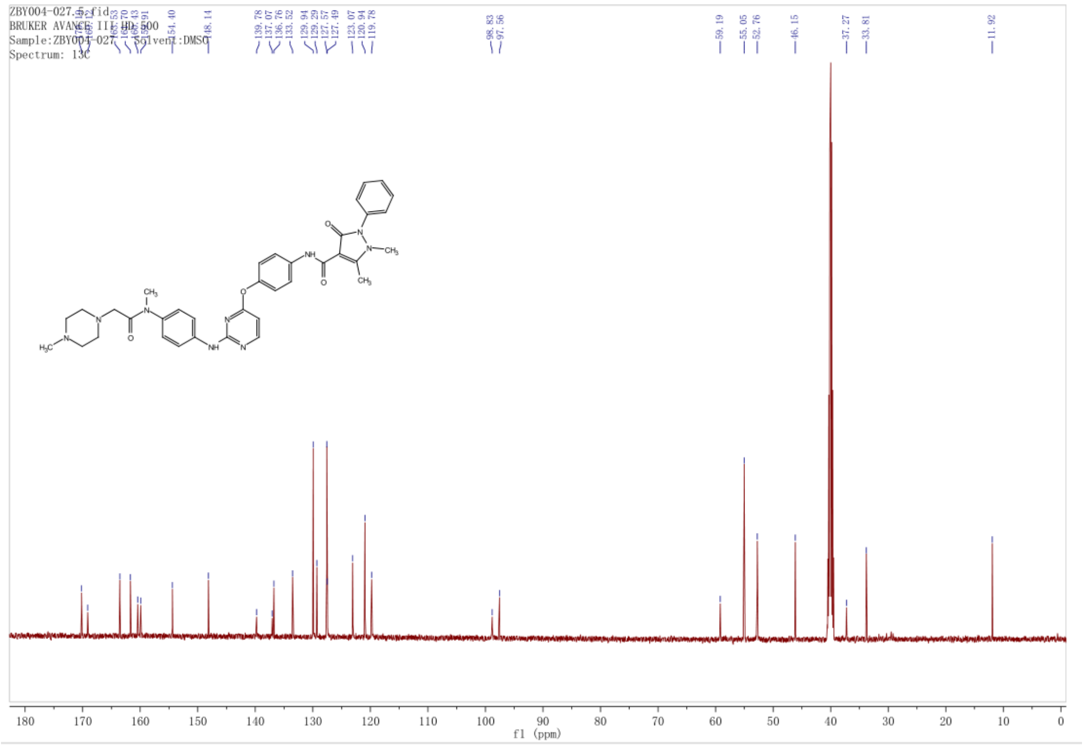

S$_{27}$: ^{13}C-NMR of compound **14i**

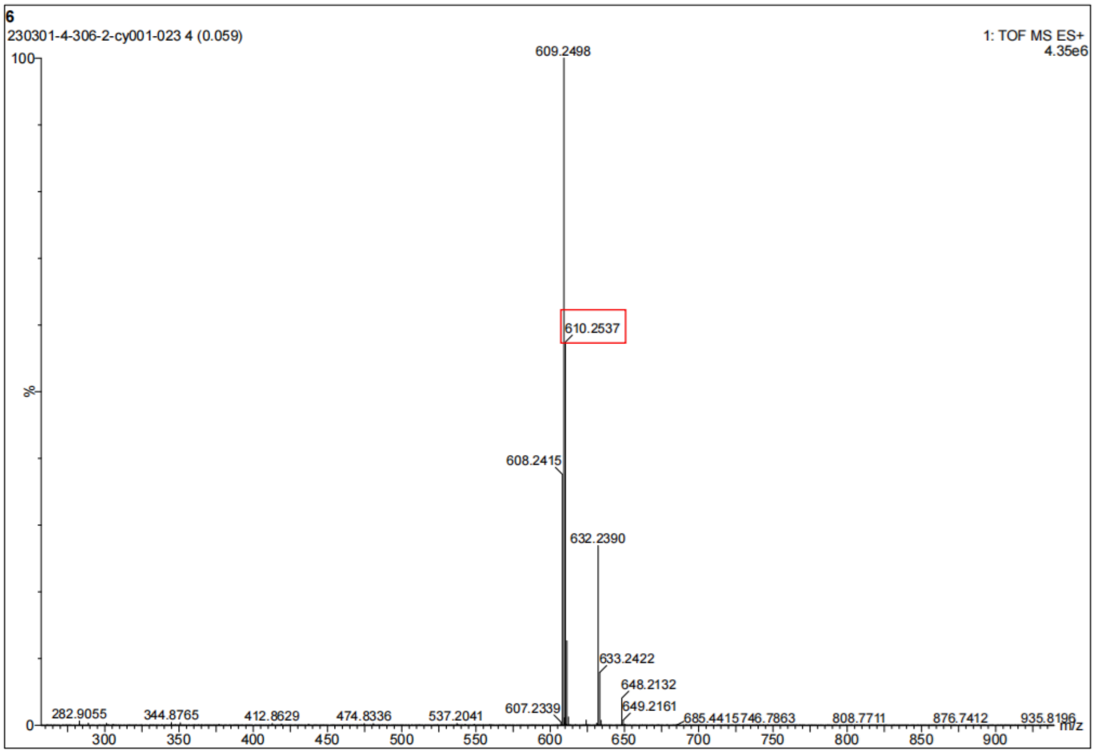

S$_{28}$: HRMS of compound **18a**

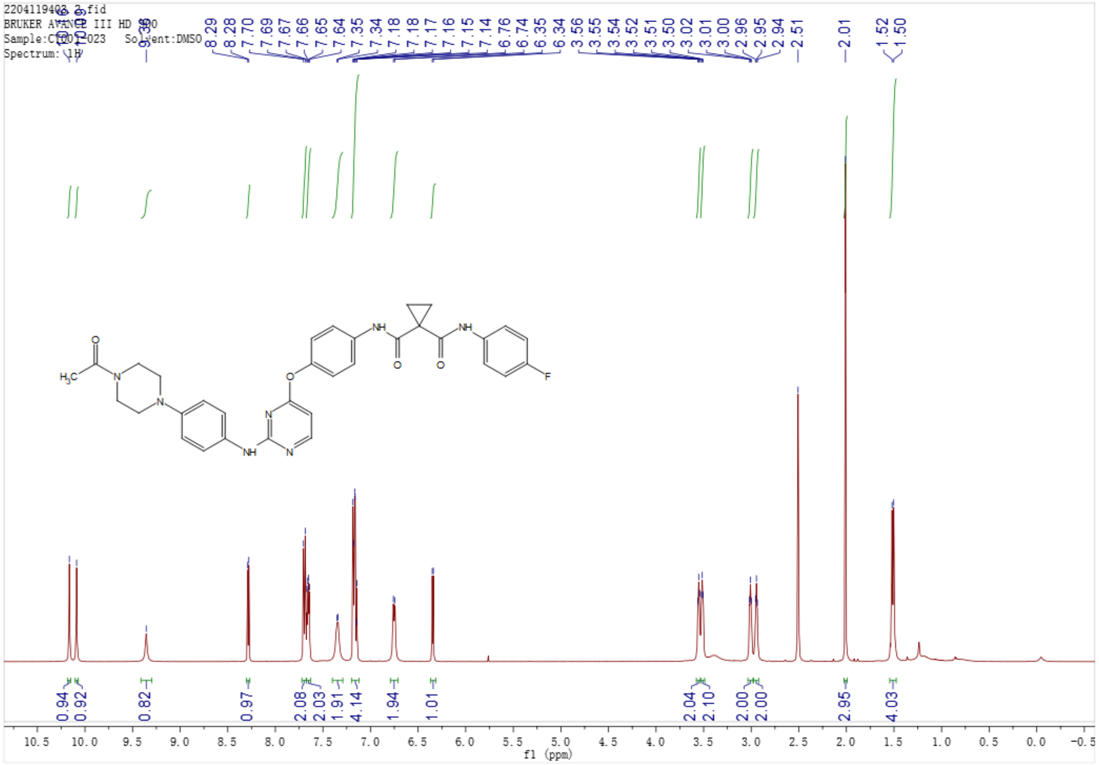

S$_{29}$: ^{1}H-NMR of compound **18a**

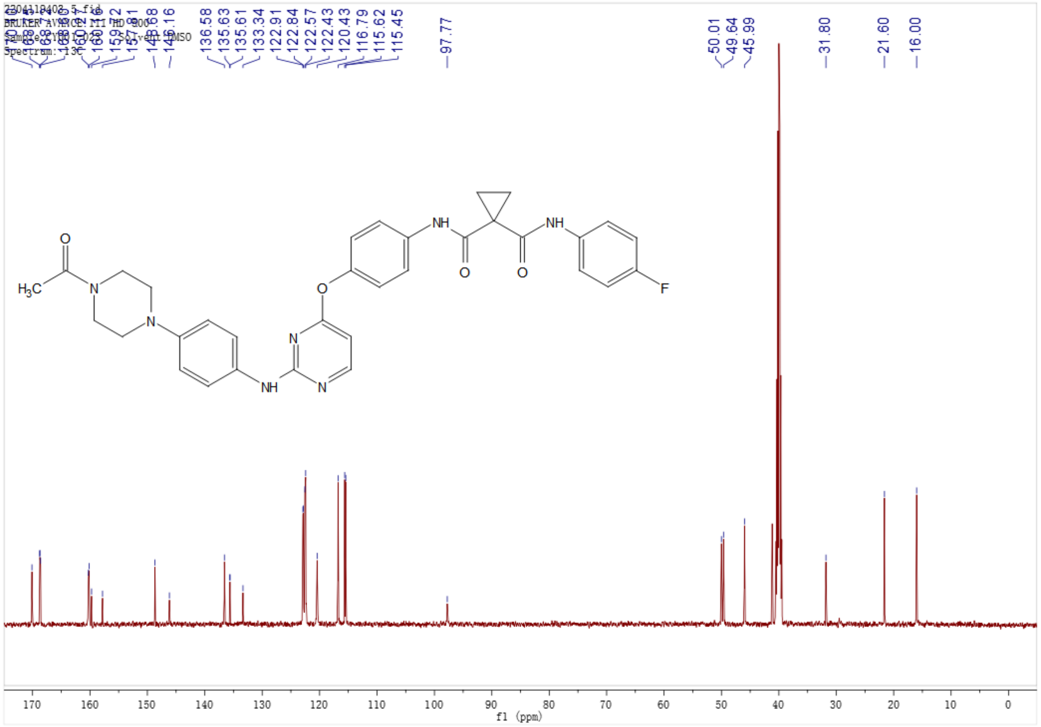

S$_{30}$: ^{13}C-NMR of compound **18a**

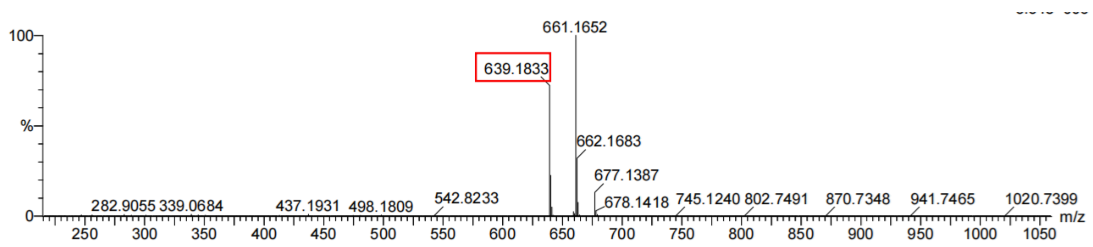

S$_{31}$: HRMS of compound **18b**

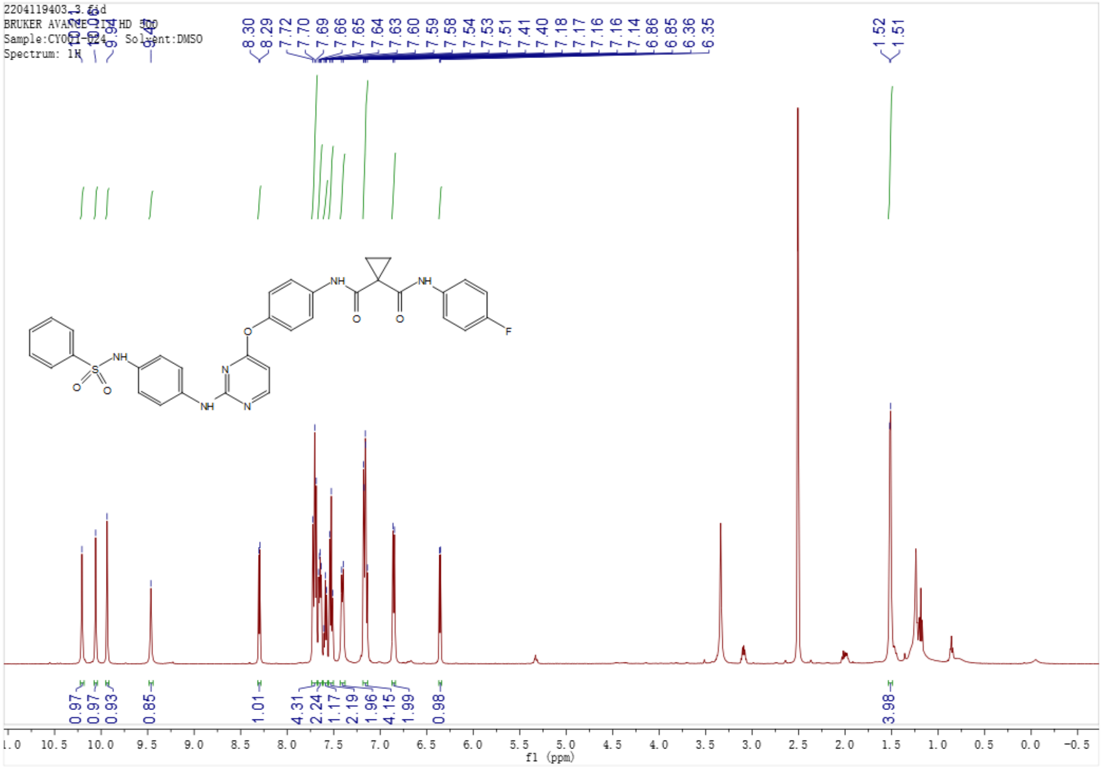

S$_{32}$: ^{1}H-NMR of compound **18b**

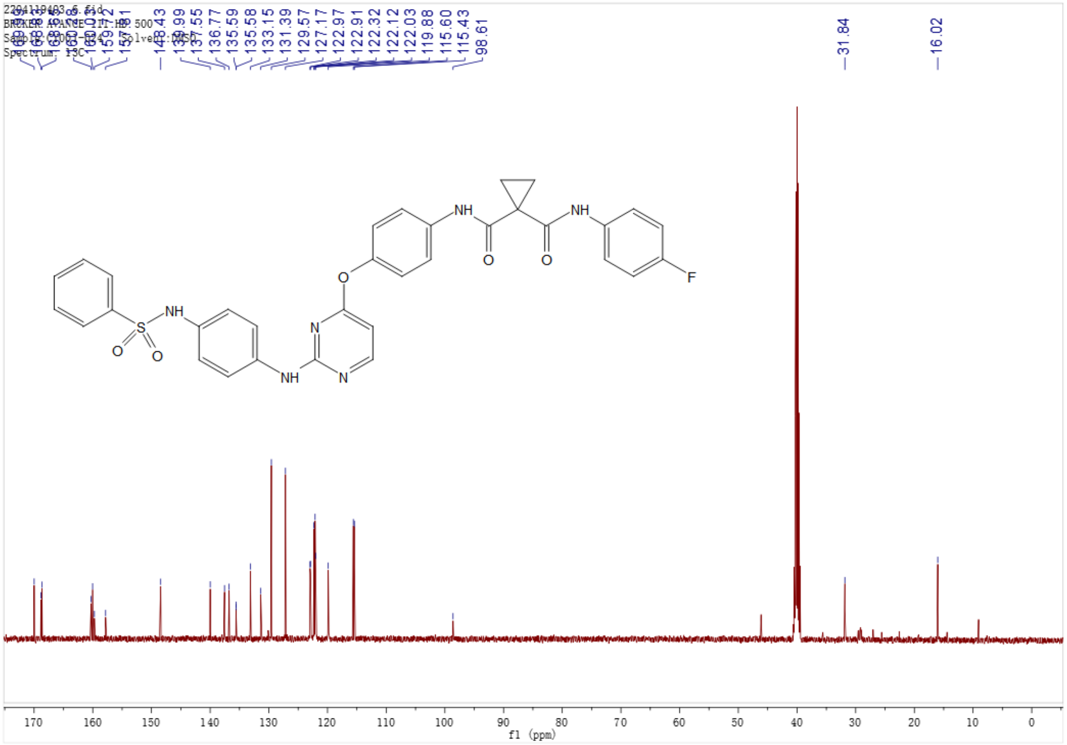

S33: ^{13}C-NMR of compound **18b**

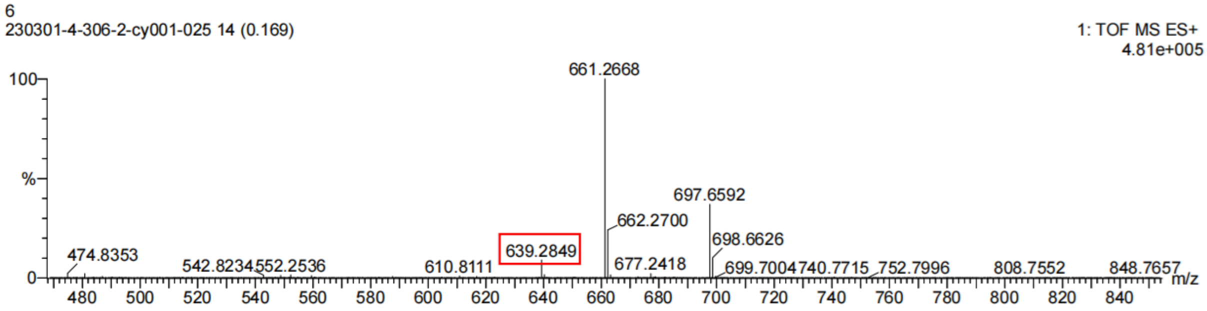

S34: HRMS of compound **18c**

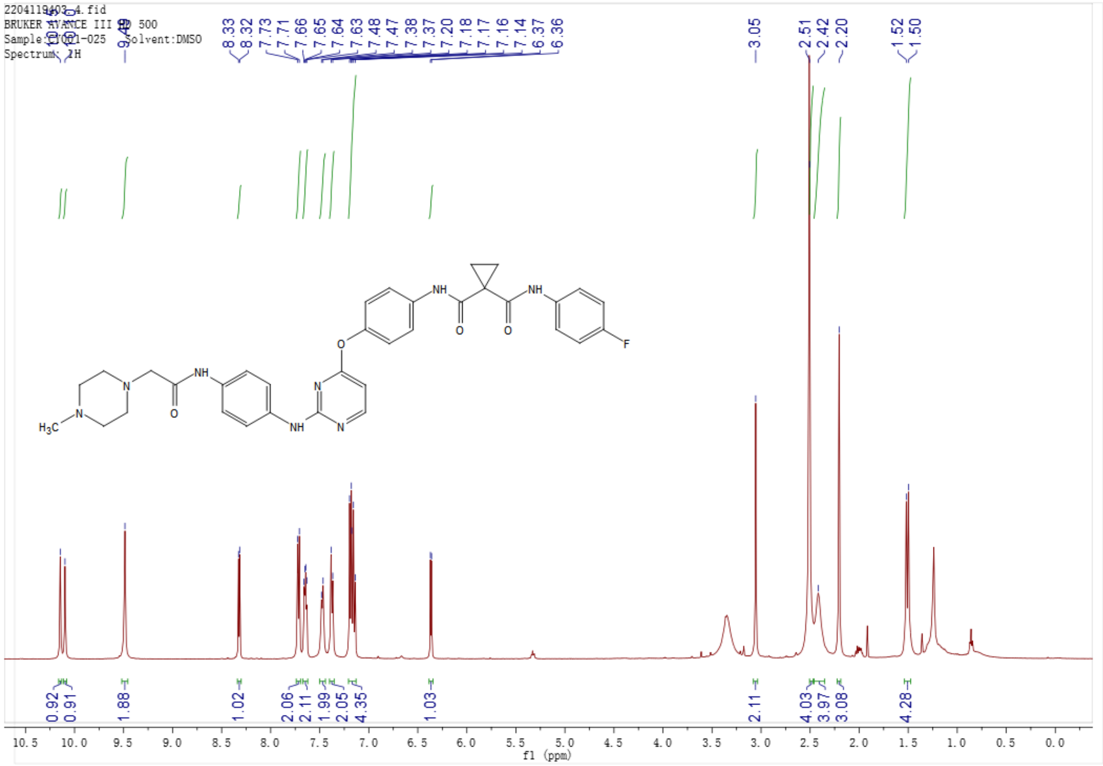

S35: ^{1}H-NMR of compound **18c**

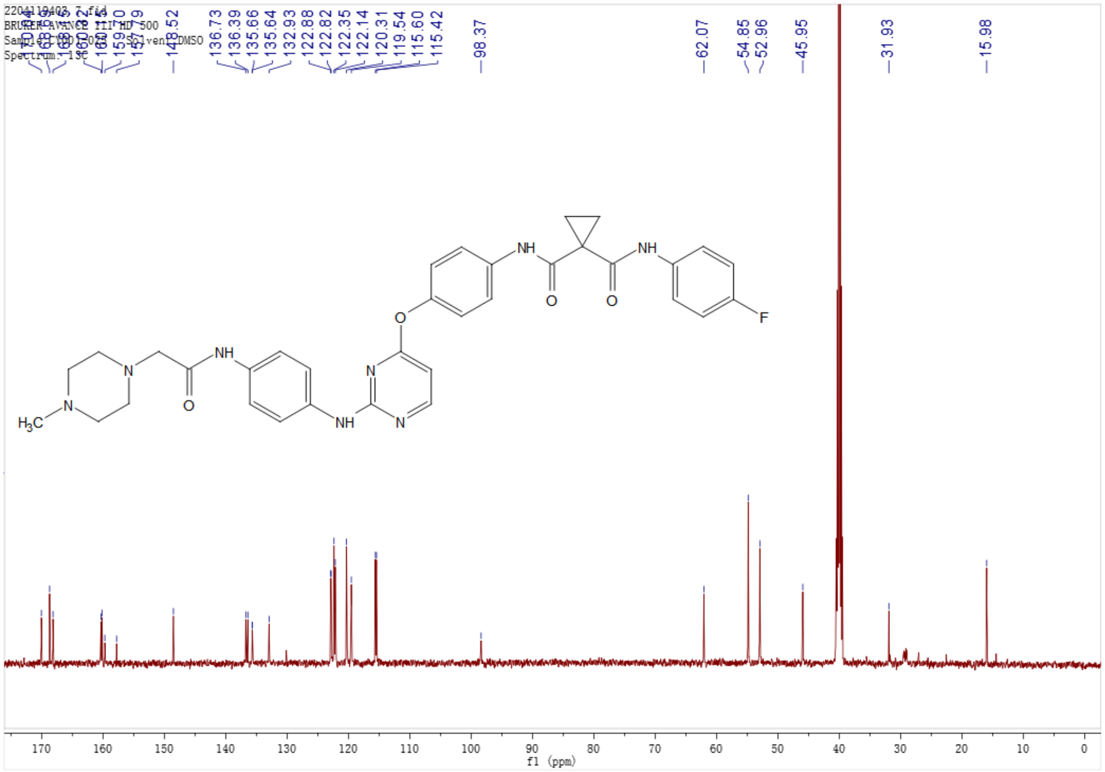

S36: ^{13}C-NMR of compound **18c**

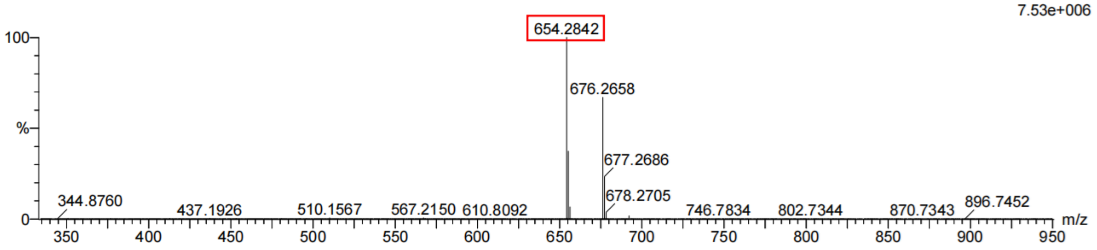

S$_{37}$: HRMS of compound **18d**

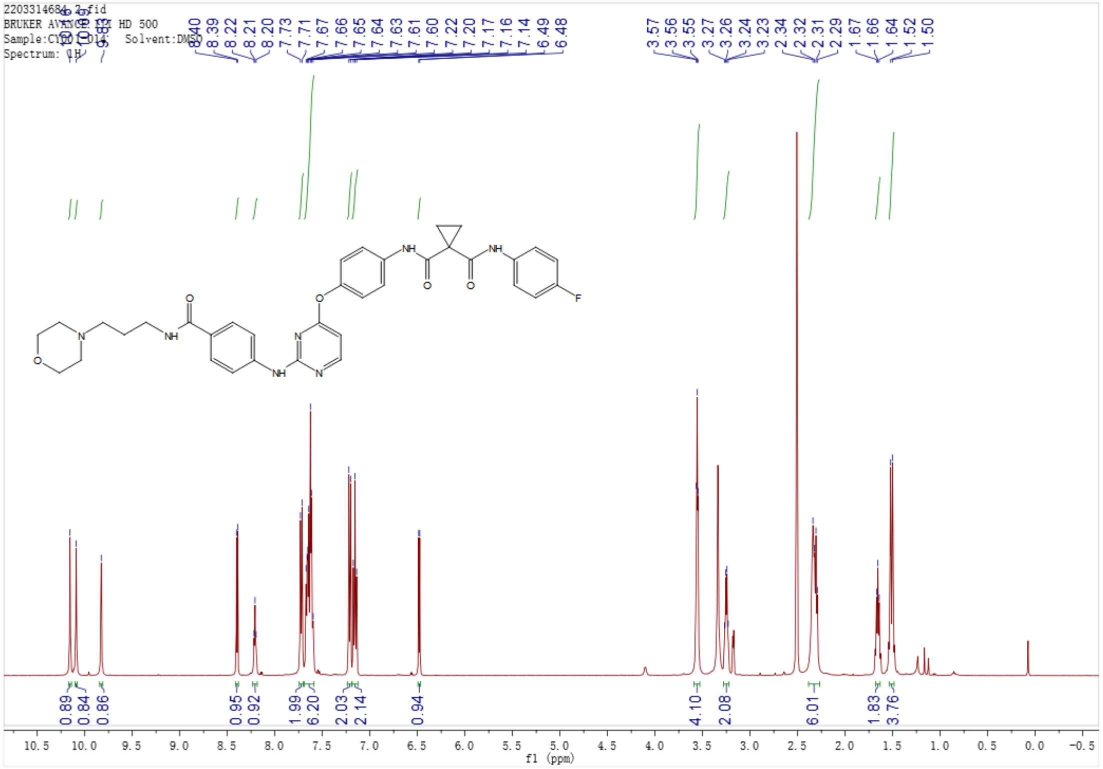

S$_{38}$: ^{1}H-NMR of compound **18d**

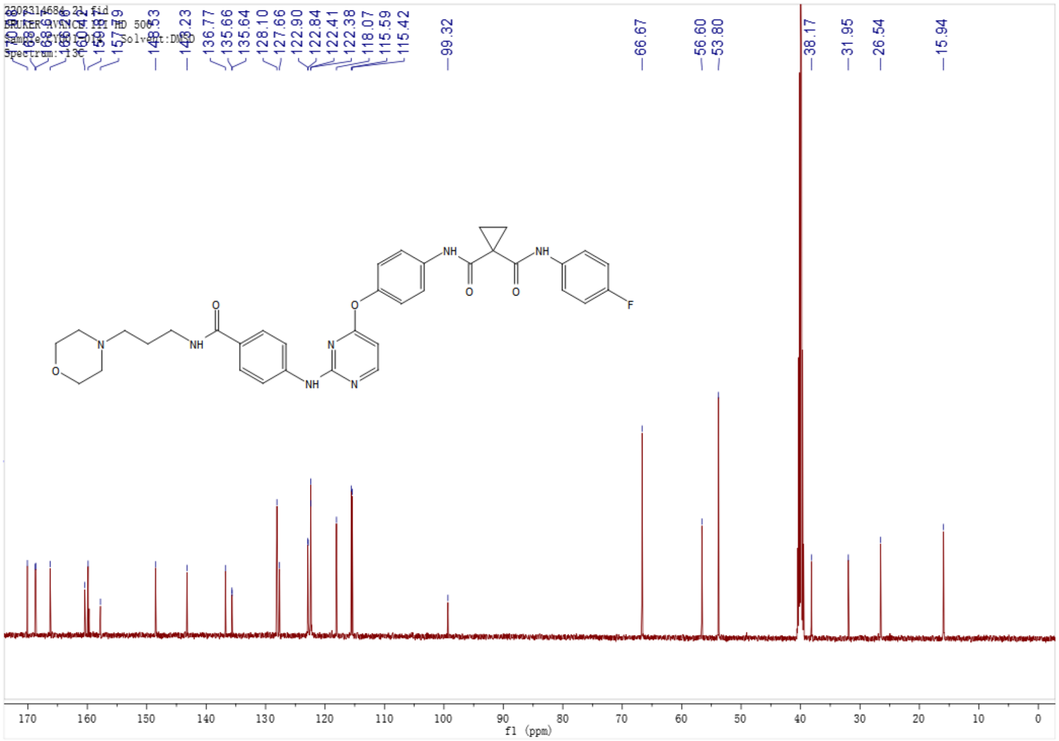

S39: ^{13}C-NMR of compound **18d**

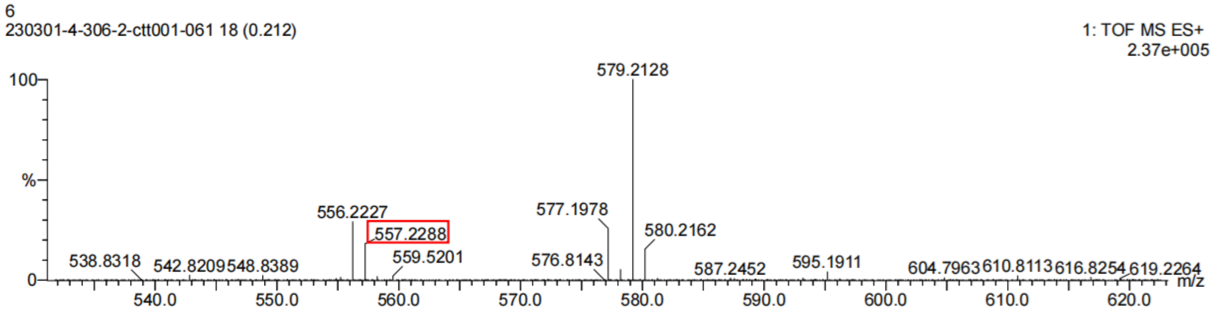

S40: HRMS of compound **18e**

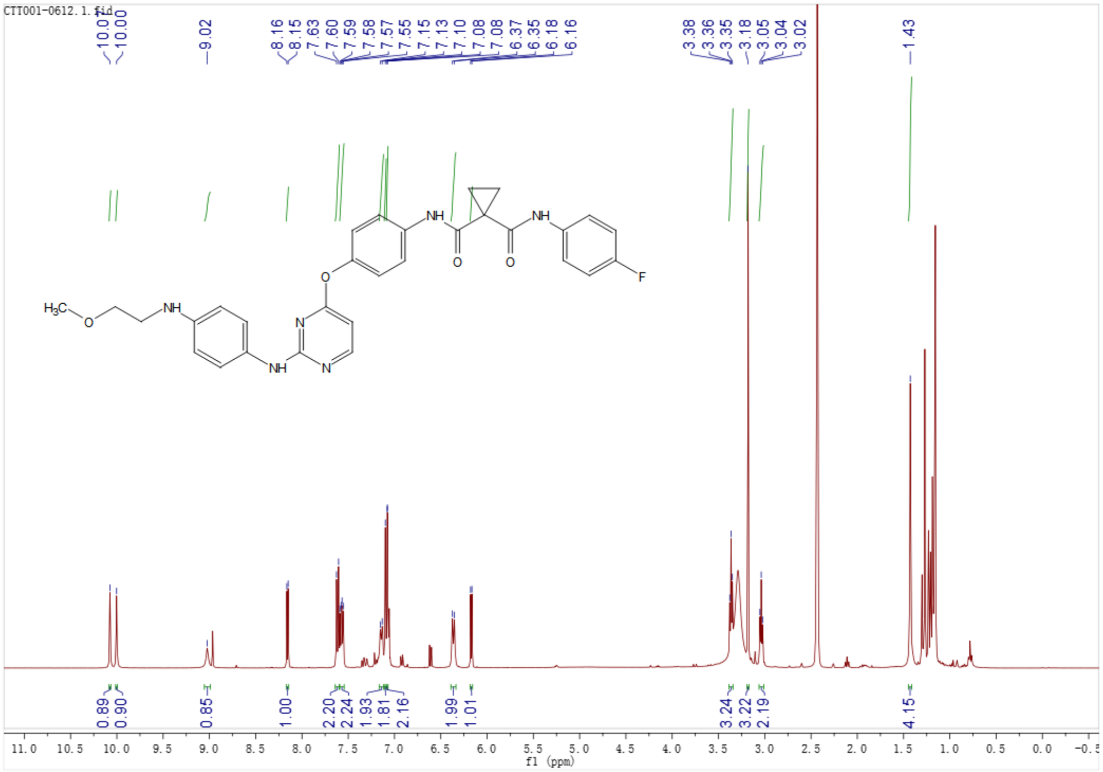

S41: ^{1}H-NMR of compound **18e**

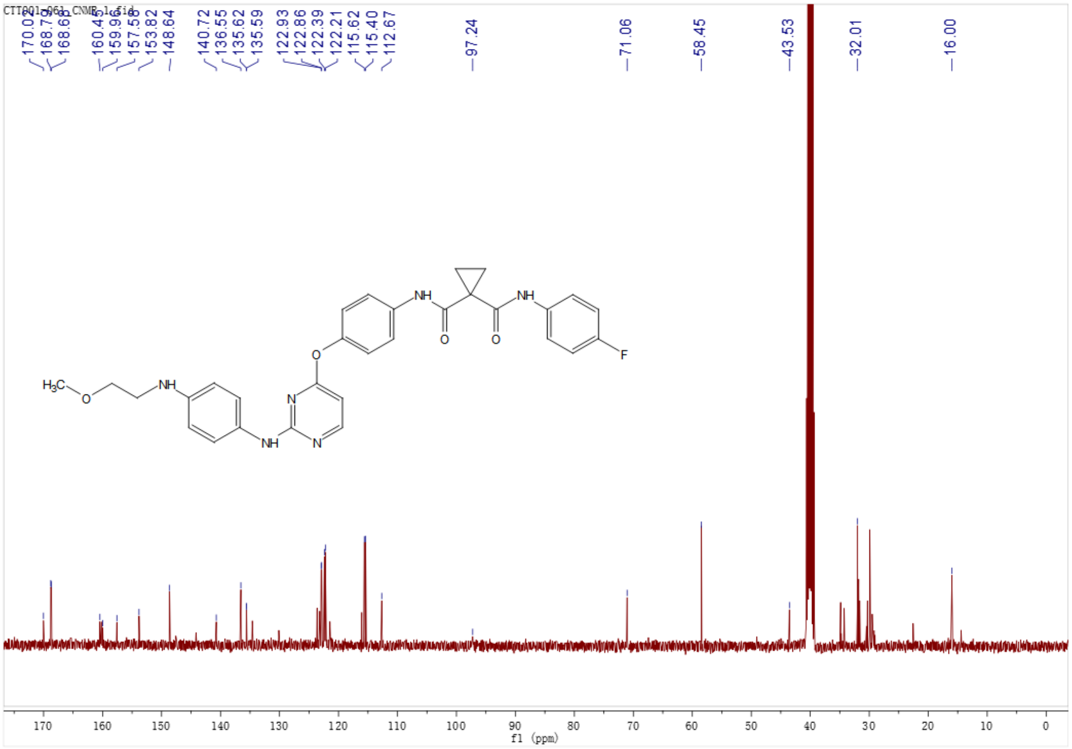

S42: ^{13}C-NMR of compound **18e**

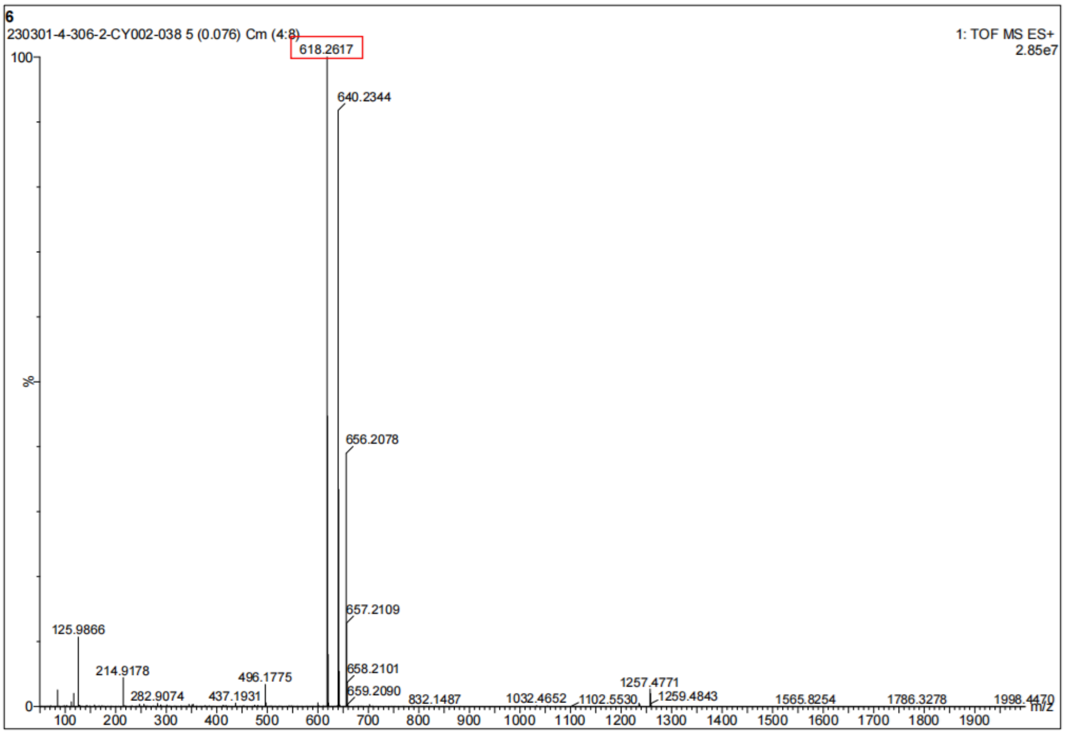

S43: HRMS of compound **18f**

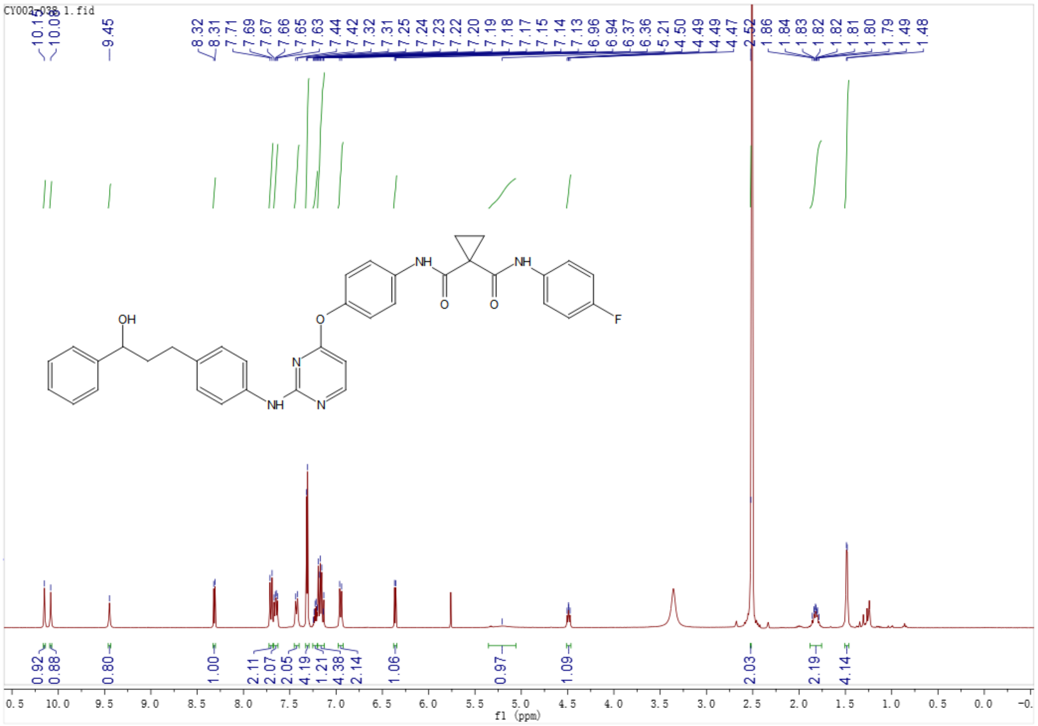

S44: ^{1}H-NMR of compound **18f**

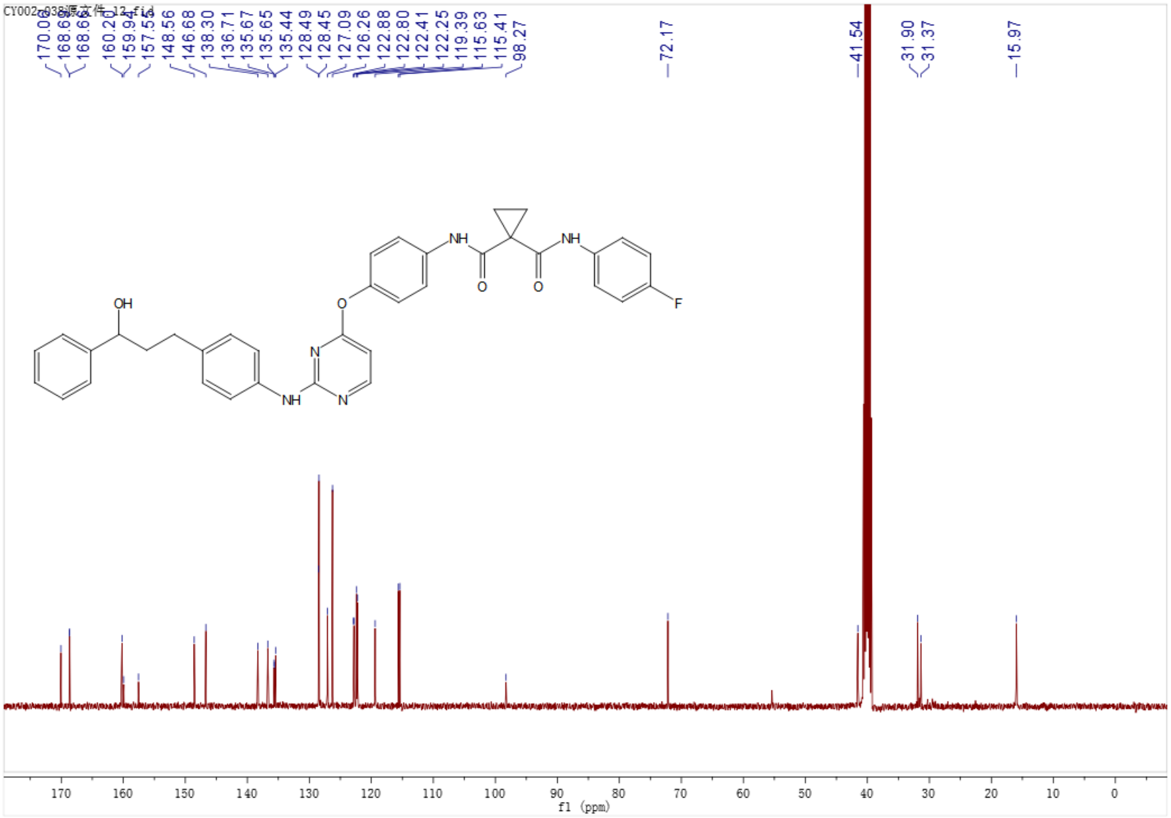

S45: ^{13}C-NMR of compound **18f**

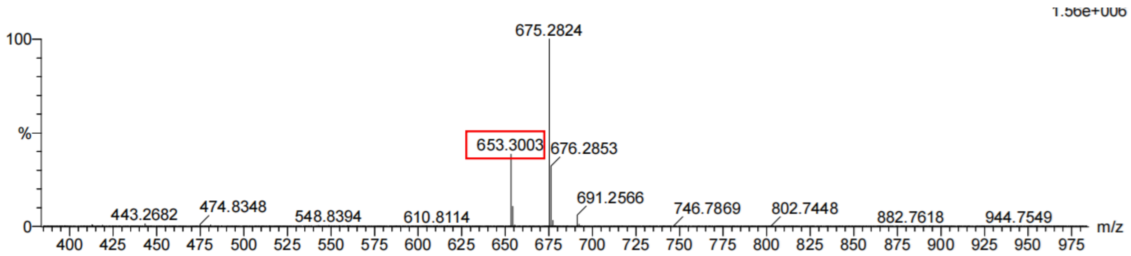

S46: HRMS of compound **18g**

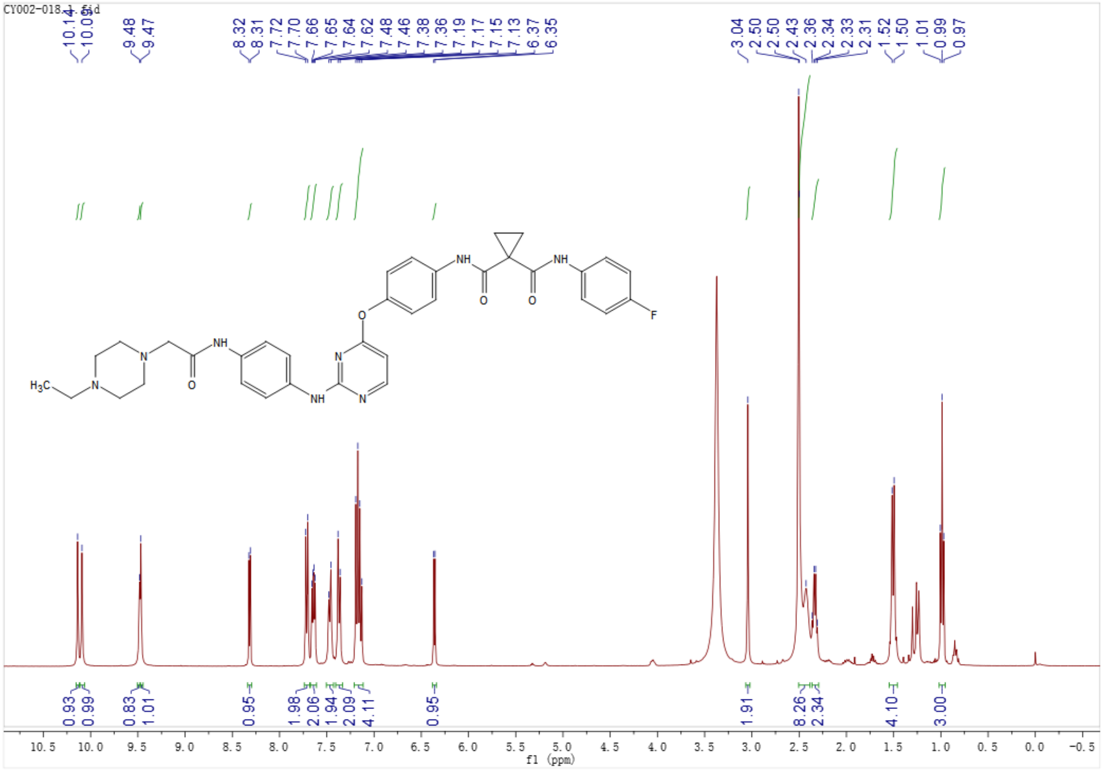

S47: ^{1}H-NMR of compound **18g**

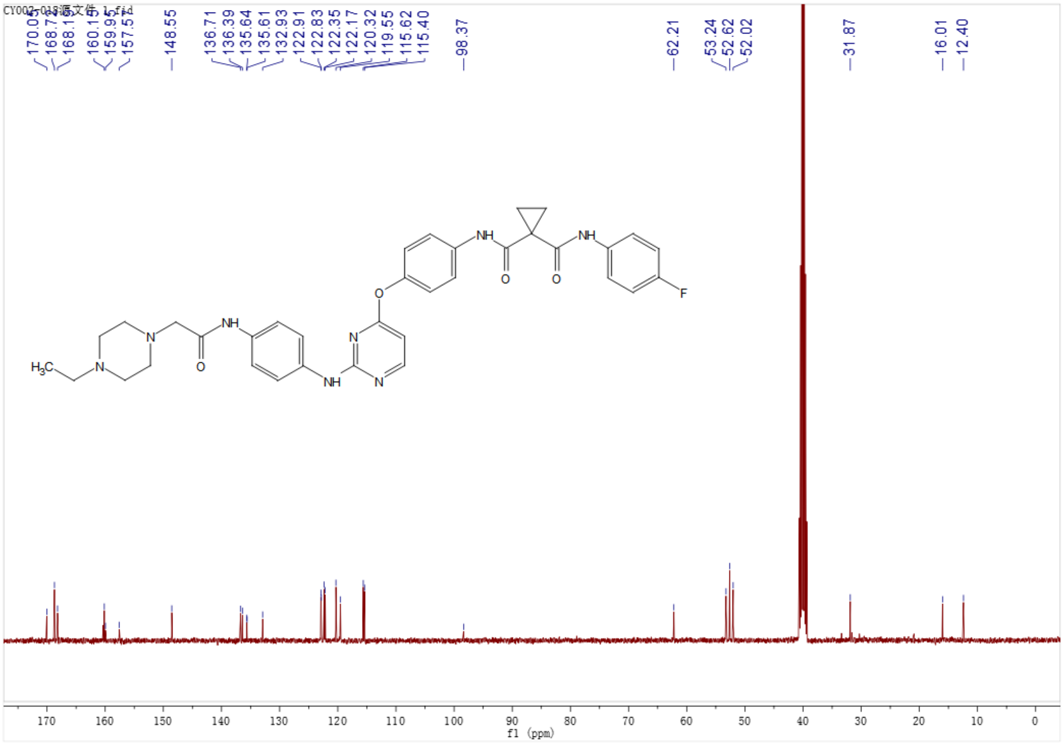

S48: ^{13}C-NMR of compound **18g**

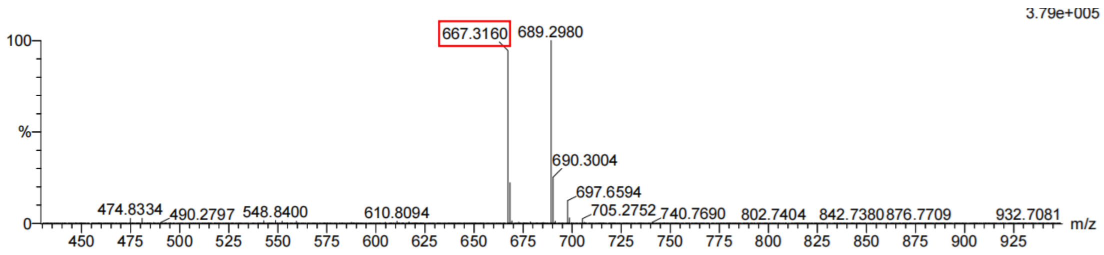

S49: HRMS of compound **18h**

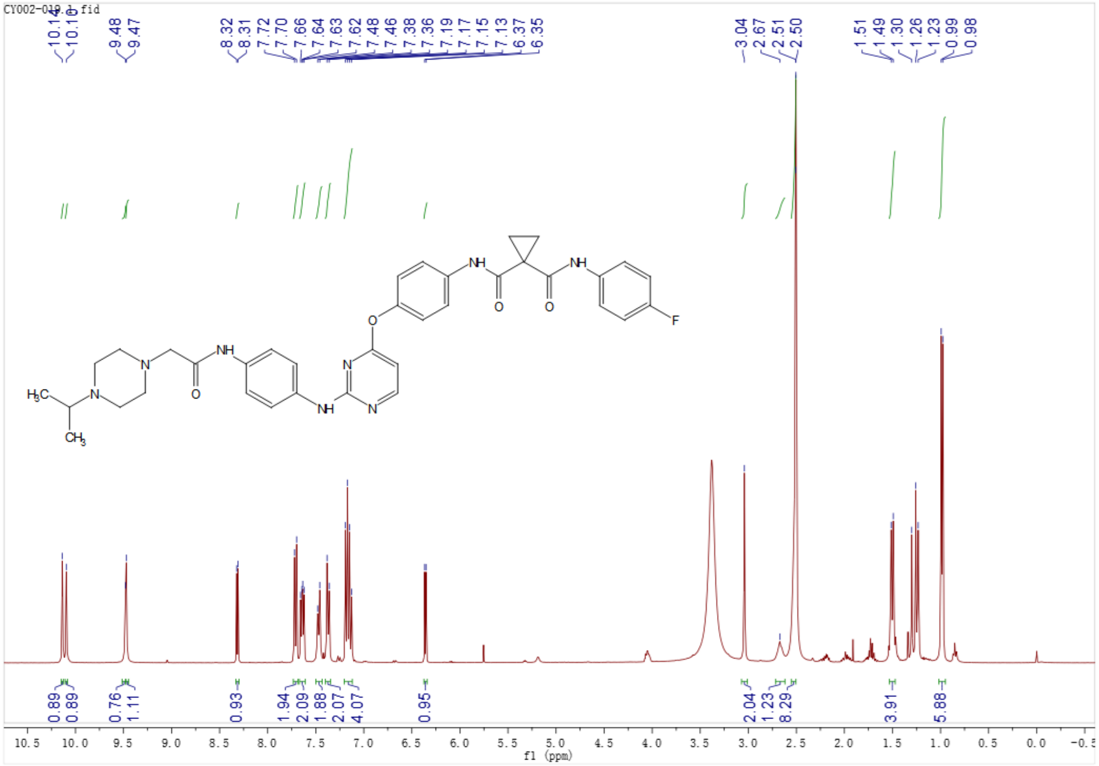

S50: ^{1}H-NMR of compound **18h**

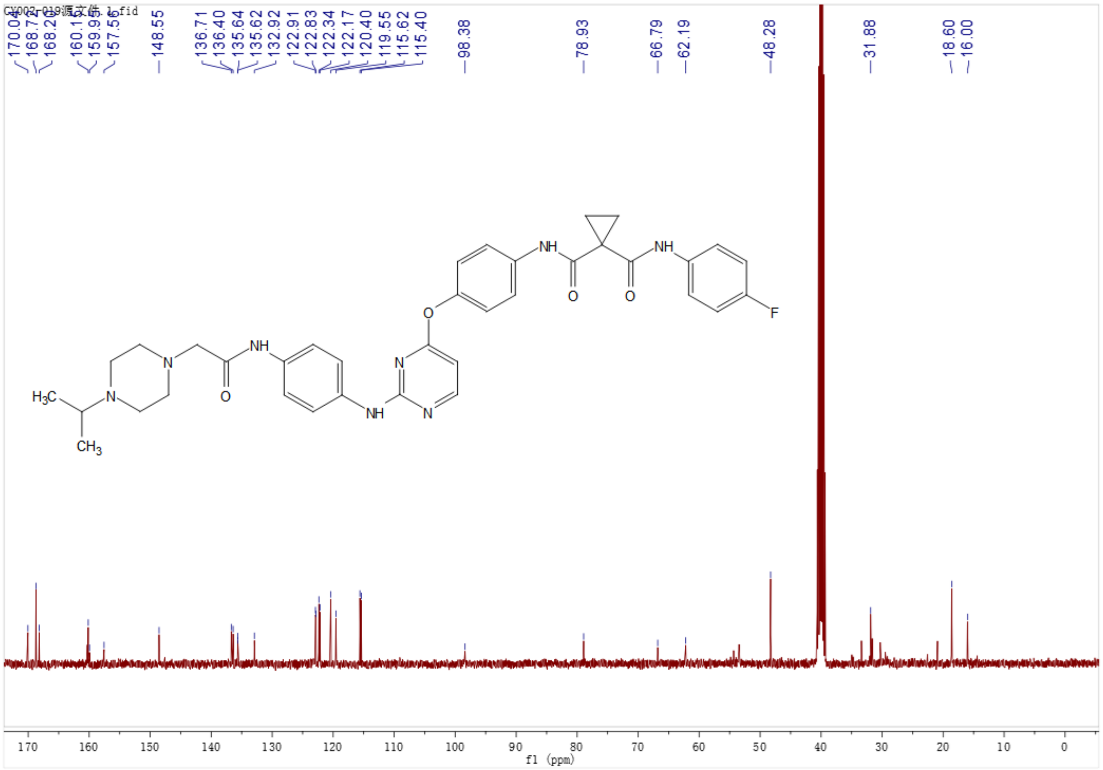

S51: ^{13}C-NMR of compound **18h**

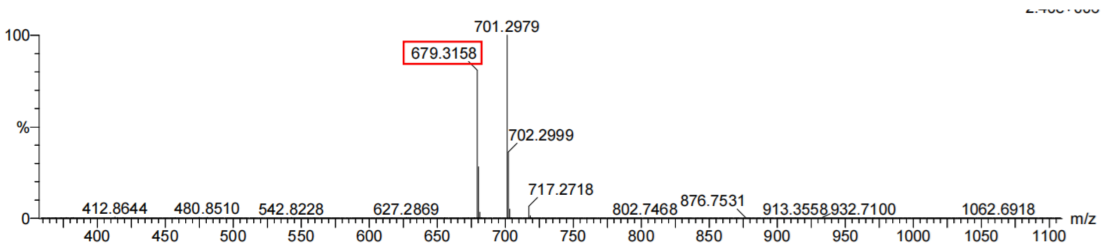

S52: HRMS of compound **18i**

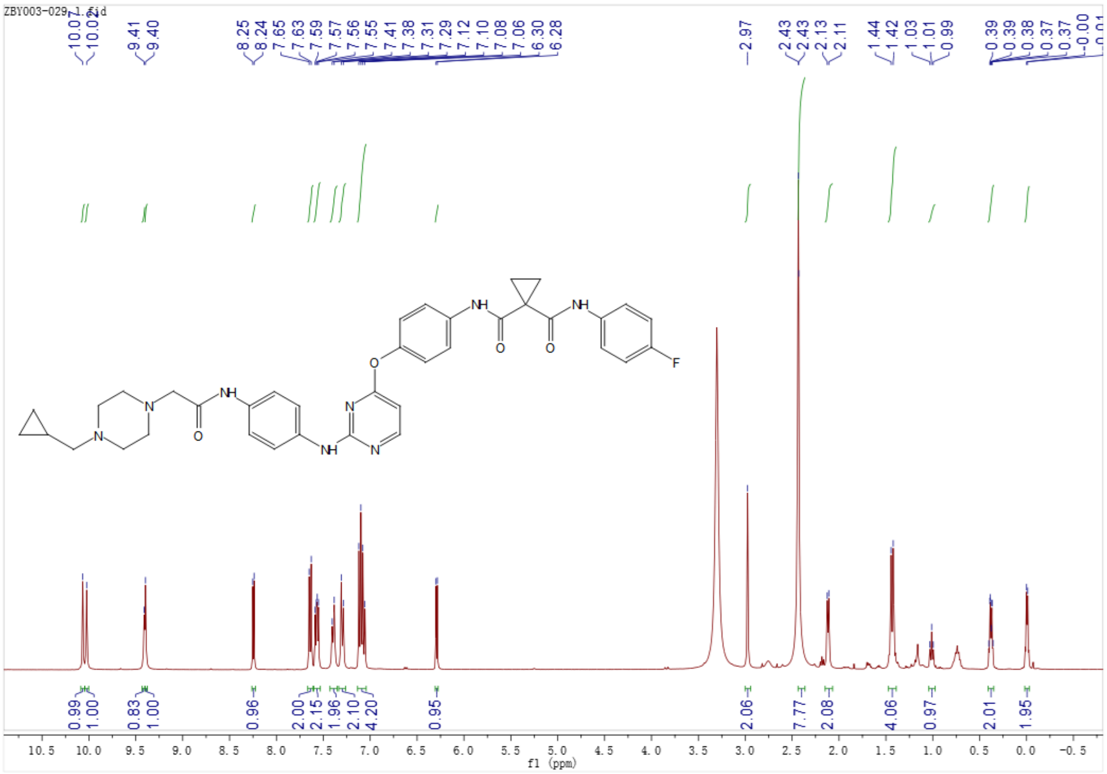

S$_{53}$: ^{1}H-NMR of compound **18i**

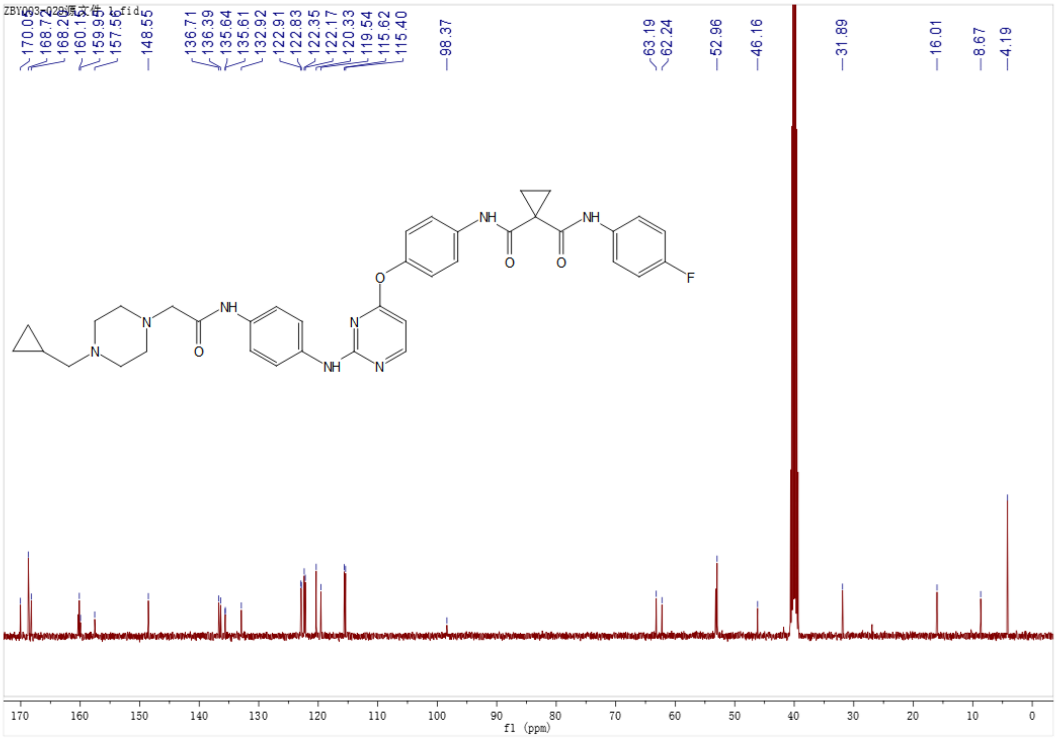

S$_{54}$: ^{13}C-NMR of compound **18i**

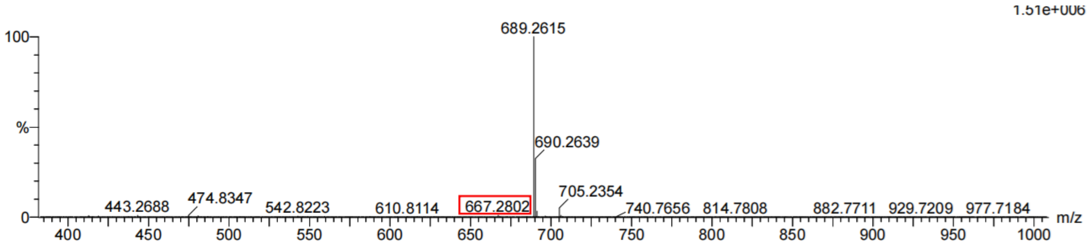

S$_{55}$: HRMS of compound **18j**

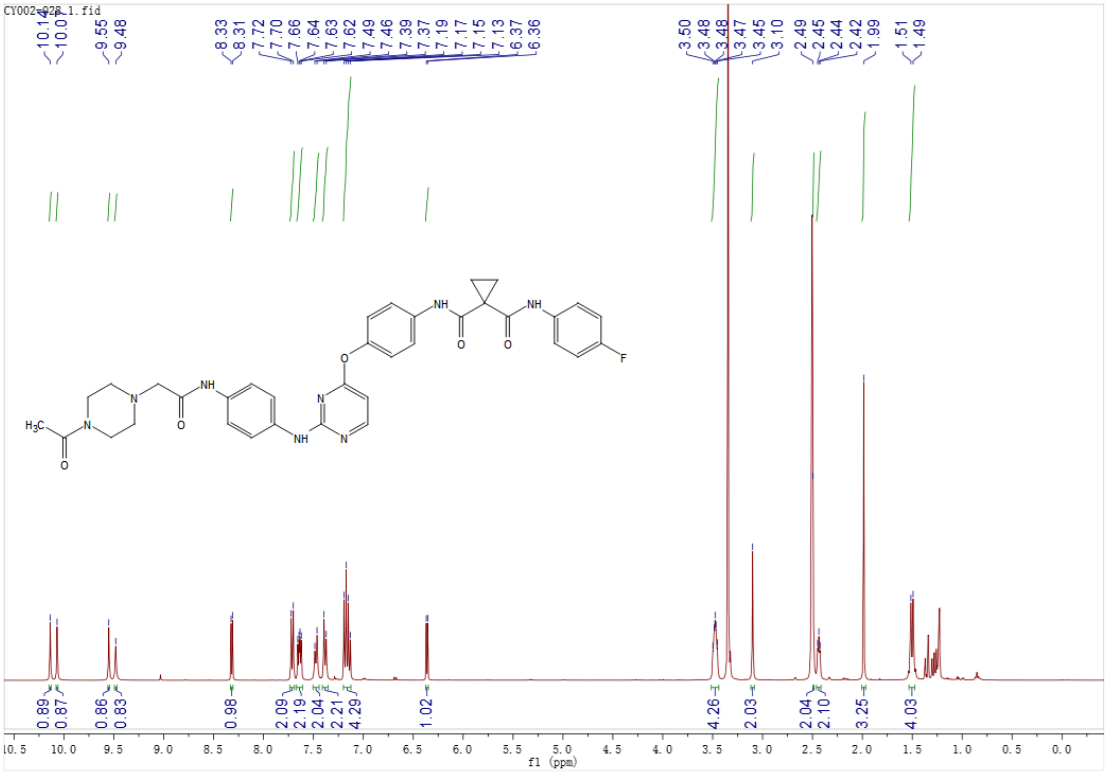

S$_{56}$: ^{1}H-NMR of compound **18j**

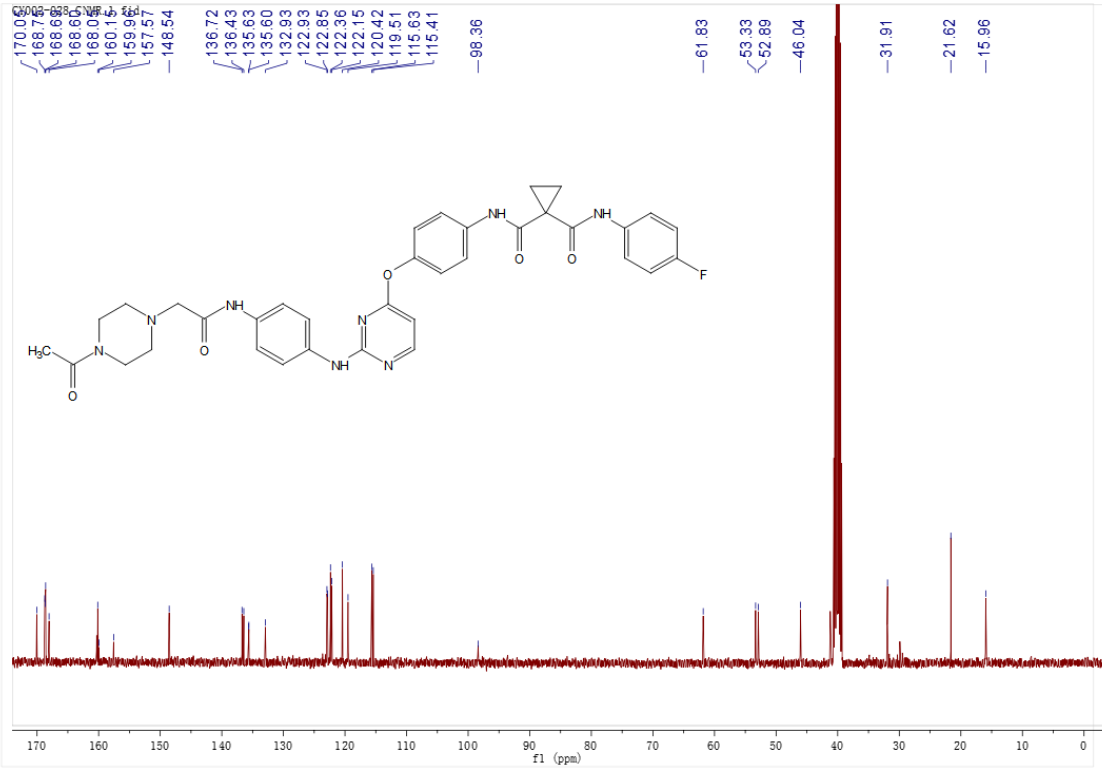

S57: ^{13}C-NMR of compound **18j**

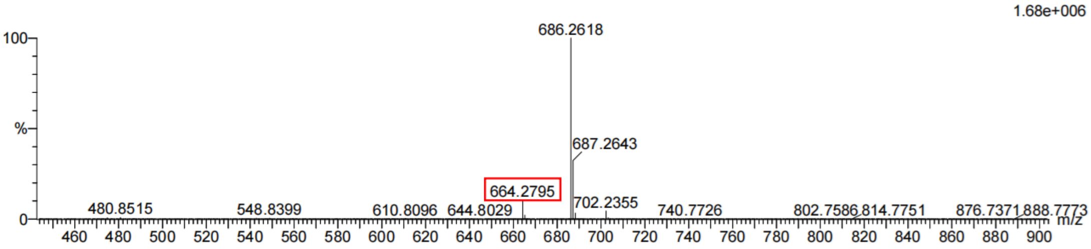

S58: HRMS of compound **18k**

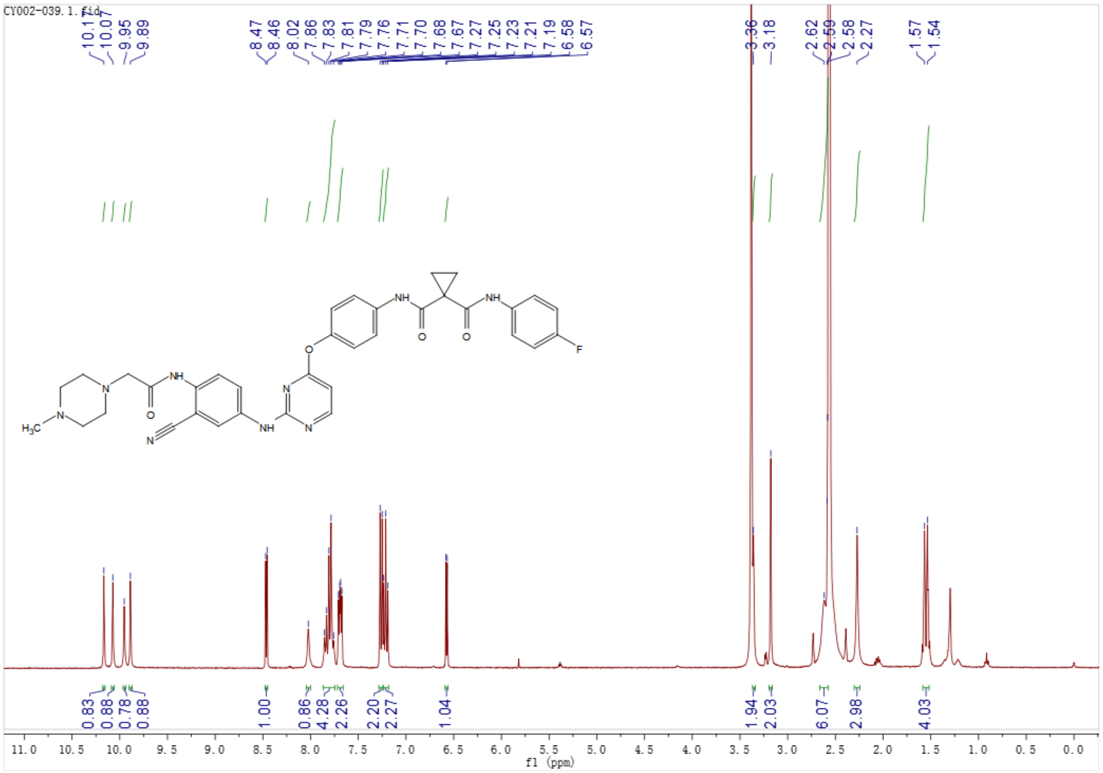

S59: ^{1}H-NMR of compound **18k**

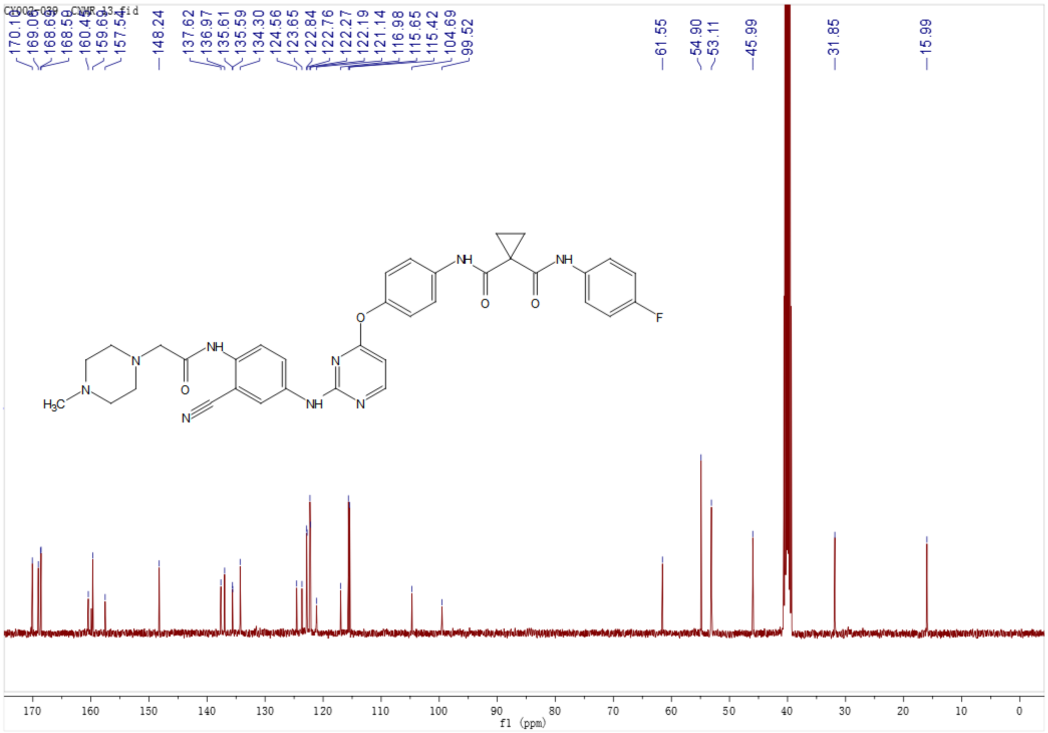

S60: ^{13}C-NMR of compound **18k**

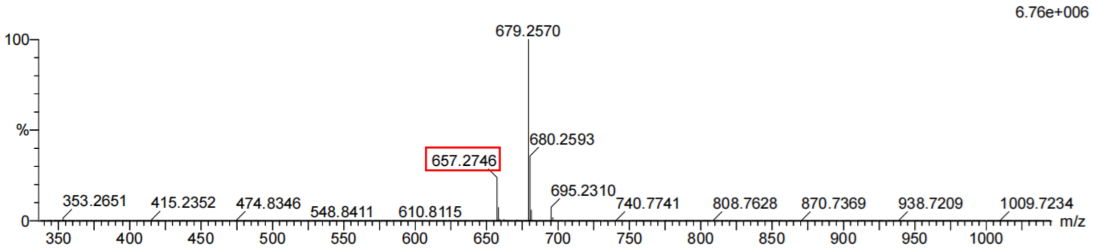

S61: HRMS of compound **18l**

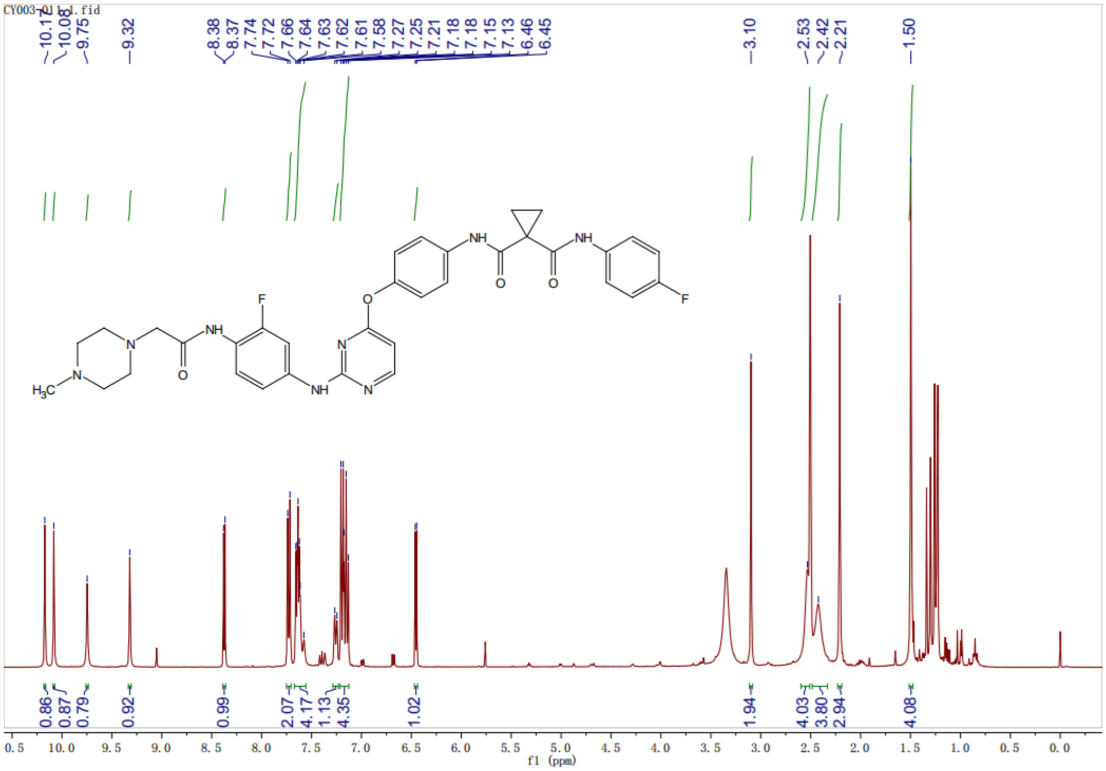

S62: ^{1}H-NMR of compound **18l**

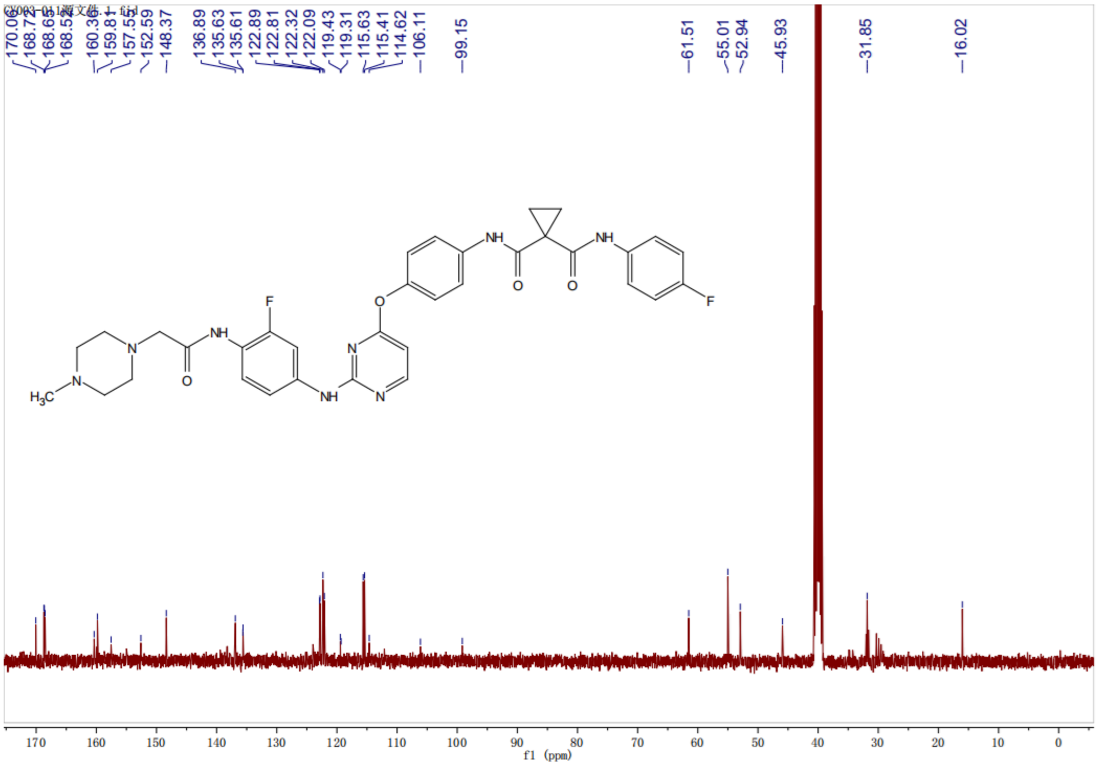

S63: ^{13}C-NMR of compound **18l**

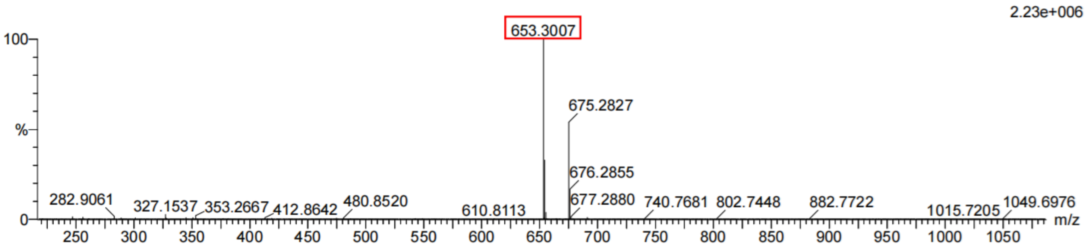

S64: HRMS of compound **18m**

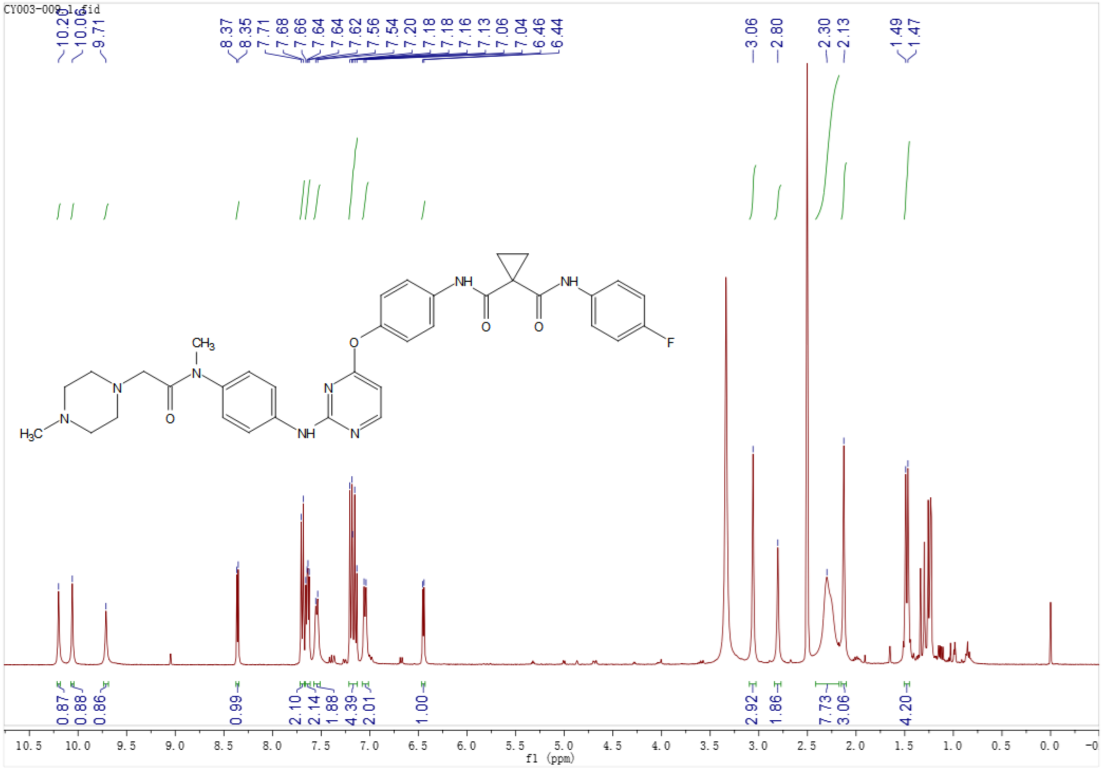

S65: ^{1}H-NMR of compound **18m**

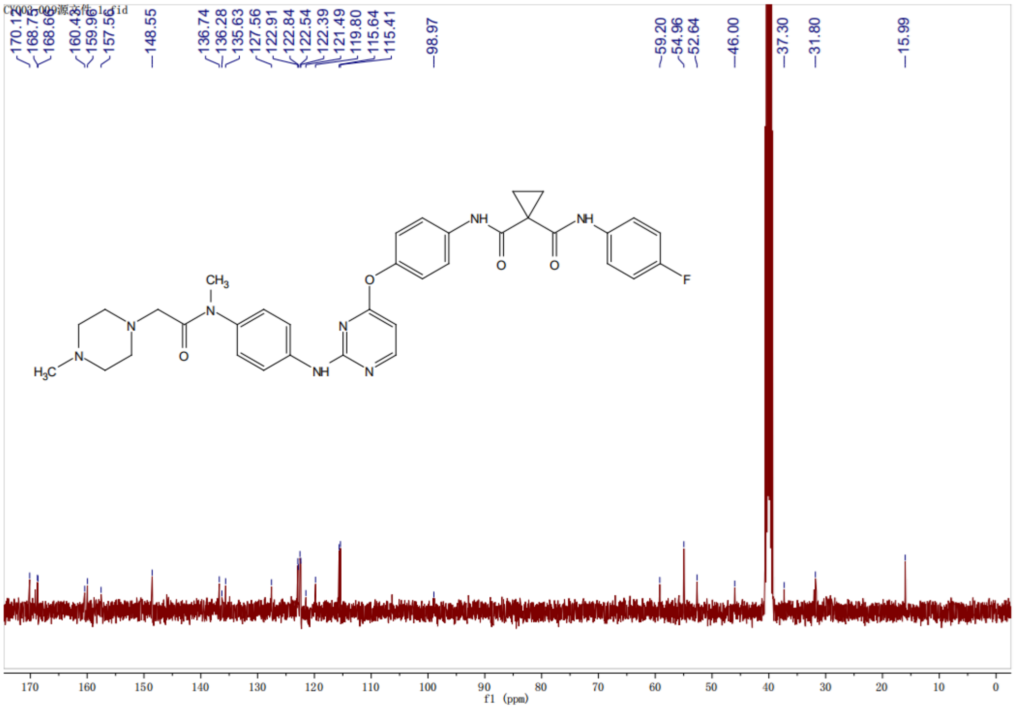

S66: ^{13}C-NMR of compound **18m**

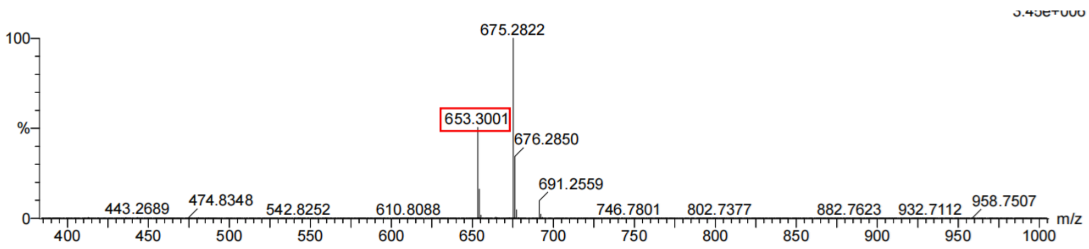

S$_{67}$: HRMS of compound **18n**

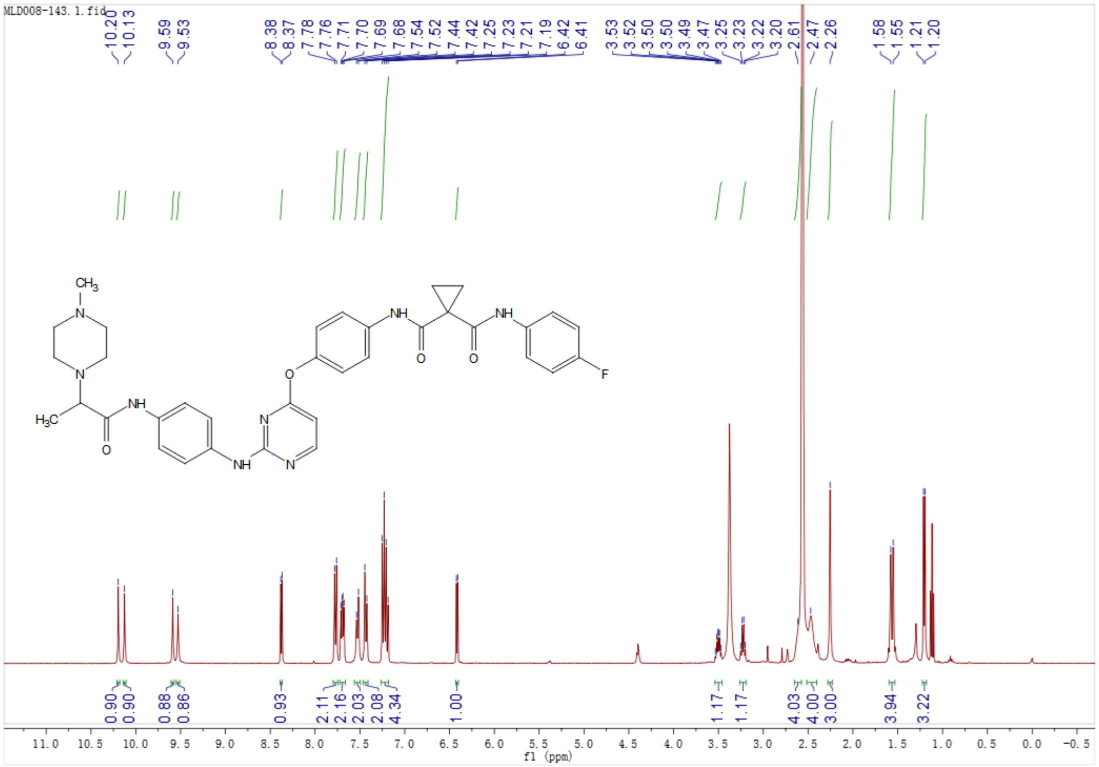

S$_{68}$: ^{1}H-NMR of compound **18n**

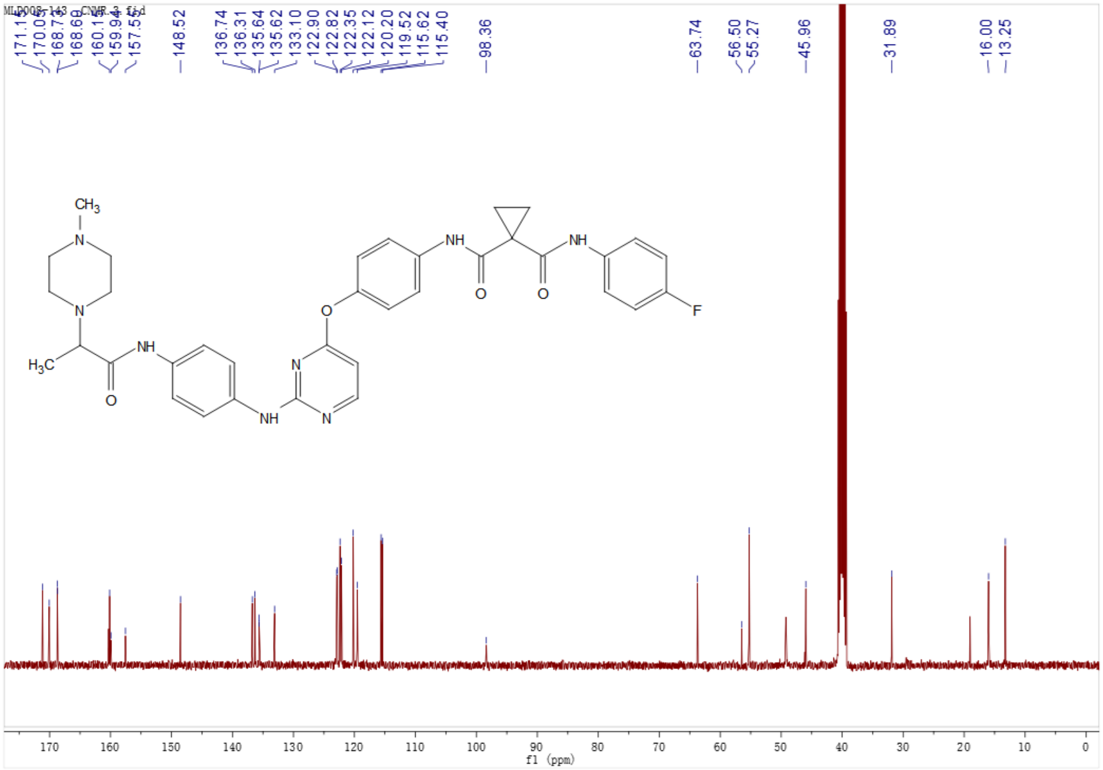

S$_{69}$: ^{13}C-NMR of compound **18n**

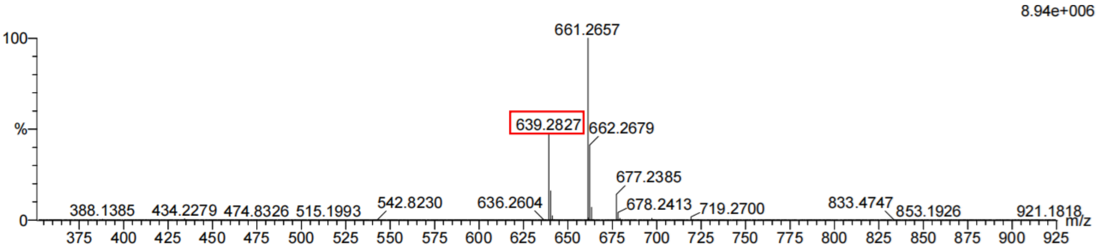

S$_{70}$: HRMS of compound **18o**

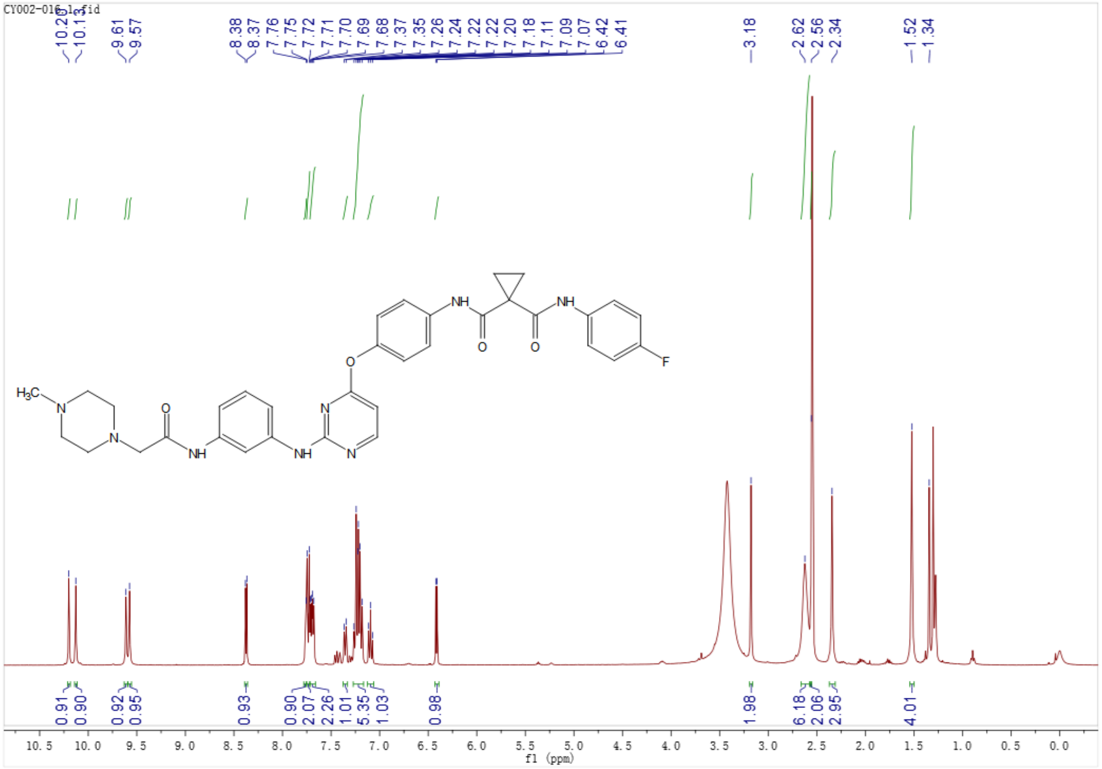

S$_{71}$: ^{1}H-NMR of compound **18o**

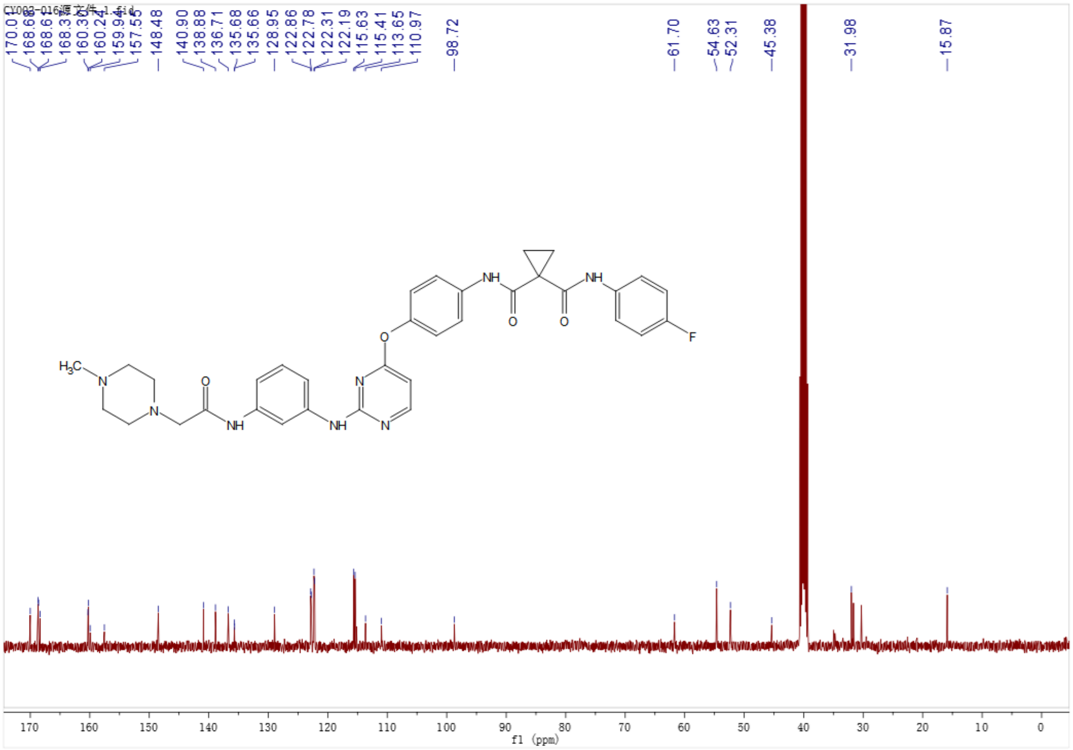

S$_{72}$: ^{13}C-NMR of compound **18o**
